# Genomic insights into the origin, domestication and diversification of *Brassica juncea*

**DOI:** 10.1038/s41588-021-00922-y

**Published:** 2021-09-06

**Authors:** Lei Kang, Lunwen Qian, Ming Zheng, Liyang Chen, Hao Chen, Liu Yang, Liang You, Bin Yang, Mingli Yan, Yuanguo Gu, Tianyi Wang, Sarah-Veronica Schiessl, Hong An, Paul Blischak, Xianjun Liu, Hongfeng Lu, Dawei Zhang, Yong Rao, Donghai Jia, Dinggang Zhou, Huagui Xiao, Yonggang Wang, Xinghua Xiong, Annaliese S. Mason, J. Chris Pires, Rod J. Snowdon, Wei Hua, Zhongsong Liu

**Affiliations:** 1 College of Agronomy, Hunan Agricultural University, Changsha, China; 2 Collaborative Innovation Center of Grain and Oil Crops in South China, Hunan Agricultural University, Changsha, China; 3 Oil Crops Research Institute of the Chinese Academy of Agricultural Sciences, Key Laboratory of Biology and Genetic Improvement of Oil Crops, Ministry of Agriculture and Rural Affairs, Wuhan, China; 4 Novogene Bioinformatics Institute, Beijing, China; 5 Guizhou Institute of Oil Crops, Guizhou Academy of Agricultural Sciences, Guiyang, China; 6 Hunan Key Laboratory of Economic Crops Genetic Improvement and Integrated Utilization, School of Life Science, Hunan University of Science and Technology, Xiangtan, China; 7 Xinjiang Academy of Agricultural Sciences, Urumqi, China; 8 Department of Plant Breeding, Justus Liebig University Giessen, Giessen, Germany; 9 Division of Biological Sciences, University of Missouri, Columbia, MO USA; 10 Department of Ecology and Evolutionary Biology, University of Arizona, Tucson, AZ USA; 11 College of Life Sciences, Resources and Environment Sciences, Yichun University, Yichun, China; 12 Plant Breeding Department, University of Bonn, Bonn, Germany

**Keywords:** Genomics, Plant breeding

## Abstract

Despite early domestication around 3000 BC, the evolutionary history of the ancient allotetraploid species *Brassica juncea* (L.) Czern & Coss remains uncertain. Here, we report a chromosome-scale de novo assembly of a yellow-seeded *B. juncea* genome by integrating long-read and short-read sequencing, optical mapping and Hi-C technologies. Nuclear and organelle phylogenies of 480 accessions worldwide supported that *B. juncea* is most likely a single origin in West Asia, 8,000–14,000 years ago, via natural interspecific hybridization. Subsequently, new crop types evolved through spontaneous gene mutations and introgressions along three independent routes of eastward expansion. Selective sweeps, genome-wide trait associations and tissue-specific RNA-sequencing analysis shed light on the domestication history of flowering time and seed weight, and on human selection for morphological diversification in this versatile species. Our data provide a comprehensive insight into the origin and domestication and a foundation for genomics-based breeding of *B. juncea*.

## Abstract (web summary)

A chromosome-scale de novo assembly of a yellow-seeded *Brassica juncea* genome and population analyses of 480 accessions from 38 countries provide insights into the origin, domestication history and morphological diversification of *B. juncea*.

## Main

*Brassica juncea* (L.) Czern & Coss is a diverse and important agricultural species^1^. An allotetraploid (AABB, 2*n* = 36), *B. juncea* derived from interspecific hybridization between the diploid progenitors *Brassica rapa* (AA, 2*n* = 20) and *Brassica nigra* (BB, 2*n* = 16)^2^. Four subspecies have been proposed based on crop use and morphology: *juncea* (seed mustard), *integrifolia* (leaf mustard), *napiformis* (root mustard) and *tumida* (stem mustard)^3^*. B. juncea* has a wide geographic range as native plants, adapted crops and introduced weeds, spanning the continents of Asia, Europe, Africa, America and Australia^4^. *B. juncea* is an important oilseed crop in India, Bangladesh, China and Ukraine, and is recently also gaining importance in Canada and Australia^5^. Meanwhile, it is grown as a condiment in Europe, North America, Argentina and China. Root mustard is distributed in Mongolia and northeastern China, whereas leaf mustards are most common in China and Southeast Asia^5,6^.

*Brassica juncea* is regarded as one of the earliest domesticated plants, with mustard mentioned as a condiment in Sanskrit and Sumerian texts from as early as 3,000 BC^7^. However, its center of origin is uncertain. Based on biogeographic explorations, Vavilov^8^ proposed Central Asia (Afghanistan and its contiguous regions) as the primary center of the origin of *B. juncea*, and Asia Minor, central/western China and eastern India as secondary centers of diversity. By contrast, many investigators^9–12^ proposed that *B. juncea* first evolved in the Middle East where its progenitor species, *B. rapa* and *B. nigra*, are sympatric. Whether *B. juncea* has a monophyletic or polyphyletic origin is controversial. Early morphological studies proposed a single origin^13,14^, whereas more detailed investigations implementing chemotaxonomy^15^, nuclear DNA markers^16,17^ and chloroplast (CP) genomic markers^18^ suggested a polyphyletic origin. Recently, a single origin was proposed once again based on genome re-sequencing, using 109 *B. juncea* accessions^19,20^. More comprehensive studies would accelerate our understanding of either the center of origin of *B. juncea*, or the number of origin and/or domestication events that gave rise to this important crop species.

Population genomics offers an opportunity to improve our understanding of the origin and domestication of crop plants^21^. To obtain a comprehensive overview of the origin, domestication and diversification of *B. juncea*, we first generated a chromosome-scale de novo assembly of a genome of the yellow-seeded *B. juncea* var. Sichuan Yellow (SY), using PacBio long reads combined with BioNano optical mapping and Hi-C chromatin interaction maps. Subsequently, we re-sequenced 480 *B. juncea* accessions from 38 countries, leading to the identification of around 4.53 million SNPs and 0.97 million insertion–deletion polymorphisms (InDel; <50 bp). Our combined analysis of CP, mitochondrial (MT) and nuclear genome data supports a single origin of *B. juncea* in West Asia, followed by at least three independent domestication events, and the evolution of new forms through spontaneous gene mutations and introgressions during its eastward spread. We furthermore scanned for selective sweeps, performed genome-wide association studies (GWAS) for flowering time and seed weight, and illuminated the domestication history and artificial selection of genes implicated in morphological diversification among diverse *B. juncea* subspecies. Our results provide a comprehensive picture of the origin and domestication history of this versatile and economically important crop species.

## Results

### Chromosome-scale genome of a yellow-seeded *Brassica juncea*

**Fig. 1:**
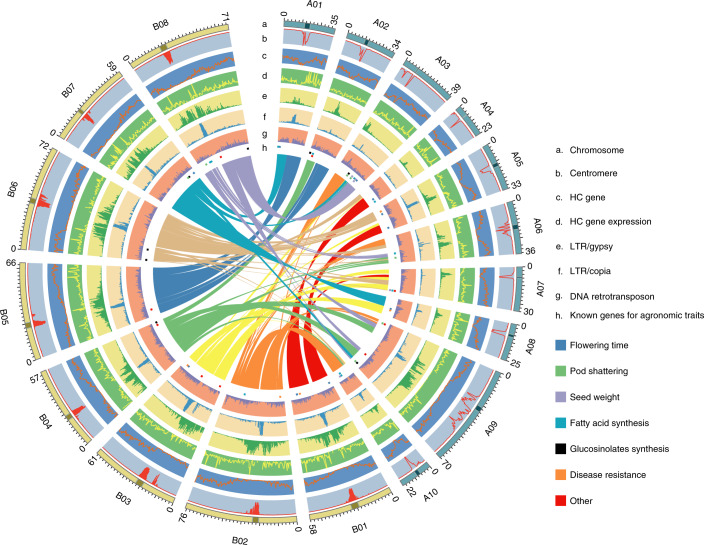
Chromosomal features and functional and synteny landscape of the yellow-seeded *B. juncea* var. SY genome. Tracks from outer (**a**) to inner (**h**) rings indicate the following: **a**, Chromosome size with units in Mb; **b**, Density of centromere-specific repeats in 5-Mb bins; **c**, Density of HC genes in 5-Mb bins; **d**, Expression of HC genes from nine tissues, calculated as the fragments per kilobase of transcript per million mapped reads (FPKM) in 5-Mb bins and normalization of FPKM by log_10_(FPKM + 1). **e**, LTR/Gypsy density (Gypsy length/5 Mb). **f**, LTR/Copia density (Copia length/5 Mb). **g**, DNA retrotransposon density (DNA retrotransposon length/5 Mb). **h**, Location of known genes (Supplementary Table 5) for major phenotypic traits. Lines in the center linking different chromosomal regions show the syntenic relationships between the A and B subgenomes.

Yellow-seeded *B. juncea* is grown widely as a condiment and oilseed. For de novo assembly of the SY genome, we integrated four sequencing and assembly technologies: PacBio long-read sequencing, Illumina short-read sequencing, BioNano optical mapping and Hi-C data (Supplementary Fig. 1 and Supplementary Table 1). The SY genome size was estimated to be 1056.53 Mb by *k*-mer analysis (Table 1 and Supplementary Fig. 2), close to the 1,068 Mb estimated by flow cytometry^22^. PacBio reads (~93×) were first assembled using FALCON^23^, followed by contig correction using Illumina reads (~130×) to generate a V.1 assembly (Supplementary Table 2). Using 202-fold coverage of BioNano data, we then generated an optical consensus map, which was implemented to assemble 1,897 super-scaffolds with an N50 of 5.87 Mb (assembly V.2). These contigs were categorized and ordered into 18 chromosome-scale scaffolds using a 15,543-marker high-density linkage map (Supplementary Fig. 3a and Supplementary Table 3). Finally, we used Hi-C data to confirm the pseudo-chromosomes and manually adjusted 165 mis-joined contigs by Juicebox^24^ (Supplementary Fig. 3b,c and Supplementary Table 2). The final SY assembly captured 933.5 Mb of genome sequence, with 867.3 Mb (~92.9%) anchored into chromosomes (Fig. 1 and Supplementary Table 4), which is superior to previous assemblies of stem^19^ and Indian^25^ mustard in terms of genome size, contiguity and anchorage. We simultaneously assembled the CP (153,465 bp) and MT (219,803 bp) genomes of SY (Supplementary Figs. 4 and 5).

**Table 1:** Summary statistics for the *Brassica juncea* var. Sichuan Yellow genome assembly

Genomic feature	SY
Estimated genome size (Mb)	1056.53
Total assembly size (bp)	933,496,244
Longest scaffold (bp)	76,001,744
Scaffold N50 (bp)	59,341,207
Contig N50 (bp)	1,926,153
Missing bases (%)	4.76
Sequences anchored to chromosome (%)	92.91
Annotated protein-coding genes (*n*)	82,723
TE proportion (%)	50.36

The high quality of the SY assembly was validated (Methods) by BUSCO and CEGMA scores of more than 98.5% (Supplementary Table 6), by alignment of over 95% identity with 81 randomly selected BACs and 2,567 paired BAC-end sequences^26^ (Supplementary Fig. 6 and Supplementary Tables 7 and 8), by high long terminal repeat (LTR) Assembly Index (LAI)^27^ of 10.73 among the assembled *Brassica* genomes (Supplementary Table 9), by high consistency with our genetic and optical maps (Supplementary Figs. 3a and 7), by consistent syntenic gene ordering (Supplementary Fig. 8) using genome-ordered graphical genotypes^28^, and by the good collinearity of SY to those of *B. rapa*^29^ and *B. nigra*^30^ and other previously reported *Brassica* genomes^19,25,31^ (Supplementary Fig. 9).

**Extended Data Fig. 1:**
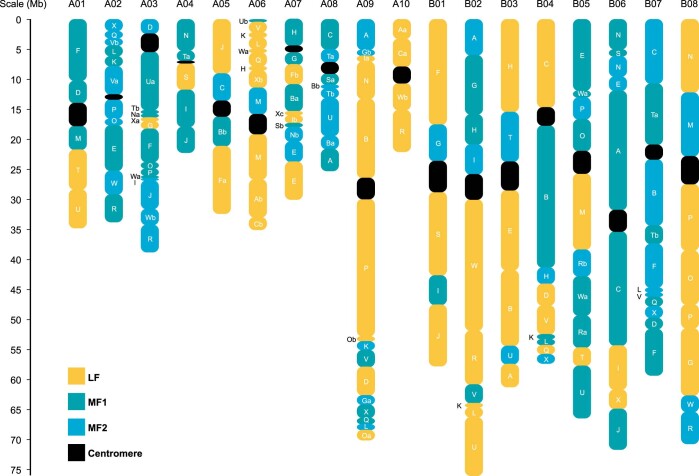
Distribution of genomic blocks along the eighteen chromosomes of the *Brassica juncea* var. Sichuan Yellow genome. Genome blocks on eighteen chromosomes were assigned to the subgenomes LF (orange), MF1 (dark cyan), and MF2 (deep sky blue). The 24 conserved genomic blocks are defined and labelled from A to X (colored) based on the syntenic relationship of the *B. juncea* and *A. thaliana* genomes. The centromeres in the SY genome are shown as black. The orientation of chromosomes is according to international standards such that the centromeres are toward the top of the chromosome.

The SY assembly contained 50.36% TEs (Table 1 and Supplementary Table 10), slightly more than the published genomes of *B. juncea* T84-66 (43.5%)^19^ and Varuna (45.8%)^25^ and *B. rapa* (37.51%)^32^, but less than *B. nigra* (53.73%)^30^. In accordance with previous *Brassica* genomes^19,25,29–33^, LTR/*gypsy* retroelements were the predominant TE family (Supplementary Table 10). We distinguished the chromosomal centromeric from pericentromeric regions by specific repeats^30,34–37^ (Fig. 1, Extended Data Fig. 1 and Supplementary Table 11), and remarkably lower recombination frequencies (Supplementary Fig. 3a). The centromere and pericentromeric regions were enriched for LTR/*copia* and LTR/*gypsy* elements, respectively (Fig. 1 and Supplementary Table 12).

Among 92,878 predicted gene models (Supplementary Note and Supplementary Table 13), 95.5% were functionally annotated in public databases (Supplementary Table 14). Alignment to known proteins and expression in at least one tissue type showed 82,723 gene models were high-confidence (HC) genes (Supplementary Table 15), with an average coding sequence length of ~1.13 kb and an average of five exons per gene, similarly to predictions in other *Brassica* genomes (Supplementary Table 13). A total of 5,756 genes (6.96% of the HC genes) encoded putative transcription factors belonging to 58 different families (Supplementary Table 16). We also identified 2,525 tRNAs, 8,363 rRNAs, 1,951 microRNAs and 4,691 small nuclear RNAs (Supplementary Table 17).

### Population structure and genomic variation

**Fig. 2:**
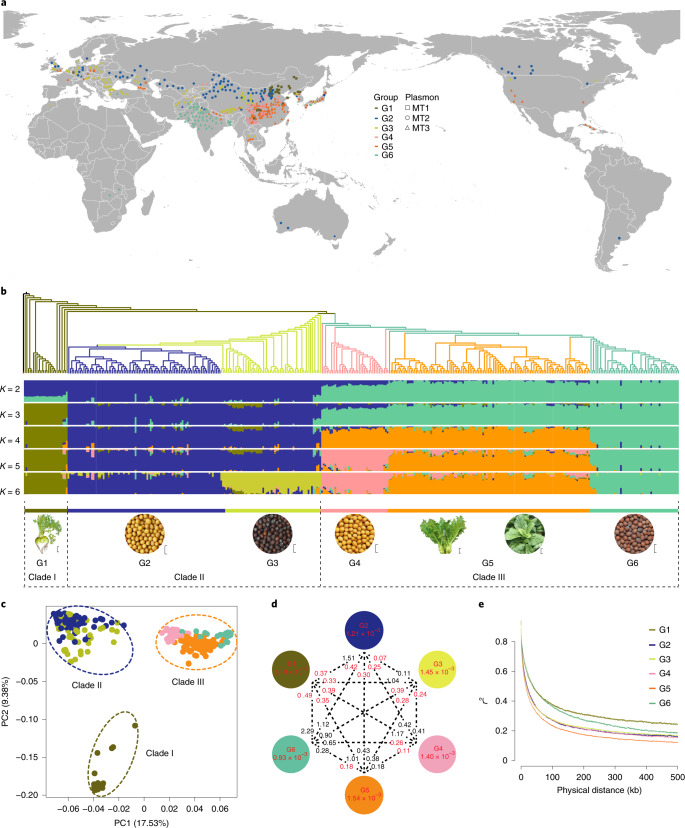
Geographic distribution, population structure and genomic diversity of *Brassica juncea* accessions. **a**, Geographic distributions of 480 *B. juncea* accessions. The geographic map was drawn using R ggplot2. **b**, The maximum-likelihood phylogeny of 390 *B. juncea* accessions with over 60% genetic components to the group and model-based clustering with *K* from 2 to 6. The five other *Brassicaceae* species used to root the phylogenetic tree are shown as a single branch. Branch colors indicate different groups based on the population structure. Scale bars, 5 cm for G1 and G5; 5 mm for G2, G3, G4 and G6. **c**, PCA plots showing three divergent clades of 390 *B. juncea* accessions. **d**, Nucleotide diversity (*π*), population divergence (*F*_ST_) and genetic distance (*D*) across the six groups. The value in each circle represents a measure of nucleotide diversity for each group; values in red on each line indicate pairwise population divergence between groups, while values in black on each line indicate pairwise genetic distances among groups. **e**, Group-specific LD decay plots.

To explore genetic variation in *B. juncea*, we re-sequenced 480 accessions representing the four subspecies from 38 countries (Fig. 2a and Supplementary Table 18) with an average depth of 15× and 97.7% of the SY genome. Using this dataset, we identified 4,529,618 high-quality SNPs and 967,266 InDels (Supplementary Table 19) based on four parameters (Methods), corresponding to 4.85 SNPs and 1.04 InDels per kb (Supplementary Table 20). A total of 946,661 SNPs (20.9%) and 50,955 InDels (5.27%) were located in coding regions. Among them, 345,138 SNPs (7.62%) caused codon changes, elongated transcripts or premature stop codons, while 27,420 InDels (2.83%) led to frameshift mutations. The SNP distribution varied across the genome depending on genome context and gene density, but was generally higher toward the telomeric chromosome regions (Supplementary Fig. 10). The A subgenome of *B. juncea* had higher nucleotide diversity (*π* = 2.05 × 10^−3^) than the B subgenome (*π* = 1.45 × 10^−3^; Supplementary Fig. 11). Moreover, linkage disequilibrium (LD) decayed faster in the A subgenome than in the B subgenome (Supplementary Fig. 12), indicating a higher degree of genetic recombination in the A subgenome of *B. juncea*.

Next, we investigated the genetic structure of the *B. juncea* population for clusters (*K*) from 2 to 10 based on 4.53 million SNPs among the 480 *B. juncea* accessions. When *K* = 6, clusters maximized the marginal likelihood (Supplementary Fig. 13). To better clarify the relationships within the population, 90 genetically admixed accessions with main genetic components of less than 60% were excluded from further analysis. Both phylogenetic and principal component analyses (PCAs) of the remaining 390 samples indicated three distinct clades (Fig. 2b,c). Clade І consisted only of root mustard from Northeast Asia. Clade II consisted of seed mustard from West Asia, Central Asia and Northwest China along the Steppe Route, a trans-Eurasian trading route predating the Silk Road^38^. Clade III included oilseed and vegetable mustards from the Indian subcontinent and southern China, corresponding to the South Silk Road connecting East and Central Asia^39^.

Our phylogenetic and genetic clustering analyses resolved six *B. juncea* genetic groups (G1–G6), which largely corresponded to morphologically distinct crops (Supplementary Fig. 14 and Supplementary Table 21). G1, the root mustard group, showed the slowest LD decay, especially in the B subgenome, and strong genetic divergence from the other five groups (pairwise *F*_ST _≥ 0.33; Fig. 2e and Supplementary Tables 22 and 23). G2 comprised yellow-seeded mustard, and almost 60% of the G2 accessions with known geographic origins were from northwestern China; other G2 accessions sourced from the former Soviet Union, Canada and Europe were documented introductions from China^40–42^. G3 spanned wide geographic origins from Tibet, central and western Asia to Europe. G3 clustered close to but distinctly from G2 (*F*_ST_ = 0.07; Fig. 2d). G4 comprised mainly accessions from southwestern China and clustered closest to the G5 group. The G5 group, including 96 leaf, 14 stem and 10 seed mustards originating from southern China to Japan^43^ and the USA^9,41^, showed the highest nucleotide diversity (*π* = 1.54 × 10^−3^) and the greatest LD decay (Fig. 2d,e). The 59 accessions forming the group G6 were almost all from South Asia. G6 showed a similarly slow LD decay to G1, and it also exhibited the lowest nucleotide diversity (*π* = 0.93 × 10^−3^), consistent with a narrow genetic base of Indian mustard^44^. All genotypes belonging to G2 and G3 in Clade II and to G4 and G6 in Clade III are grown for seed use, whereby G2 and G3 differentiate less strongly from G4 (pairwise *F*_ST_ = 0.25 and 0.24, respectively) than from G6 (pairwise *F*_*ST* _= 0.42 and 0.39, respectively; Fig. 2e and Supplementary Table 22).

### Domestication and spread of *Brassica juncea*

**Fig. 3:**
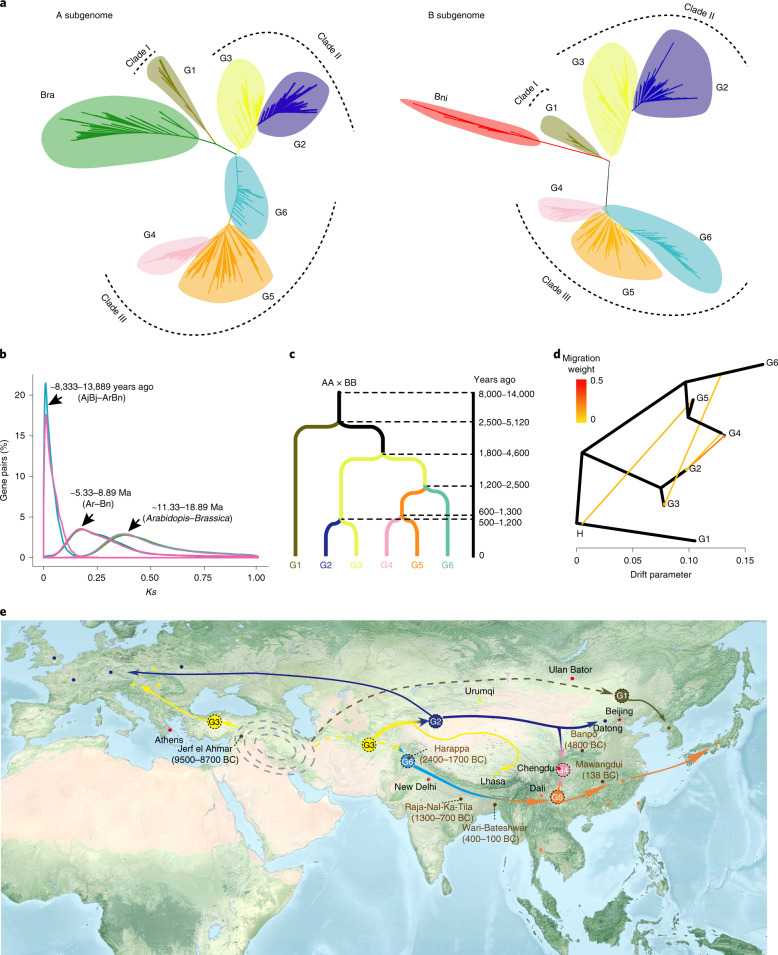
Speciation and demographic history of *Brassica juncea*. **a**, Maximum-likelihood phylogenies of the subgenomes of 390 *B. juncea* accessions compared to 68 *B. rapa* accessions (left), and 11 *B. nigra* accessions (right). **b**, Estimates of molecular divergence between *B. juncea* (AjBj) and its pseudo-ancestor (ArBn, pooled by two progenitors, *B. rapa* and *B. nigra*). **c**, Divergence time for six groups was estimated using SMC++. **d**, Detection of gene flows among *B. juncea* groups by TreeMix analysis. Arrows represent the direction of migrations. Horizontal branch length is proportional to the amount of genetic drift that has occurred on the branch. Scale bar shows ten times the average standard error of the entries in the sample covariance matrix. **e**, Putative spread routes of *B. juncea*. Archaeological evidence showing that seed cakes or carbonized mustard seeds were excavated from Jerf el Ahmar (9500–8700 BC)^54^, Banpo site (about 4800 BC)^55^, Harappa (2400–1700 BC)^59^, Raja-Nal-ka-Tila site (1300–700 BC)^60^, Wari-Bateshwa (400–100 BC)^61^ and Mawangdui site (about 138 BC)^64^. The geographic map was adapted from NASA (https://visibleearth.nasa.gov/images/147190/explorer-base-map/147191w/). Ma, million years ago.

To delineate domestication and spread, we further constructed A and B subgenome phylogenies of *B. juncea* and its progenitors (Supplementary Table 24). Both subgenome phylogenetic trees confirmed six groups of *B. juncea* and that the G1 group was the closest to the progenitor species, although G4 and G6 had the opposite positions (Fig. 3a and Supplementary Figs. 15 and 16). These nuclear phylogenies support the hypothesis that *B. juncea* originated monophyletically^19^.

**Extended Data Fig. 2:**
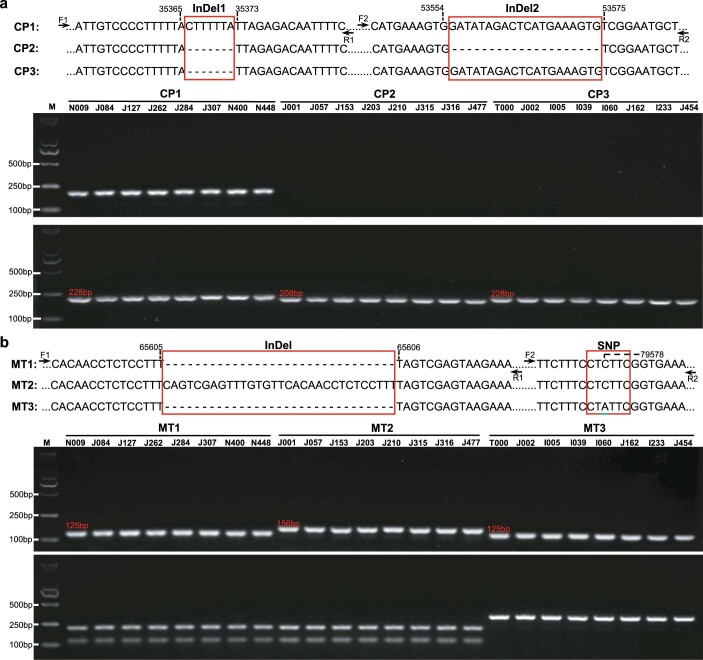
Three types of *Brassica juncea* chloroplast and mitochondrial genomes. **a**, Three *B. juncea* chloroplast genome types were identified by sequence alignment. PCR validation of the two InDels in the chloroplast genomes of *B. juncea* accessions. **b**, Three *B. juncea* mitotypes were shown by sequence alignment. PCR validation of the InDel and the SNP in the mitochondrial genomes of *B. juncea* accessions. The amplified DNA was treated with the restriction enzyme *Ear*I. All the PCR experiments were repeated independently for three times with similar results. The primers used for PCR were listed in Supplementary table 42. Source data for the gels were provided as a Source Data file. Source data

We assembled 478 CP and 10 MT genomes to study cytoplasmic relationships between *B. juncea* and its progenitors (Supplementary Tables 18 and 25). Based on the assembled CP genomes, we found two InDel variants and divided the *B. juncea* CP genomes into three types (CPs 1–3; Extended Data Fig. 2a and Supplementary Table 18). Meanwhile, we classified the MT genomes into three types (MTs 1–3) using an InDel and a SNP locus^45^ (Extended Data Fig. 2b and Supplementary Table 18). These three MT types corresponded to the three specific CP classifications, and were subsequently named plasmotypes I–III. All G1 accessions carried plasmotype І, whereas all G6 and most (94.2%, 113/120) G5 accessions harbored plasmotype III. The remaining three groups contained all three plasmotypes, with plasmotype II predominating (G2 91.3%, G3 71.2%, G4 70.0%; Supplementary Table 21). In the CP phylogeny, most (467/478) of the *B. juncea* accessions were rooted in the *B. rapa* lineage (Supplementary Fig. 17), consistent with the conclusion that *B. rapa* is the maternal ancestor of *B. juncea*^46,47^. CP and MT phylogenies (Supplementary Figs. 17 and 18) and PCR analysis (Extended Data Fig. 2) indicated that plasmotype І of *B. juncea* descended from *B. rapa* and evolved into plasmotype II and III via insertion/deletions and a base substitution. From the perspective of cytoplasmic inheritance, *B. juncea* shows a single origin.

The progenitor species of *B. juncea* are sympatric in the Middle East^48^. Wild *B. juncea* forms have been observed to grow on the plateaus in Asia Minor and southern Iran^10,49–52^. The group G3, including Turkish accessions, possessed not only all three plasmotypes (Fig. 2a and Supplementary table 21) but also higher nucleotide diversity (Fig. 2d), implying that the place where the G3 accessions were collected is a plausible center of origin. Collectively, these data support that *B. juncea* most likely originated in West Asia (the Middle East).

Importantly, we estimated that *B. juncea* formed ~8,000–14,000 years ago by natural hybridization between both progenitors (Fig. 3b). A demographic history model of the *B. juncea* groups favors at least three independent evolutionary routes (Fig. 3c). Four gene flows were detected among the six groups by Treemix and *D*-statistic analyses: from root mustard (G1) to leafy mustard (G5), from Indian mustard (G6) to West and Central Asia mustard (G3), from northwestern China (G2) to southwestern China yellow-seeded mustard (G4) and, with a lower weight, in the reciprocal direction from G4 to G2 (Fig. 3d and Supplementary Table 26).

Root mustard first diverged from wild *B. juncea*, approximately 2,500–5,120 years ago (Fig. 3c). We speculate that root mustard was domesticated in Mongolia and northeastern China according to its current geographic distribution and historical records^53^, although how it spread into East Asia remains elusive (Fig. 3e). Additionally, wild *B. juncea* was domesticated into the seed mustard (G3), and a diverse range of *B. juncea* accessions developed (Fig. 3c,e and Supplementary Table 18). The G3 mustard spread eastward from northern Afghanistan along the Steppe Route and entered Tibet via the Hexi corridor. During the dissemination process of G3, a new yellow-seed mustard (G2) evolved about 500 years ago from spontaneous gene mutations^56,57^, probably in Xinjiang^58^ (Fig. 3e). In parallel, the G3 mustard spread from southern Afghanistan into the Indian subcontinent^12^ where it was domesticated into Indian mustard (G6), which is supported by archaeological excavations^59^. Indian mustard then spread eastward^60,61^ to form a new type of broad-leaf mustard (var. *rugosa*)^13^, probably around 300 BC^62^. These broad-leaf mustards spread further east into southwestern China, where they were grown as vegetables and oilseed before the sixth century AD^63^. Historical records documented the subsequent derivation of stem mustard from broad-leaf mustard in the Sichuan Basin in the eighteenth century^6^. Accordingly, we observed very low genetic diversity in stem mustard and a closer relationship to leaf mustard (G5) than G4 accessions from the same geographic region (Supplementary Table 27).

**Extended Data Fig. 3:**
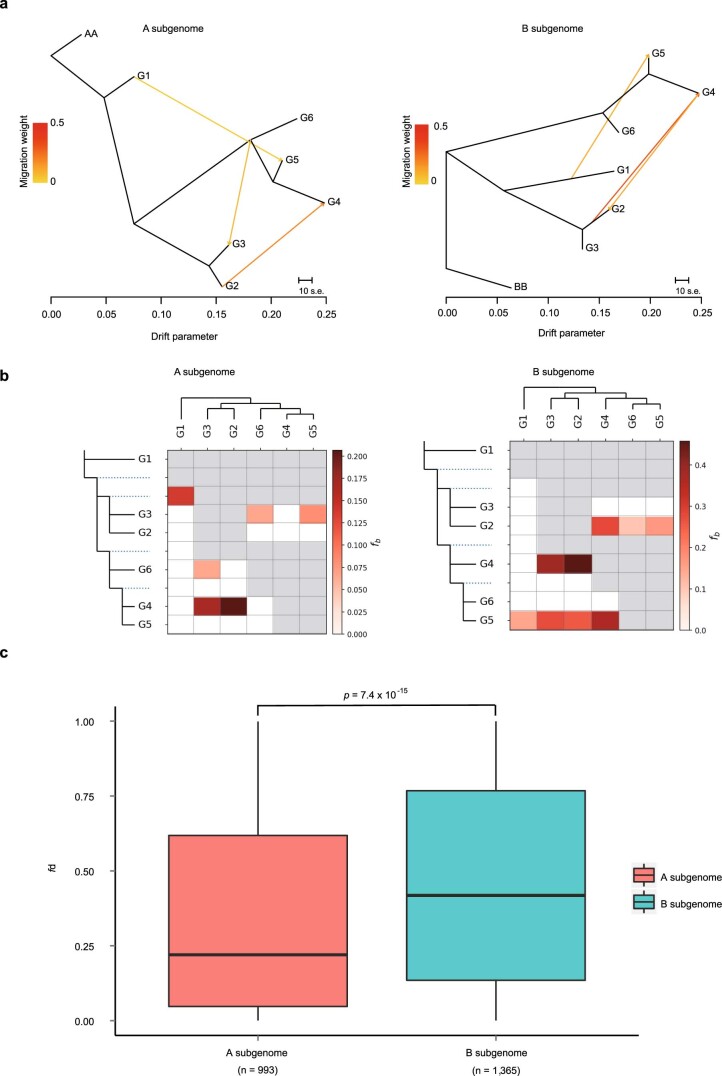
Estimation of introgressions among the six groups of *Brassica juncea*. **a**, Treemix analysis. Migration arrows are colored according to their weight. Horizontal branch length is proportional to the amount of genetic drift that has occurred on the branch. Scale bar shows ten times the average standard error of the entries in the sample covariance matrix. **b**, *f*-branch values. **c**, *fd* values from G2 to G4. The center lines in box plots indicate the median values, and the bottom and top edges of the box indicate the 25th and 75th percentiles, respectively. Whiskers extend to data no more than 1.5 times the interquartile range. *p*-value was calculated using two-sided *t*-test.

The G4 group inherited yellow-seed color and plasmotype II from G2, and early maturity from G5. Migration weight, *f-*branch and *f*_d_ values showed more genetic components were introgressed into the B subgenome than into the A subgenome from G2 to G4 (Extended Data Fig. 3), which can explain the opposite position of G4 and G6 in the A and B subgenome phylogenies (Fig. 3a). The proportions of introgressed fragments from G2 detected in the G4 accessions varied from 0.07 to 0.26, with an average of 0.159 (Supplementary Fig. 19 and Supplementary Table 28). The five largest introgressed genomic blocks (relative IBD rate > 0.7; Methods) included the regions from 49.8 to 50.8 Mb on chromosome A09 and from 39.8 to 41.8 Mb on chromosome B08, which carry *Arabidopsis thaliana* *TT8* (TRANSPARENT TESTA 8) orthologous genes (BjuA09g45700S and BjuB08g18790S) that are nonfunctional in yellow-seed *B. juncea*^56,57^. Therefore, we concluded that G4 is a genetic admixture from the natural hybridization of G2 with G5, implying that the combination of gene mutations by natural hybridization played a significant role in the domestication and spread of yellow-seeded *B. juncea*.

### Ecogeographic adaptation of *Brassica juncea* flowering time

We observed flowering time variation across 390 *B. juncea* accessions grown under four contrasting environments: 94 to 194 d in Guiyang, 71 to 200 d in Xiangtan, 29 to 78 d in Kunming and 25 to 65 d in Urumqi (Supplementary Fig. 20 and Supplementary Table 29). The flowering time of 390 accessions was positively correlated across different environments (*r*^2 ^= 0.46 to 0.95; Supplementary Fig. 21). The broad-sense heritability of flowering time reached 0.74 (Supplementary Table 29). Most of the root mustards and some leaf mustards did not flower in Kunming, indicating vernalization failure due to insufficiently low temperatures.

**Fig. 4:**
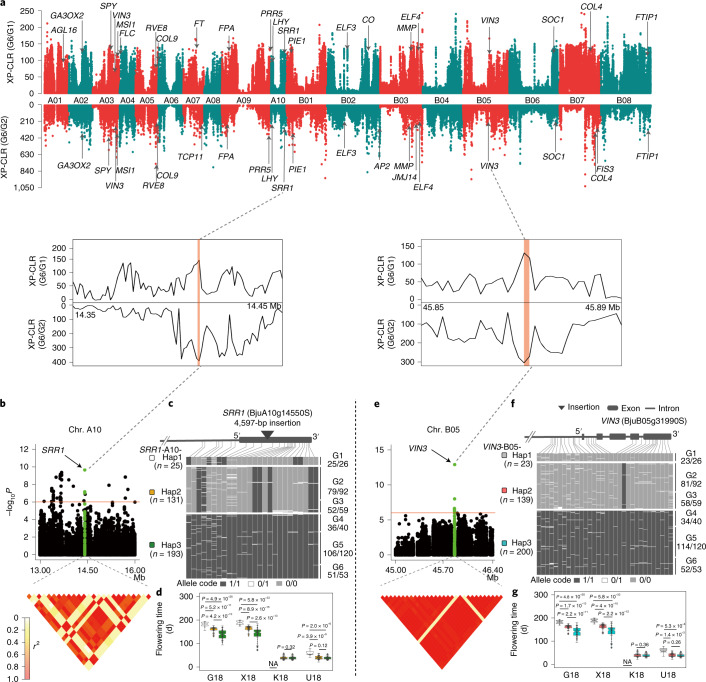
Genome-wide screening of selective sweeps and GWAS for flowering time in *Brassica juncea*. **a**, Genome-wide distribution of selective sweeps identified through comparisons between G1 or G2 with G6 using XP-CLR (cross-population composite likelihood-ratio test) values (sliding window = 10 kb, step = 1 kb). The flowering time candidate genes in the selective regions are labeled. **b**,**e**, Local Manhattan plot showing the 14.35–14.45 Mb and 45.85–45.89 Mb regions on chromosomes A10 and B05, respectively. The green plots represent the position of these SNPs in *SRR1* (BjuA10g14550S) and *VIN3* (BjuB05g31990S). Two and five SNPs in the gene regions of *SRR1* and *VIN3* were significantly associated with flowering time, respectively. Heat maps spanning the SNP markers in LD with the most strongly associated SNPs in *VIN3* and *SRR*1 gene regions. The red lines indicate the significance threshold (−log_10_*P* = 6.0). **c**,**f**, Three haplotypes with a frequency greater than 0.01 were identified in the *SRR1* and *VIN3* gene regions, respectively. Box plot showed three haplotypes corresponding to flowering time in *SRR1* and *VIN3* gene regions, respectively. **d**,**g**, Box plots for flowering time based on the haplotypes (Hap.) for *SRR1* (**d**) and *VIN3* (**g**) under four different environments. Box edges represent the 0.25 and 0.75 quartiles, with the median values shown by bold lines. Whiskers extend to data no more than 1.5 times the interquartile range, and remaining data are indicated by dots. *P* values were calculated using two-sided *t*-tests. NA, data missing (G1 group did not flower in Kunming).

We identified 43 and 38 putative selective sweeps in G6/G1 and G6/G2, respectively, containing 63 flowering time candidate genes (Fig. 4a and Supplementary Table 30). Of these genes, 30 and 7 have known roles in the photoperiod and vernalization pathways, respectively. We also scanned selective sweeps for flowering time by comparing G1 with group G2, G3, G4 or G5 and identified 42 candidate genes for flowering time (Supplementary Fig. 22). Simultaneously, a total of 56 candidate genes showed significant association to flowering time across the four environments by GWAS analysis (Supplementary Fig. 23 and Supplementary Table 31). Of these genes, 12 also detected by the selective-sweep scan were investigated in more detail as potential contributors to domestication (Supplementary Fig. 24).

**Extended Data Fig. 4:**
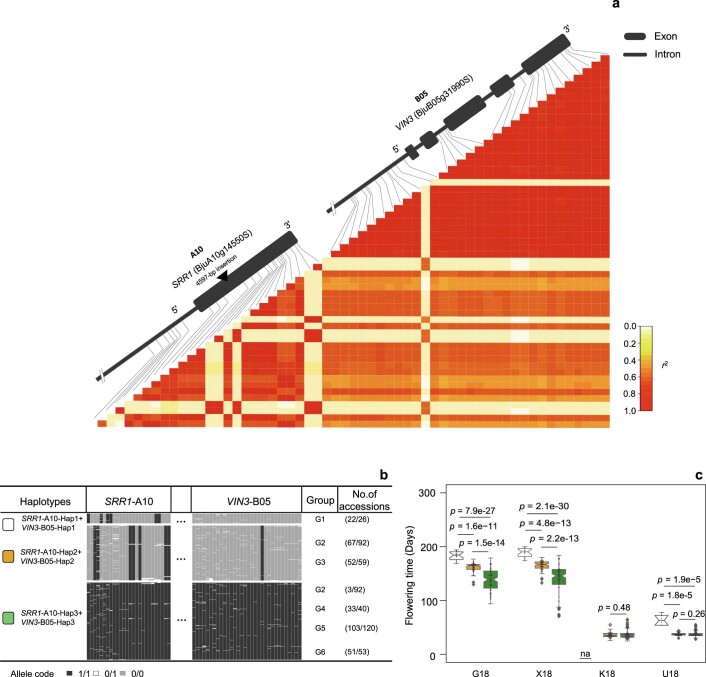
Co-evolution analysis of the flowering time genes *SRR1* (BjuA10g14550S) and *VIN3* (BjuB05g31990S) in *Brassica juncea*. **a**, LD analysis between *SRR1* and *VIN3* genes. **b**, The combinations of both *SRR1* and *VIN3* haplotypes (*SRR1*-A10-Hap1 + *VIN3*-B05-Hap1, *SRR1*-A10-Hap2 + *VIN3*-B05-Hap2, and *SRR1*-A10-Hap3 + *VIN3*-B05-Hap3). **c**, Boxplot showing comparison between these three haplotypes corresponding to accessions across four environments. Box edges represent the 0.25 and 0.75 quantiles with the median values shown by bold lines. Whiskers extend to data no more than 1.5 times the interquartile range, and remaining data are indicated by dots. *p*-value was calculated with two-sided *t*-test. na, data missing (G1 group did not flower in Kunming).

Notably, two SNPs in the region of BjuA10g14550S (*SRR1*, SENSITIVITY TO RED LIGHT REDUCED 1) and five SNPs in BjuB05g31990S (*VIN3*, VERNALIZATION INSENSITIVE 3) were found to be significantly associated with flowering time (Fig. 4b,e and Supplementary Table 31). SRR1 is a pioneer protein involved in the regulation of the circadian clock and phytochrome B signaling^65^, while *VIN3* is a crucial gene involved in vernalization^66^. We found strong LD between *SRR1* on chromosome A10 and *VIN3* on B05 (Extended Data Fig. 4a). The combinations of both *SRR1* and *VIN3* haplotypes were consistent with the haplotypes of either gene (Extended Data Fig. 4b,c). *SRR1*-A10-Hap1 and *VIN3*-B05-Hap1 were present in late-flowering or non-flowering accessions of the G1 group, which was domesticated in cold, long-day environments. *SRR1*-A10-Hap2 and *VIN3*-B05-Hap2 were present mostly in accessions from G2 and G3 with moderate flowering time. These seed mustard groups were domesticated under long-day conditions with large diurnal temperature variations (20–30 °C). Finally, *SRR1*-A10-Hap3 and *VIN3*-B05-Hap3 were present in the earliest-flowering accessions, mainly from G4, G5 and G6 (Fig. 4c,d,f,g and Supplementary Table 32). These results demonstrate the coevolution of *SRR1* and *VIN3* during the domestication of *B. juncea*, and support the conclusion that *B. juncea* underwent three independent domestication events.

Furthermore, a 4,597-bp insertion was found in the exon of *SRR1*. All *SRR1*-A10-Hap3 accessions have this insertion, whereas it is carried only by some (50/118) *SRR1*-A10-Hap2 accessions (Supplementary Fig. 25a,b). Comparing flowering time, we found that *SRR1*-A10-Hap2 accessions with the insertion flower earlier than those without the insertion, suggesting that this gene lost its function because of the premature termination codon produced by the insertion (Supplementary Fig. 25b,c). A 13-bp insertion in the third intron and 6-bp deletion in the fifth exon of *VIN3* were detected in *VIN3*-B05-Hap1 and *VIN3*-B05-Hap2 (Supplementary Fig. 26a). *VIN3*-B05-Hap3 accessions have the highest relative expression level and flower earliest, while *VIN3*-B05-Hap1 and *VIN3*-B05-Hap2 accessions flower latest and show a moderate, but not significantly different, gene expression level (Supplementary Fig. 26b) because these two haplotypes differ at only a single SNP (Supplementary Fig. 27).

In addition, we identified 15 genes significantly associated with flowering time by both GWAS and selective-sweep scan (Supplementary Table 33). These genes included transcription factors, SUVR and WD-40 repeat proteins, and gibberellic acid signaling, which warrant further investigation.

### Genetics of morphological diversification in *Brassica juncea*

Domestication and artificial selection of *B. juncea* imparted major morphotype changes, including the increase in seed size, root expansion and stem swelling. We aimed to identify selective sweeps and genomic regions associated with each of these traits in the *B. juncea* panel.

Seed size is a primary agronomic trait that contributes to seed yield in condiment and oilseed mustards. We observed significant variation in thousand seed weight (TSW), ranging from 0.29 to 2.48 g, 0.52 to 2.94 g, 0.66 to 3.16 g and 0.96 to 4.30 g across the four environments, respectively (Supplementary Fig. 21 and Supplementary Table 29). A high broad-sense heritability of 0.92 was calculated for TSW (Supplementary Table 29). Significant positive correlations were detected across the environments, with *r*^2^ values of 0.44–0.82 (Supplementary Fig. 21).

**Extended Data Fig. 5:**
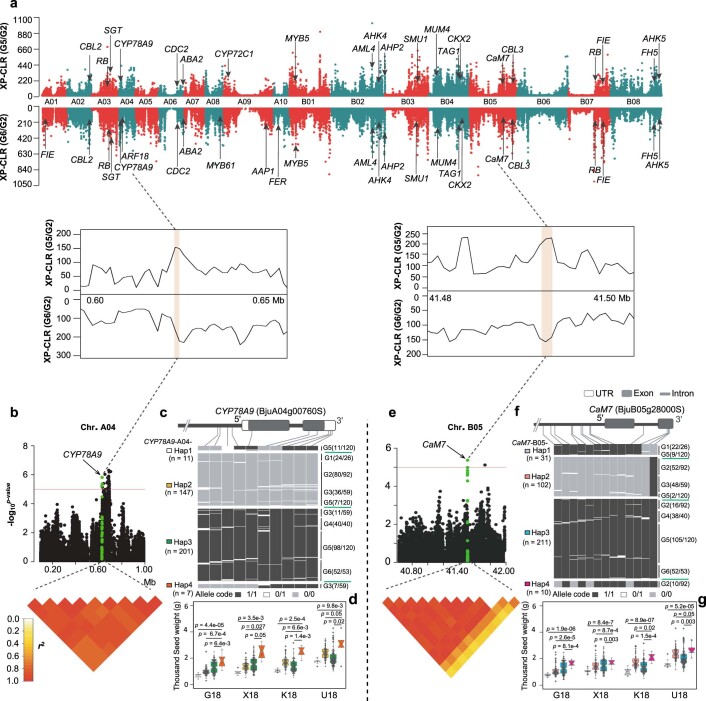
Genome-wide selective sweep scan and GWAS for seed weight in *Brassica juncea*. **a**, Genome-wide distribution of selective-sweep signals identified through comparisons between G5 or G6 with G2 using XP-CLR values (sliding window = 10 kb, step = 1 kb). The thousand seed weight candidate genes in the selection regions are labeled. **b** and **e**, Local Manhattan plot showing the 0.60 - 0.65 Mb and 41.48 - 41.50 Mb region on chromosomes A04 and B05, respectively. The green plots represent the position of these SNPs in *CYP78A9* (*BjuA04g00760S*) and *CaM7* (*BjuB05g28000S*). Three and one SNPs in *CYP78A9* and *CaM7* are significantly associated with thousand seed weight, respectively. The heatmaps span the SNP markers that show linkage disequilibrium (LD) with the most strongly associated SNPs. The grey dashed lines indicate the significance threshold (-log_10_^*p*^ = 5.0). **c** and **f**, Comparison of conserved SNPs specific to six groups in *CYP78A9* and *CaM7* gene region, respectively. Two haplotypes with frequency greater than 0.01 were identified in *CYP78A9* and *CaM7* gene region, respectively. **d** and **g**, Comparison in thousand seed weight between accessions of three haplotypes in *CYP78A9* and *CaM7* gene region, respectively. Box edges represent the 0.25 quantile and 0.75 quantile with the median values shown by bold lines. Whiskers extend to data no more than 1.5 times the interquartile range, and remaining data are indicated by dots. *p*-value was calculated with two-sided *t*-test.

We identified 33 and 51 putative selective sweeps in G5/G2 and G6/G2, respectively, which contained 65 candidate genes for TSW. Among these genes, 19 overlapped between G5/G2 and G6/G2 (Supplementary Table 34). We detected 22 significantly associated candidate genes using GWAS (Supplementary Fig. 28 and Supplementary Table 35), of which 7 were also detected by selective sweeps (Supplementary Fig. 28). The two genes detected by both approaches, BjuA04g00760S (*CYP78A9*, CYTOCHROME P450 78A9) and BjuB05g28000S (*CAM7*, CALMODULIN 7; Extended Data Fig. 5b,e and Supplementary Table 35), were previously shown to regulate seed weight in *Brassica* *napus*^67^ and *Gossypium hirsutum*^68^. Four haplotypes were detected in *CYP78A9*. *CYP78A9*-A04-Hap4 was present in 7 G3 accessions with the highest TSW, whereas *CYP78A9*-A04-Hap1 was present in 11 G5 vegetable accessions with the lowest TSW under four environments. *CYP78A9*-A04-Hap2 was mainly present in accessions from G1, G2 and G3, while *CYP78A9*-A04-Hap3 was present mainly in accessions from G4, G5 and G6. We also detected four haplotypes for *CAM7*. *CAM7*-B05-Hap1 corresponded to the G1 root mustard types with the lowest TSW, whereas *CAM7*-B05-Hap4 corresponded to 10 G2 oilseed accessions which had the highest TSW across environments. The accessions with *CAM7*-B05-Hap2 and *CAM7*-B05-Hap3 corresponded well to those with *CYP78A9*-A04-Hap2 and *CYP78A9*-A04-Hap3, respectively (Extended Data Fig. 5f,g and Supplementary Table 36).

Interestingly, Hap2 of *CYP78A9* and *CAM7* was sensitive to environments. For example, the G2 and G3 accessions of *CYP78A9*-A04-Hap2 produced heavier seeds under long-day than under short-day conditions (Supplementary Fig. 29 and Supplementary Table 36). However, they showed delayed flowering under short-day environments and produced lighter seeds than the G4, G5 and G6 accessions of *CYP78A9*-A04-Hap3. The significant increase in TSW of G2 and G3 accessions under long-day environments is a major factor causing opposing phenotypes in accessions with these two haplotypes under long-day and short-day conditions. Quantitative PCR with reverse transcription (RT–qPCR) analysis showed that both *CYP78A9* and *CAM7* were upregulated in the large-seeded accession ‘7981’ (TSW, 2.65–4.30 g) compared to the small-sized seeds accession ‘SY’ (TSW, 1.40–2.46 g; Supplementary Fig. 30). Collectively, these results implicate *CYP78A9* and *CAM7* as causal genes for TSW in *B. juncea*. Haplotype analysis suggests that selection of these genes for local photoperiod adaptation induced diversification of seed size in *B. juncea*.

Meanwhile, we detected 30 genes significantly associated with TSW by both GWAS and selective-sweep scan (Supplementary Table 37). These genes included transcription factors, hormone signaling pathways, lipid transporters and ribosomal proteins, which require further investigations.

**Fig. 5:**
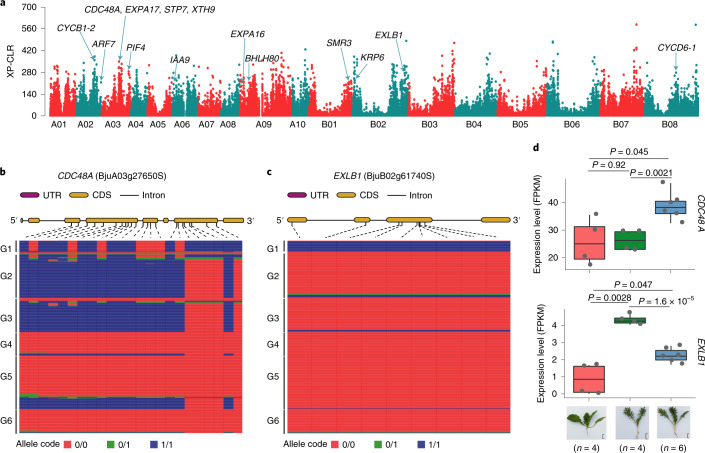
Identification of candidate genes for root enlargement in root mustard (*Brassica juncea ssp. napiformis*). **a**, Genome-wide distribution of selective sweeps related to tuber root formation in *B. juncea*. **b**, Haplotypes for the candidate gene *CDC48A* (BjuA03g27650S). **c**, Haplotypes for the candidate gene *EXLB1* (BjuB02g61740S). **d**, Expression levels of *CDC48A* and *EXLB1* in non-root and root mustard (before and 2 weeks after root enlargement) were estimated based on FPKM values. Box edges represent the 0.25 and 0.75 quartiles, with the median values shown by bold lines. Whiskers extend to data no more than 1.5 times the interquartile range, and remaining data are indicated by dots. *P* values were calculated using two-sided *t*-tests. Scale bars, 2 cm.

To investigate selection signatures putatively related to the domestication of root mustard, we compared the root mustard genomes to those of seed and leaf mustards using selective-sweep scan. In total, 2,803 sweep regions were identified in root mustard, covering 21.85 Mb with 5,756 genes (Supplementary Table 38). Fourteen candidate genes implicated in the formation of storage roots were identified (Fig. 5a and Supplementary Table 39), with putative functions in auxin signaling, sugar transport, cell division, cell expansion and cell wall modification. Of these, *CDC48A4* (BjuA03g27650S), participating in cell division and growth^69^, was found to have three haplotypes corresponding to the three independent domestication events (Fig. 5b). Its expression was upregulated during root enlargement in root mustard (Fig. 5d). The root and non-root mustards carried distinctly different haplotypes of the expansin gene *EXPB1* (BjuB02g61740S; Fig. 5c). Its expression was downregulated after root enlargement in root mustard (Fig. 5d), which is consistent with the expression patterns of *EXPB1* in *Raphanus sativus*^70^ and *Ipomoea batatas*^71^ during storage root development. We observed similar expression patterns in another expansin gene, *EXPA16* (BjuA09g18260S), and the cell elongation gene *XTH9* (BjuA03g32220S) after root enlargement in root mustard (Supplementary Table 39).

**Extended Data Fig. 6:**
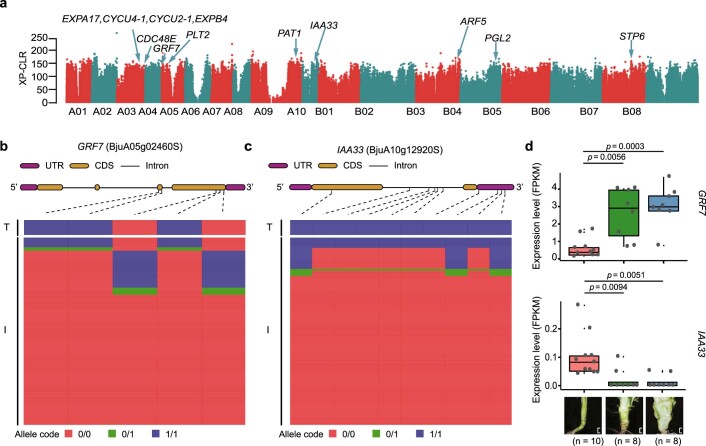
Identification of candidate genes for stem tuber formation in stem mustard. **a**, Genome-wide distribution of selective sweeps in stem mustard for stem tuber formation. **b**, Haplotypes for the candidate gene *GRF7* (BjuA05g02460S) in stem mustard (T) and leaf mustard (I). **c**, Haplotypes for the candidate gene *IAA33* (BjuA10g12920S) in stem mustard (T) and leaf mustard (I). **d**, The expression levels of *GRF7* and *IAA33* in non-stem mustard, stem mustard (one week after the stem swelled, three weeks after the stem swelled) (from left to right) were estimated based on FPKM values. In box plots, the center lines indicate the median values and the bottom and top edges of the box indicate the 25th and 75th percentiles, respectively. Whiskers extend to data no more than 1.5 times the interquartile range, and remaining data are indicated by dots. *p*-value was calculated using two-sided *t*-test. Scale bars, 2 cm.

Stem mustard is characterized by its enlarged edible stem with a diameter of > 20 cm, much bigger in diameter than leaf mustard (usually <5 cm^72^). We compared genomes of stem and leaf mustards and identified a total of 5,018 selective sweeps, spanning 46.51 Mb (Extended Data Fig. 6 and Supplementary Table 40). Twelve candidate genes selected during stem mustard breeding (Supplementary Table 41) are implicated in cell division, cell expansion, regulation of auxin signaling and glucose transport, functions with reported roles in storage organ formation in *Brassica*^73^. Expression of BjuA05g02460S, orthologous to *GRF7* (GROWTH-REGULATING FACTOR 7) regulating leaf and stem development^74^, was upregulated during stem swelling (Extended Data Fig. 6b,d), while the genes encoding auxin-responsive protein, *IAA33* (BjuA10g12920S), and the auxin-response factor, *MP* (also known as *ARF5*, (BjuB03g51870S), were downregulated after stem swelling (Extended Data Fig. 6c,d and Supplementary Table 41). This result contrasts with reports in turnip (*B. rapa* ssp. *rapa*)^75^, where expression of auxin-response genes did not change significantly during hypocotyl expansion. Overall, a greater subgenomic prevalence of selective sweeps related to root and stem swelling suggests that the A subgenome has undergone stronger selection than the B subgenome (Supplementary Tables 38 and 40). This finding is consistent with the high morphotype diversity of *B. rapa*^73^, which putatively provides a better selective substrate than the narrower range of variation present in *B. nigra*.

## Discussion

SY is a yellow-seeded landrace of *B. juncea* and represents a new form evolved from hybridization between two big gene pools. Therefore, SY is different from previously sequenced stem^19^ and Indian^25^ mustard. The chromosome-scale reference genome of SY, in conjunction with re-sequencing of 480 accessions, captured major genetic variation and allowed detailed reconstruction of the evolutionary and domestication history of this diverse ancient crop species. Plant genomics, together with archaeological evidence and historical written records, likely indicated a monophyletic origin of *B. juncea* in West Asia 8,000–14,000 years ago and at least three subsequent independent domestication events in the last 500–5,000 years: seed mustard near Central Asia, oilseed mustard in the Indian subcontinent and root mustard in East Asia. As *B. juncea* spread eastward, yellow-seeded (Oriental) mustard arose in Northwest China, stem mustard in the Sichuan Basin and probably broad-leaf mustard in eastern India, by selection acting on via spontaneous mutations. Hybridization of leaf mustard with yellow-seeded and root mustard gave rise to early-maturing yellow-seeded mustard in the Yunnan–Kweichow Plateau and lobed-leaf mustard (var. multisection Bailey) in eastern China, respectively. We also identified underlying genes and causal alleles for morphological variants such as root and stem swelling, flowering time and seed size variation associated with domestication and diversification. Our results not only elucidate the complex evolutionary and domestication history of *B. juncea*, but also pave the way for future research and breeding of this morphologically diverse condiment, oilseed, leaf, stem and root vegetable species.

## Methods

### Genome sequencing and assembly

#### Genome sequencing

High-molecular-weight DNA was isolated from fresh young leaves of *B. juncea* ssp*. juncea* var. SY. A SMRTbell library constructed with Sequel 1.0 reagents was sequenced on the PacBio Sequel. Illumina paired-end libraries of 350 bp in length were prepared following the manufacturer’s protocol. Hi-C libraries were performed as previously described^76^. Hi-C libraries were controlled for quality and sequenced on the Illumina HiSeq X Ten platform. Total RNA samples were extracted from root, stem, leaf, flower bud, siliques (7 and 15 d post-anthesis (DPA)), pod wall (20 DPA) and seed (20 DPA). RNA-sequencing (RNA-seq) libraries were made using the NEBNext Ultra RNA Library Prep Kit for Illumina (NEB) following the manufacturer’s recommendations and also sequenced on the Illumina X Ten platform.

#### Optical mapping

High-molecular-weight DNA extracted using the BioNano Plant Tissue DNA isolation kit (BioNano Genomics) was digested by Nt.BspQI and labeled with IrysPrep Labeling mix. The labeled DNA sample was loaded on the IrysChip and imaged using the BioNano Irys System.

#### Construction of a high-density *Brassica juncea* genetic map

A set of 172 recombinant inbred lines were derived from the cross SY × Purple Leaf Mustard (PM). Genomic DNA extracted from recombinant inbred line individual plants were digested with MseI. The fragments between 330 and 550 bp were gel excised and eluted. The pooled libraries were amplified and sequenced on a HiSeq 2000 platform. After stringent filtering, a total of 51,018 SNPs were identified in 21,210 genotyping-by-sequencing tags using the UNEAK pipeline^77^. To map the reads, the published *B. juncea* ‘T84-66’ genome (http://brassicadb.cn/#/SearchJBrowse/?Genome=Bju15/) was used as the reference. Genotyping of recombinant inbred lines was performed using a hidden Markov mode^78^ and the genetic map was constructed using MSTMap^79^.

#### De novo genome assembly

The genome size of SY was estimated by Jellyfish (v.2.2.9)^80^ using the *k*-mer of 17. After low-quality PacBio subreads shorter than 500 bp or with a quality score lower than 0.8 were filtered out, clean PacBio subreads were error-corrected and assembled into contigs by FALCON^23^ with the parameters --max_diff 100, --max_cov 100 and --min_cov 3, and then connected to scaffolds using Sspace-longread (v.1.1)^81^. After filling gaps using PacBio reads with PBJelly (v.1.9.1)^82^, gap-closed scaffolds were polished by Quiver^83^ and Pilon^84^ software with PacBio reads and Illumina data, respectively.

#### Scaffolding by integrating BioNano optical map

High-quality labeled molecules were pairwise aligned, clustered and assembled into contigs following the BioNano Genomics assembly pipeline. The BioNano Solve (V3.1) pipeline module ‘HybridScaffold’ was used to perform the hybrid assembly between the initial scaffold sequences and BioNano-assembled genome maps with the one-enzyme method. Using 202-fold coverage of BioNano data, we then generated an optical consensus map, which was implemented to assemble 1,897 super-scaffolds with an N50 of 5.87 Mb (assembly v2). Visualization of alignments between genome sequences and BioNano optical maps was performed by BioNano Access software (v1.5.1).

#### Pseudo-chromosomes assembly using the high-density genetic map

For pseudo-chromosomes assembly, markers of the high-density *B. juncea* genetic map were aligned to SY assembly V.2 by BWA (v. 0.7.8)^85^ mem. We set a threshold of at least three linked markers to order and orientate the contigs. Contigs which showed conflicts to the genetic map were called as potential mis-joins and checked based on marker continuity. A total of 35 mis-joins were found in 2,329 contigs and split to give 2,364 contigs after correction (Supplementary Table 2). Subsequently, the software Chromonomer (v.1.07, http://catchenlab.life.illinois.edu/chromonomer/manual/) was used to construct the initial pseudo-chromosomes of SY, with default parameters, following the internationally agreed nomenclature for *Brassica* chromosomes (http://www.brassica.info/resource/maps/lg-assignments.php).

#### Pseudo-chromosomes validation using Hi-C

To avoid artificial bias, the following type of reads were removed: (1) reads with ≥10% unidentified nucleotides (N); (2) reads with >10 bp aligned to the adaptor, allowing ≤10% mismatches; (3) reads with >50% bases having phred quality < 5. The filtered Hi-C reads were aligned to the initial pseudo-chromosome genome by BWA (v0.7.8)^85^ with default parameters. Reads were excluded from subsequent analysis if they did not align within 500 bp of a restriction site. Only uniquely mapped reads and valid paired-end ditags were used to validate the pseudo-chromosome sequences. The scaffolds of assembly V3 were used to make the Hi-C map by HiCPlotter^86^, and the interaction matrix of each chromosome was visualized with heat maps at the 25-kb resolution. A total of 165 mis-joined contigs were identified and manually broken using Juicebox^24^ according to the discrete chromatin interaction pattern. Of these, 150 mis-joined contigs, which lacked sufficient linked markers (three or more per contig or subcontig), were corrected and ordered by Hi-C contact map. Next, 13 mis-joins showing conflicts between the results of Hi-C data and the high-density map were broken, then re-clustered and ordered according to the Hi-C contact signal. Two remaining unanchored contigs that could not be anchored by the genetic map were repositioned to their pseudo-chromosome based on the Hi-C data.

#### Assessment of SY genome quality

The 1,440 conserved protein models in the BUSCO embryophyta_odb9 dataset (https://busco.ezlab.org/frame_wget.html) and the 248 conserved protein models in the CEGMA dataset (http://korflab.ucdavis.edu/dataseda/cegma/) were searched against the SY genome by using the BUSCO (v2)^87^ and the CEGMA (v. 2.5)^88^ programs with default parameters. Eighty-one BAC sequences and 2,567 BAC-end sequences from the PM BAC library were aligned to the SY genome by LASTZ^89^ with parameters (M = 254, K = 4,500, L = 3,000, Y = 15,000; --seed = match12 --step = 20 --identity = 85). LTRharvest^90^ (with parameters --similar 85.00 --vic 10 --seed 30 --seqids yes --motif TGCA --motifmis 1 --minlenltr 100 --maxlenltr 3,500 --mindistltr 1,000 --maxdistltr 20,000 --mintsd 4 --maxtsd 20) and LTR_FINDER^91^ (with parameters: --w 2 --l 100 --L 3,500 --d 1,000 --D 20,000 --M 0.3) were used to de novo predict the candidate LTR-RTs (full-length LTRs retrotransposon) in the SY assembly sequences. LTR_retriever^92^ was then used to combine and refactor all the candidates to get the final full-length LTR-RTs. LAI^27^ was calculated based on the formula: LAI = (intact LTR-RTs length/total LTR-RTs length) × 100%. As recommended by the steering group of the Multinational *Brassica* Genome Project (https://www.brassica.info/), the consistency of syntenic gene ordering was evaluated by exploiting the linkage mapping information depicted by the genome-ordered graphical genotypes^28^. Protein sequences of annotated HC genes from *B. juncea* vars. SY, T84-66 (ref. ^19^) and Varuna^25^, both progenitors *B. rapa*^29^ and *B. nigra*^30^, and previously reported *B. napus* cv. ZS11 (ref. ^31^) were reciprocal aligned using BLASTP with an *E*-value cutoff of 1e^−5^. The reciprocal best hit for each alignment was used to build whole-genome synteny between SY and the other five *Brassica* subgenomes by MCScanX^93^.

Detailed procedures for the SY genome annotation are provided in the Supplementary Note.

### Genome blocks and centromere detection

We first constructed the three subgenomes (LF, MF1 and MF2) following methods described previously^94^. Then, we defined the genomic blocks in SY based on the syntenic relationship of the *B. juncea* and *A. thaliana* genomes^95^. We aligned the A subgenome centromeric repeat sequences (CentBrs, CRB and TR805)^34,35^ and the B subgenome centromeric repeat sequences (CRB, pBNBH35 and CLs)^30,35–37^ to the SY assembly using BLAST (*E*-value 1e^−5^). The pericentromeric regions of A subgenome were detected using peri-centromere-specific retrotransposons and the tandem repeat sequence TR238 (ref. ^35^), whereas the pericentromeric regions of B subgenome contained more LTR*/gypsy* elements^30^. Then, the densities of centromeric repeat sequences were calculated to detect the centromere locations.

### Re-sequencing, reads mapping and SNP calling

A panel of 480 mustard accessions (Supplementary Table 18) were self-pollinated over multiple generations before re-sequencing. Genomic DNA extracted from fresh leaves was used for 350-bp Illumina libraries preparation. Sequencing protocols were the same as mentioned above. A total of 7.01 Tb (~14.48 Gb per sample) of clean data was generated after removing reads with ≥10% unidentified nucleotides (N), >10 nucleotides aligned to the adaptor or of which >50% bases had Phred quality scores less than 5. The paired-end reads were mapped to the SY genome using BWA (v0.7.8)^85^ with the command ‘mem --t 4 --k 32 --M’. Duplicated reads were removed with SAMtools (v.0.1.19)^96^. The genomic variants for each accession were then identified with the HaplotypeCaller module and the GVCF model by Genome Analysis Toolkit^97^ (GATK) software. All the GVCF files were merged. The high-quality SNPs and InDels were created in the HaplotypeCaller module filtered with the following four parameters: depth for individual ≥ 4, genotype quality for individual ≥ 5, minor allele frequency (MAF) ≥ 0.05, with missing rate ≤ 0.1 and heterozygous rate < 0.1. The identified SNPs and InDels were further annotated with ANNOVARtool (v2013-05-20)^98^, and divided into the following groups: variations occurring in intergenic regions, within 1 kb upstream (downstream) of transcription start (stop) sites, in coding sequences and in introns.

### Population structure and phylogenetic analyses

The population genetic structure was examined using the program ADMIXTURE (v1.23)^99^ with *K* values (the putative number of populations) from 2 to 10. The *K* = 6 value was chosen because clusters maximized the marginal likelihood. To better clarify the relationships of *B. juncea* accessions, 390 accessions with the genetic components of larger than 0.6 were retained for the further analysis. To construct maximum-likelihood phylogeny, we screened 30,609 synonymous SNPs to reduce influences of natural or artificial selection. Phylogenetic tree analysis was performed using IQ-TREE (v1.6.6)^100^, based on the best model (GTR + F + ASC + R7) determined by the Bayesian information criterion. Bootstrap support values were calculated using the ultrafast bootstrap approach (UFboot) with 1,000 replicates. Five known closely related species *A. thaliana*, *Crambe hispanica*, *Cardamine hirsuta*, *Eutrema halophilum* and *Eutrema* *salsugineum* were used as outgroups. The phylogenetic tree was visualized by the online tool EvolView (https://www.evolgenius.info//evolview/). PCAs were done by GCTA^101^. The population relatedness and migration events were inferred using TreeMix^102^. We ran the tree with the group 1 as the root group and made this the base tree topology. Then we ran TreeMix using introducing migration events from 1 to 6. To detect admixture, we computed *D*-statistics^103^ based on ABBA and BABA SNP frequency differences. For a triplet of taxa P1, P2 and P3, and an outgroup O, that follows the phylogeny of (((P1, P2), P3), O), a *D* statistic significantly different from zero indicates P3 exchanged gene with P1 (*D* value 0) or P2 (*D* value >0). Then, the *f-branch* statistic calculated introgressions among the six groups by the software package Dsuite^104^. The *f*_d_ statistic^105^ was used to calculate the fraction of introgression in G4 from G2 in 100-SNP windows, which signifies gene flow when 0 < *f*_d _< 1.

Nucleotide diversity (*π*) and fixation index (*F*_ST_) were calculated by vcftools^106^ and pairwise genetic distance was calculated by Arlequin (v.3.5.2.2)^107^. To estimate and compare the pattern of LD among different groups, the squared correlation coefficient (*r*^*2*^) between pairwise SNPs was computed using the PopLDdecay (v.3.40)^108^ software. Parameters in the program were --MaxDist 500 --MAF 0.05 --Miss 0.1. The average *r*^2^ value was calculated for pairwise markers in a 500-kb window and averaged across the whole genome.

To construct subgenome trees, we selected 390 *B. juncea* accessions with genetic components greater than 60% in each group and 68 *B. rapa* and 11 *B. nigra* samples (Supplementary Table 24). We selected 14,264 and 10,629 synonymous SNPs for the A and B subgenomes, respectively, filtered with the following processes: depth for individual ≥ 4, missing rate ≤ 0.1, MAF > 5%. The maximum-likelihood phylogeny for each subgenome was constructed using IQ-TREE (v1.6.6)^100^ based on the optimal models (TVM + F + ASC + R6) following the same pipeline implemented as that for the *B. juncea* phylogeny.

### Pairwise identity-by-descent detection

To investigate genome-wide introgression between G4 and G2, we identified haplotypes in the G4 accessions that were identical by descent (IBD) with individuals from both the original source of diversification, the G5 leaf mustard, and the source of introgression, the G2 yellow-seeded mustard, following an approach described previously^109^. To estimate the frequency of shared haplotypes along individual chromosomes, each chromosome was divided into bins of 10 kb with a sliding window of 5 kb, and the number of recorded IBD tracts between G4 and the two groups (G2 and G5) was computed per bin. As the total number of pairwise comparisons differed between the groups, these numbers were normalized from 0 (no IBD detected) to 1 (IBD shared by all individuals within the group). The normalized IBD between G4 and the G2 (nIBD_G2_) and the normalized IBD between G4 and the G5 (nIBD_G5_) were then used to calculate the rIBD (nIBD_G2 _− nIBD_G5_). Finally, the putative introgression segments from the G2 to each of the G4 accessions were identified.

### Estimation of divergence time and demographic history

With genome-scale characterization of the divergence of orthologous genes, we managed to date the divergence between *B. rapa* A genome and *B. juncea* A subgenome, between *B. nigra* B genome and *B. juncea* B subgenome, and between *Brassica* and *Arabidopsis*. The synonymous divergence (*K*_S_) values for *A. thaliana*, *B. rapa*, *B. nigra*, and A and B subgenomes of *B. juncea* were calculated using the *K*_A_/*K*_S_ Calculator (v2.0)^110^. The divergence time between species was calculated as *K*_S_/2 *µ*, where *µ* is the mutation rate (1.5 × 10^−8^ ~ 9 × 10^−^^9^ per synonymous site^111^).

SMC++ (v1.13)^112^ was used to estimate the divergence time and historical *Ne* among different groups of *B. juncea*. For normalizing the population size, we selected seven different samples from each group. Generations were calculated by the upper and lower mutation rates of 1.5 × 10^−^^8^ and 9 × 10^−^^9^ per synonymous site for each generation^111^, and the generation time was 1 year.

### Organellar genome analysis

The CP genomes were assembled by NOVOPlasty^113^ using genome re-sequencing data. After manually correcting the orientation of the two inverted repeats, the assembled CP genomes were annotated by GeSeq^114^. The InDel variants in CP genomes of *B. juncea* were identified through sequence alignment and confirmed by PCR (Extended Data Fig. 2a). The maximum-likelihood phylogeny of CP genomes was constructed based on high-quality variants (variants with >20% missing calls and MAF < 0.01) using RAxML (v8.0.17)^115^ with the GTRGAMMAI model. A bootstrap of 1,000 repetitions was used to assess the reliability of the phylogeny reconstructed. The MT genomes were assembled by Celera Assembler^116^ with default parameters using PacBio reads of ten *B. juncea* accessions. For the mitotype analysis, an InDel and a reported SNP locus^45^ were identified by sequence alignment and confirmed by PCR (Extended Data Fig. 2b). Phylogenetic tree analysis of MT genomes was performed through IQ-TREE (v1.6.6)^100^ using the best model (HKY + F) determined by the Bayesian information criterion with 1,000 bootstrap replicates.

### Measurement and statistical analysis of agronomic traits

The 390 *B. juncea* accessions were grown in four locations: Guiyang (Guizhou, E106.72/N026.58, short-day, mild-winter), Xiangtan (Hunan, E112.90/N027.86, short-day, mild-winter), Kunming (Yunnan, E102.72/N025.04, long-day, subtropical) and Urumqi (Xinjiang, E087.60/N043.80, long-day, continental steppe with large diurnal temperature differences) in 2018 (designated G18, X18, K18 and U18, respectively). The field trials were conducted with two replications. The flowering time was recorded as days to flowering by 25% plants. Open pollinated seeds were harvested and dried. The mean weight of a thousand seeds from the three replications was used for further analysis. Statistical analyses of phenotypic data were performed with the R packages Hmisc (v4.1.1)^117^ and Psych (v1.8.4)^118^.

### GWAS analysis

Only SNPs with MAF ≥ 0.05 and missing rate ≤ 0.1 in a population were used to carry out GWAS. This resulted in 4,423,439 SNPs that were used in GWAS for 390 *B. juncea* accessions. We performed GWAS using GEMMA (the genome-wide efficient mixed-model association) program^119^ under the mixed-linear model. The top three PCs were used for population-structure correction. The genetic relationship between individuals was modeled as a random effect using the kinship (K) matrix. Significant *P*-value thresholds (*P* < 10^−^^6^ and 10^−^^5^ for flowering time and TSW, respectively) were set to control the genome-wide type I error rate.

### Selective-sweep analysis

The XP-CLR score were calculated using the XP-CLR^120^ package with sliding windows of 10 kb that had a 5-kb overlap between adjacent windows. The top 5% regions were assigned to candidate selective regions, and genes in these regions were considered as candidate genes.

### Transcriptome analysis

Total RNA was isolated from a sampled organ with two biological replicates at a specific developmental stage to investigate expression of the genes associated with formation of special organs for enlarged roots and tuber stems. As above, RNA-seq libraries were constructed and sequenced on an Illumina X Ten. The clean reads were mapped against the SY genome using TopHat (v2.0.12)^121^ software. The number of reads mapped was counted using HTSeq (v0.6.1)^122^ and then FPKM values were calculated for each gene. Transcripts of less than one per million mapped reads were ignored. Analysis of differential gene expression between two samples was performed using the DESeq R package (v1.18.0)^123^. Genes with an adjusted *P* value < 0.05 found by DESeq were assigned as differentially expressed. Procedures for the RT–qPCR analysis are provided in the Supplementary Note.

### Reporting Summary

Further information on research design is available in the Nature Research Reporting Summary linked to this article.

## Online content

Any methods, additional references, Nature Research reporting summaries, source data, extended data, supplementary information, acknowledgements, peer review information; details of author contributions and competing interests; and statements of data and code availability are available at 10.1038/s41588-021-00922-y.

## Supplementary information

Supplementary Information Supplementary Note, Supplementary Figs. 1–30 and source data

Reporting Summary

Supplementary Tables 1–42

## Data Availability

The genome sequence and annotation data for *B. juncea* var. SY, the re-sequencing data for 480 *B. juncea* accessions and transcriptome data are accessible under NCBI BioProject no. PRJNA615316. For Functional annotation of the SY genome, the SwissProt (https://ftp.uniprot.org/pub/databases/uniprot/current_release/knowledgebase/complete/uniprot_sprot.fasta.gz/), NR (https://ftp.ncbi.nlm.nih.gov/blast/db/FASTA/nr.gz/) and KEGG (release 53; https://www.genome.jp/kegg/brite.html) databases were used. Seeds of accessions used, phenotype data and sequences of the CP and MT genomes reported here are available from the corresponding authors upon request. Source data are provided with this paper.

## Source data

Source Data Extended Data Fig. 2 Unprocessed gels.
