# Supplementary material for: Genomic insights into the origin, domestication and diversification of Brassica juncea
Source: Nat Genet. 2021 Sep 6;53(9):1392–402. doi: 10.1038/s41588-021-00922-y (PMC8423626; doi:10.1038/s41588-021-00922-y)
Supplement: Supplementary file 1 — Supplementary Note, Supplementary Figs. 1–30 and source data [file 41588_2021_922_MOESM1_ESM.pdf]

nature portfolio

https://doi.org/10.1038/s41588-021-00922-y

Supplementary information

Genomic insights into the origin, domestication and diversification of *Brassica juncea*

In the format provided by the authors and unedited

Genomic insights into the origin, domestication and diversification of *Brassica juncea*

Supplementary Notes

Methods

Genome annotation

The pipeline for prediction of repeat elements included *de novo* and homology-based approaches. For homolog evidence, alignment searches were undertaken against the RepBase database (http://www.girinst.org/repbase), and then were predicted by RepeatProteinMask (http://www.repeatmasker.org/). For *de novo* annotation, LTR_FINDER[1], PILER[2], RepeatScout (http://www.repeatmasker.org/), and Repeat-Modeler (http://www.repeatmasker.org/RepeatModeler.html) were used to construct a *de novo* library, then annotation was carried out with Repeatmasker (http://repeatmasker.org/).

A comprehensive strategy combing *ab initio* prediction, protein-based homology searches, and RNA sequencing was used to annotate the gene structure. Protein sequences from *B. juncea* (T84-66 V1.5), *B. napus* (Darmor V4.1), *B. rapa* (Chiifu V3.0), *B. nigra* (YZ12151), *B. oleracea* (HDEM), *Arabidopsis thaliana* were aligned to the SY genome using WUblast[3] with an *E*-value cutoff of 1*e*-5 and the hits were conjoined by Solar software[4]. GeneWise[5] was used to predict the exact gene structure

of the corresponding genomic regions for each WUblast hit. Gene structure created by GeneWise were denoted as Homo-set (homology-based prediction gene set). Gene models created by PASA[6] were denoted as the PASA Iso set, and the training data for the *ab initio* gene prediction programs. Five *ab initio* gene prediction programs Augustus (version 2.5.5)[7], Genscan (version 1.0)[8], Geneid[9], GlimmerHMM (version 3.0.1)[10] and SNAP[11] were used to predict coding regions in the repeat-masked genome. 29.6 Gb RNA-seq data (Supplementary Table 15) were mapped to the assembly using Tophat (version 2.0.8)[12], and Cufflinks (version 2.1.1)[13] and then used to assemble the transcripts into gene models (Cufflinks-set). In addition, gene models were predicted from Trinity-assembled transcripts by PASA[6], and was denoted as PASA-T-set (PASA Trinity set). Gene model evidence from the Homo-set, PASA-Iso-set, Cufflinks-set, PASA-T-set and *ab initio* programs were combined by EVidenceModeler (EVM)[14] into a non-redundant set of gene annotations. Weights for each type of evidence were set as follows: PASA-ISO-set > Homo-set > PASA-T-set > Cufflinks-set > Augustus > GeneID = SNAP = GlimmerHMM = Genscan. Gene models with low confidence scores were filtered out by the following criteria: (1) coding region lengths of 150 bp, (2) supported only by *ab initio* methods and with FPKM<1. We further classified these gene models to high-confidence (HC) and low-confidence (LC) protein-coding genes based on the following stringent confidence classification method. BLASTP was used to align the predicted peptide sequences to above-mentioned known protein datasets with an *E*-value cutoff of 1*e*-10. For each gene model, we selected the best-matching reference protein as a template sequence and defined the transcript sequence with

maximum coverage of the template as representative sequence. The high confidence (HC) genes were designated, if they had a significant BLAST hit to reference proteins and their representative protein had a similarity to the respective template sequence above a threshold to at least two references (>60% for *A. thaliana, B. oleracea*; >80% for *B. napus*, *B. rapa*, *B. nigra*; and >90% for *B. juncea*).

All protein-coding genes were aligned to two integrated protein sequence databases: SwissProt (https://ftp.uniprot.org/pub/databases/uniprot/current_release/knowledgeba-se/complete/uniprot_sprot.fasta.gz) and NR (ftp://ftp.ncbi.nlm.nih.gov/blast/db/FAS-TA/nr.gz). Protein domains were annotated by InterPro[15] and Pfam[16], respectively. The Gene Ontology (GO) terms for each gene were obtained from the corresponding InterPro and Pfam entry. The pathways in which the genes might be involved were assigned by BLAST against the KEGG databases (release 53, https://www.geno-me.jp/kegg/brite.html), with an *E*-value cutoff of 1*e*-5. Functional annotation results were merged from above two strategies.

The softwares tRNAscan-SE[17] and INFERNAL[18] were used to annotate genes for tRNA and for miRNA and snRNA, respectively. rRNA sequences were predicted by aligning to *Arabidopsis* template rRNA sequences.

qRT-PCR analysis

We selected twelve *B. juncea* accessions based on three haplotypes of *SRR1* and *VIN3*

associated with flowering time. These accessions were grown in the growth chamber under long-day (20°C with 65% relative humidity, a light intensity of 110 μmol photons $m^{-2}\cdot sec^{-1}$ and the photoperiod of 16 h light and 8 h dark for 20 days) or vernalization treatment (4°C with 65% relative humidity, a light intensity of 110 μmol photons $m^{-2}\cdot sec^{-1}$ and the photoperiod of 12 h light and 12 h dark for one month), and their relative expression of *SRR1* or *VIN3* was compared by qRT-PCR analysis. We used RNAs extracted from seeds 25 days post anthesis to compare relative expression of *CYP78A9* (BjuA04g00760S) and *CaM7* (BjuB05g28000S) between 'SY' (TSW 1.40~2.46g) and '7981' (TSW 2.65~4.30g) accessions by qRT-PCR analysis. The PCR program was run as follows: 95 °C for 3 min, followed by 39 cycles of 95 °C for 10 s, and 60 °C for 30 s. The PCR reaction volume was 20 μL in total, which included 2 μL diluted cDNA, 10 μl SYBR Premix Ex Taq II (Tli RNaseH Plus) (2×), 0.6 μL primers and 6.8 μL RNAfree water (TaKaRa, Tokyo, Japan). Three biological replicates and three technical replicates were conducted, and the relative expression levels were calculated using the $2^{-\Delta\Delta CT}$ method[19]. As an internal control, *HSP70-2* (BjuB04g43630S) was used to normalize the gene expression. The primers sequences are listed in Supplementary Table 42.

Restriction enzyme analysis

The restriction enzyme *Hinc*II (Thermo Scientific) was used to recognize the SNP at nt. 1799 of *VIN3* in *B. juncea* accessions. The PCR reactions contained 5μl 2X Fast LongTaq PCR PreMix (Innovagene, Changsha, China), 100ng DNA template and 0.2μl each primer (10umol/L), which was supplemented to 10μl with sterile water. The

following amplification program was used: denaturation at 94 °C for 3 min, 35 cycles of amplification for (94 °C/30 s, 56°C/30 s, 72 °C/30 s), and a final extension at 72°C for 5min. The PCR product (10 μl) was digested by adding 1μl *Hin*cII enzyme, 17μl H_2O, and 2μl buffer and incubated at 37 °C for 8 h. 8 μl of PCR product and 1μl of loading buffer were loaded into each sample well on a 1.5% agarose gel visualized by ethidium bromide stains under ultraviolet light.

References

1. Xu, Z. & Wang, H. LTR_FINDER: an efficient tool for the prediction of full-length LTR retrotransposons. *Nucleic Acids Res.* **35**, W265-268 (2007).
2. Edgar, R. C. & Myers, E. W. PILER: identification and classification of genomic repeats. *Bioinformatics* Suppl **1**, i152-i158 (2005).
3. She, R., Chu, J. S., Wang, K., Pei, J. & Chen, N. genBlastA: enabling BLAST to identify homologous gene sequences. *Genome Res.* **19**, 143-149 (2009).
4. Yu, X. J. et al. Detecting lineage-specific adaptive evolution of brain-expressed genes in human using rhesus macaque as outgroup. *Genomics* **88**, 745-751 (2006).
5. Birney, E., Clamp, M. & Durbin, R. GeneWise and Genomewise. *Genome Res.* **14**, 988-995 (2004).
6. Haas, B. J. et al. Improving the *Arabidopsis* genome annotation using maximal transcript alignment assemblies. *Nucleic Acids Res.* **31**, 5654-5666 (2003).
7. Stanke, M. et al. AUGUSTUS: *ab initio* prediction of alternative transcripts. *Nucleic Acids Res*. **34**, W435-W439 (2006).
8. Burge, C. & Karlin, S. Prediction of complete gene structures in human genomic

DNA. *J. Mol. Biol.* **268**, 78-94 (1997).

9. Guigo, R. Assembling genes from predicted exons in linear time with dynamic programming. *J. Comput. Biol.* **5**, 681-702 (1998).
10. Majoros, W. H., Pertea, M. & Salzberg, S. L. TigrScan and GlimmerHMM: two open source *ab initio* eukaryotic gene-finders. *Bioinformatics* **20**, 2878-2879 (2004).
11. Korf, I. Gene finding in novel genomes. *BMC Bioinformatics* **5**, 59 (2004).
12. Kim, D. et al. TopHat2: accurate alignment of transcriptomes in the presence of insertions, deletions and gene fusions. *Genome Biol.* **14**, R36 (2013).
13. Ghosh, S. & Chan, C.-K. K. in *Plant Bioinformatics* (ed. Edwards D.) **1374**, 339-361 (Humana Press, New York, 2016).
14. Haas, B. J. et al. Automated eukaryotic gene structure annotation using EVidenceModeler and the Program to assemble spliced alignments. *Genome Biol.* **9**, R7 (2008).
15. Hunter, S. et al. InterPro in 2011: new developments in the family and domain prediction database. *Nucleic Acids Res*. **40**, D306-D312 (2012).
16. Punta, M. et al. The Pfam protein families database. *Nucleic Acids Res.* **40**, D290-D301 (2012).
17. Lowe, T. M. & Eddy, S. R. tRNAscan-SE: a program for improved detection of transfer RNA genes in genomic sequence. *Nucleic Acids Res.* **25**, 955-964 (1997).
18. Nawrocki, E. P., Kolbe, D. L. & Eddy, S. R. Infernal 1.0: inference of RNA alignments. *Bioinformatics* **25**, 1335-1337 (2009).

19. Livak, K. J. & Schmittgen, T. D. Analysis of relative gene expression data using real-time quantitative PCR and the $2^{-\Delta\Delta CT}$ Method. *Methods* 25, 402-408 (2001).

Supplementary Figures

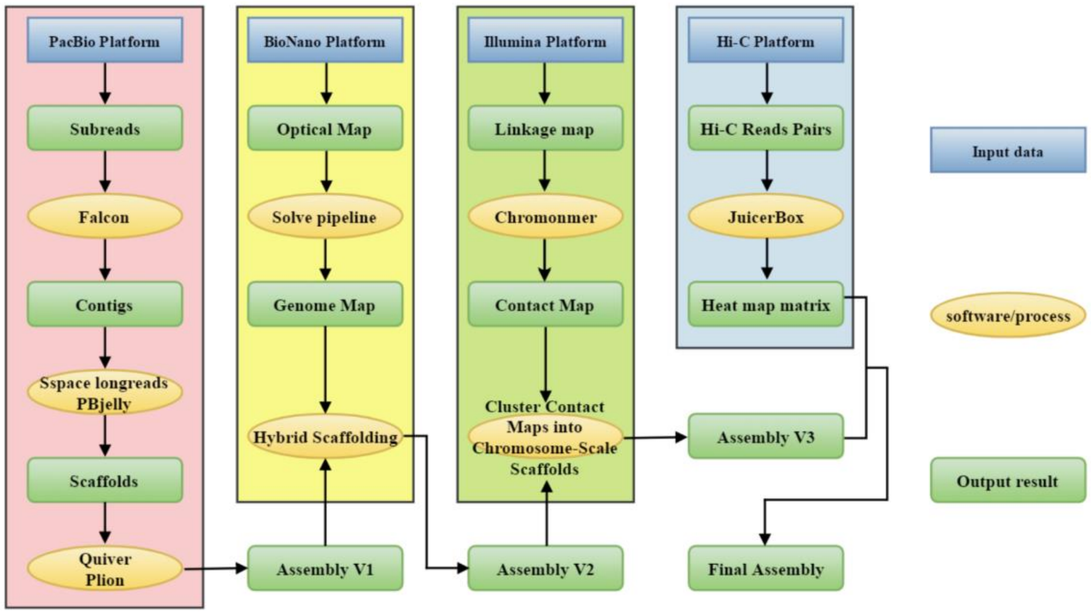

Supplementary Figure 1

The pipeline for *Brassica juncea* var. Sichuan Yellow genome assembly in this study.

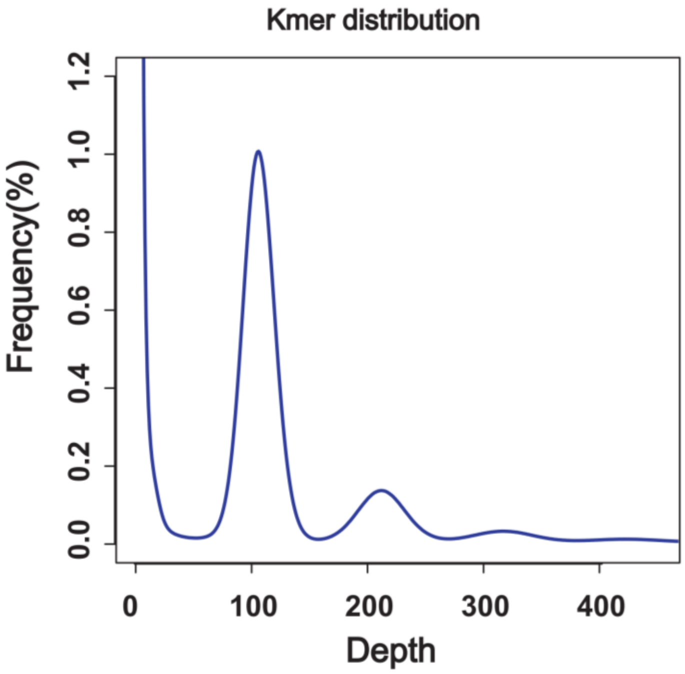

Supplementary Figure 2

Estimation of *Brassica juncea* var. Sichuan Yellow genome size by *K*-mer analysis. The figure shows the frequency of 17-mers, which are 17 bp sequences from clean reads of short-insert-size libraries. We identified 113,030,347,504 *K*-mers with a peak *K*-mer depth of 106. Genome size can be estimated as 1,066.32 Mb (total *K*-mer number/the volume peak). After revision, the genome size of *B. juncea* was estimated as 1,056.53 Mb.

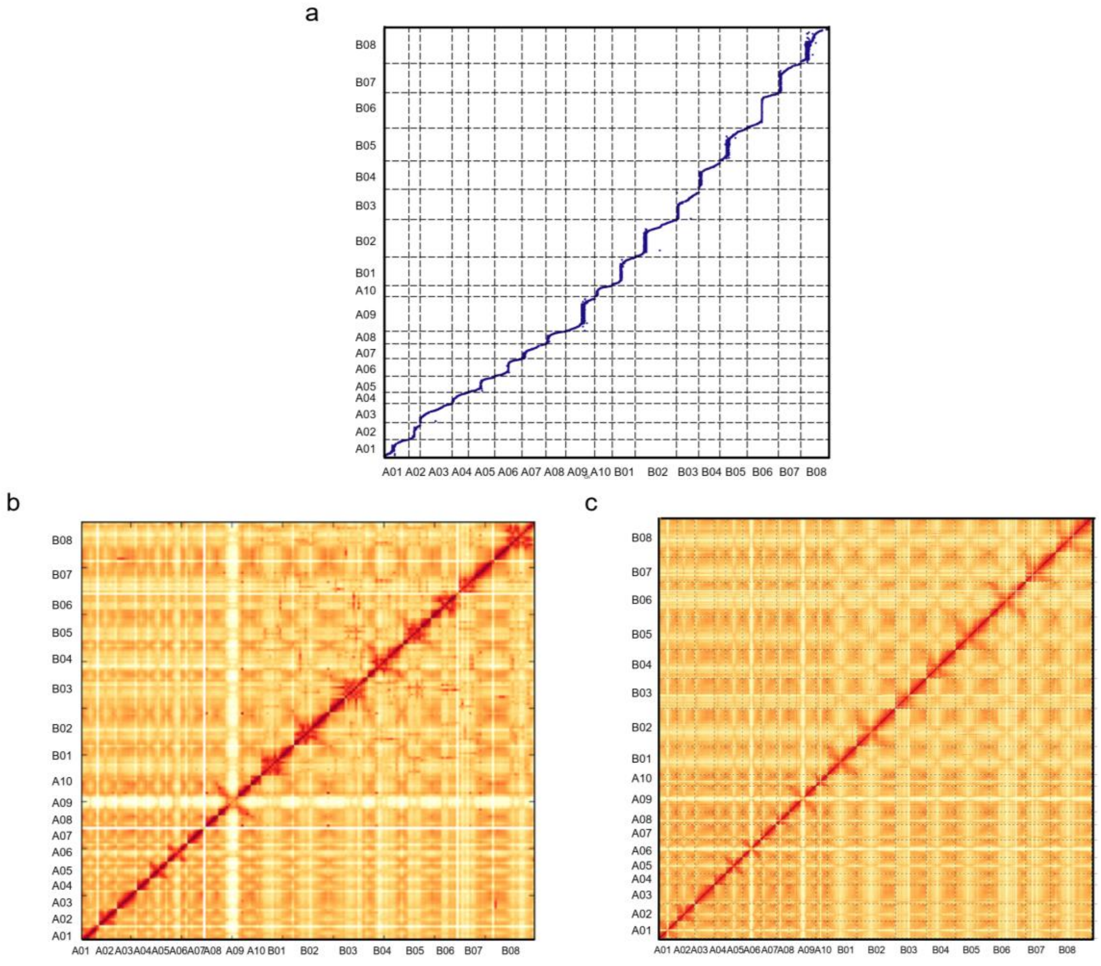

Supplementary Figure 3

The genetic map and Hi-C assisted assembly of *Brassica juncea* var. Sichuan Yellow pseudomolecules.

a, Comparison of SNP markers on the physical map (y-axis) with their position on the genetic map (x-axis). **b**, Heat map showing unnormalized contact matrix in 165 contig regions in V.3 assembly. **c**, Heatmap of chromatin interactions in each chromosome of SY.

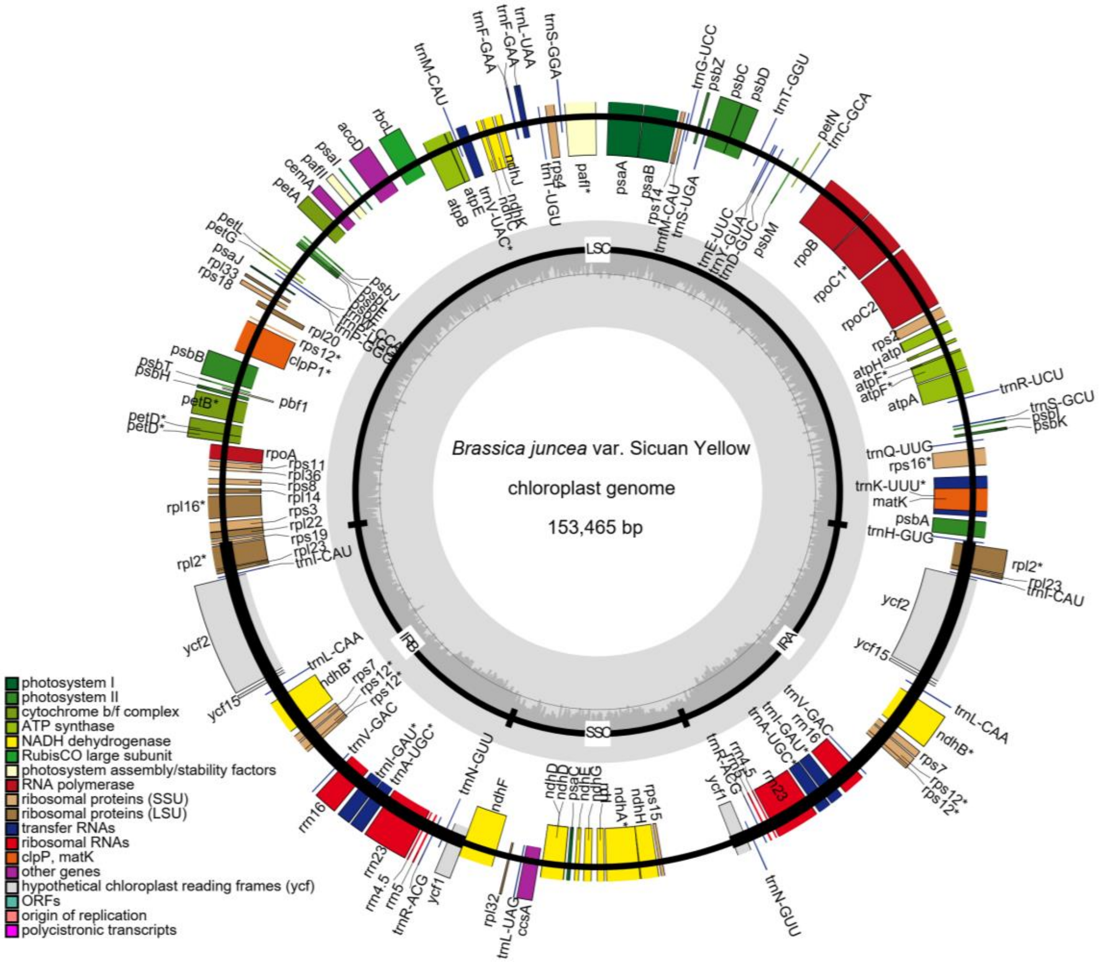

Supplementary Figure 4

Chloroplast genome map of *Brassica juncea* var. Sichuan Yellow.

Genes that belong to different functional groups are color-coded. GC content is represented on the inner circle by the dark gray plot.

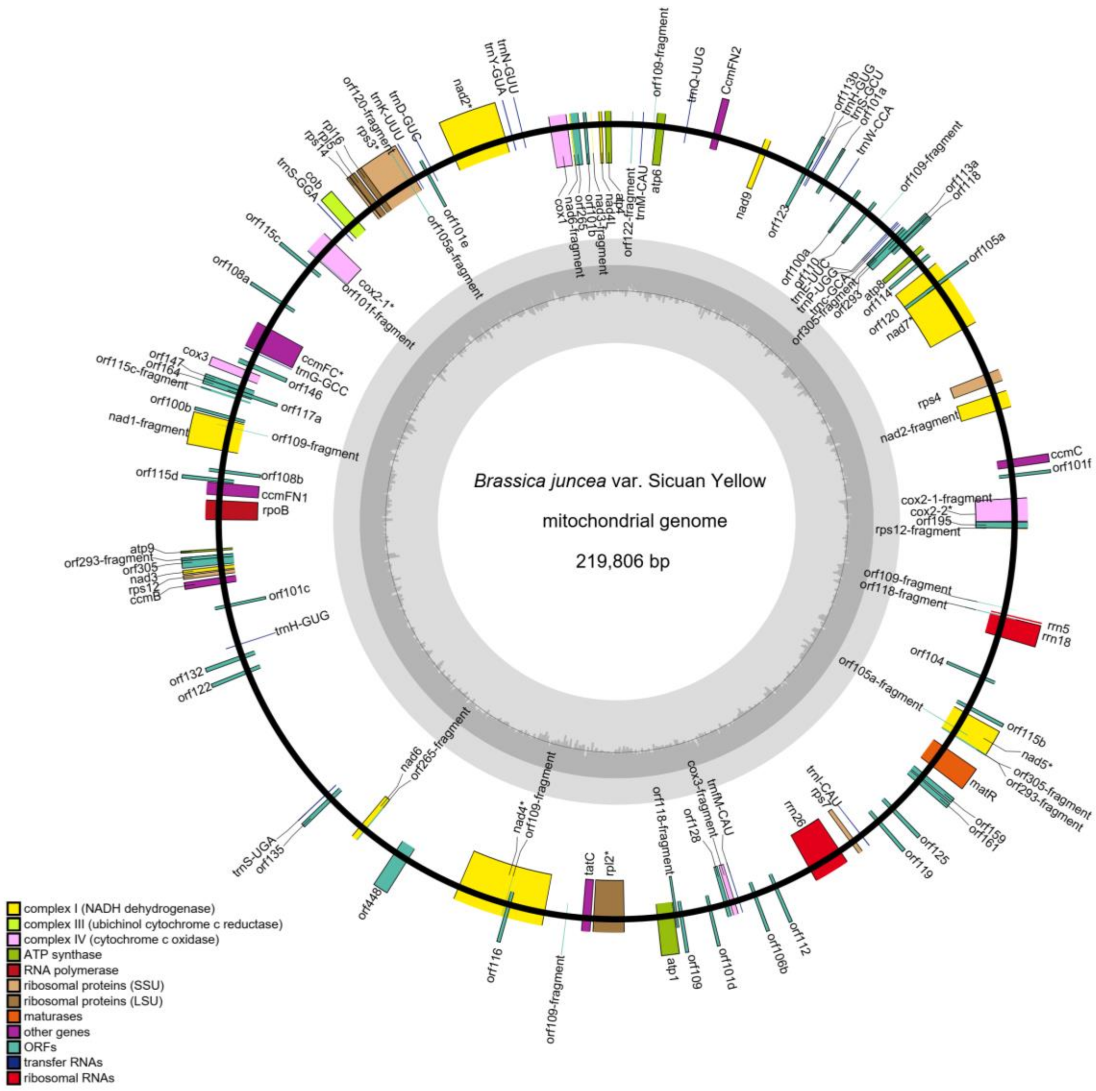

Supplementary Figure 5

Mitochondrial genome map of *Brassica juncea* var. Sichuan Yellow.

Genes that belong to different functional groups are color-coded.

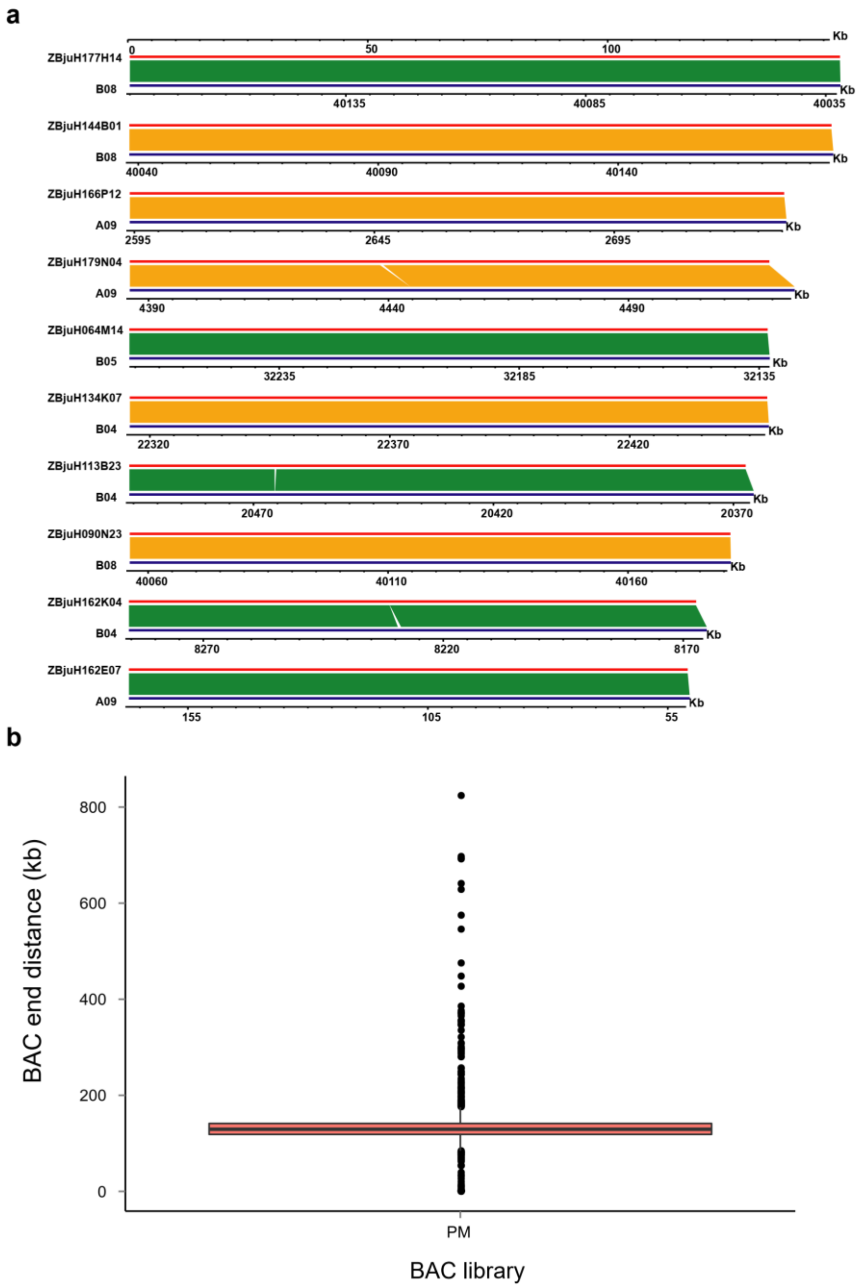

Supplementary Figure 6

Assessment of the *Brassica juncea* var. Sichuan Yellow genome assembly by selected BAC and paired BAC-end sequences.

a, Alignment of 10 BAC sequences to *B. juncea* SY chromosome, with forward and reverse orientations, shown in the graphic. The shaded regions between BAC sequences (upper red lines) and reference sequences of *B. juncea* (lower black lines) indicate the sequence alignments with forward (green) or reverse (orange) orientation. The white blocks indicate the unfilled gaps on the scaffolds. **b**, The distance of paired BAC-end sequences from the Purple Leaf Mustard (PM) BAC library.

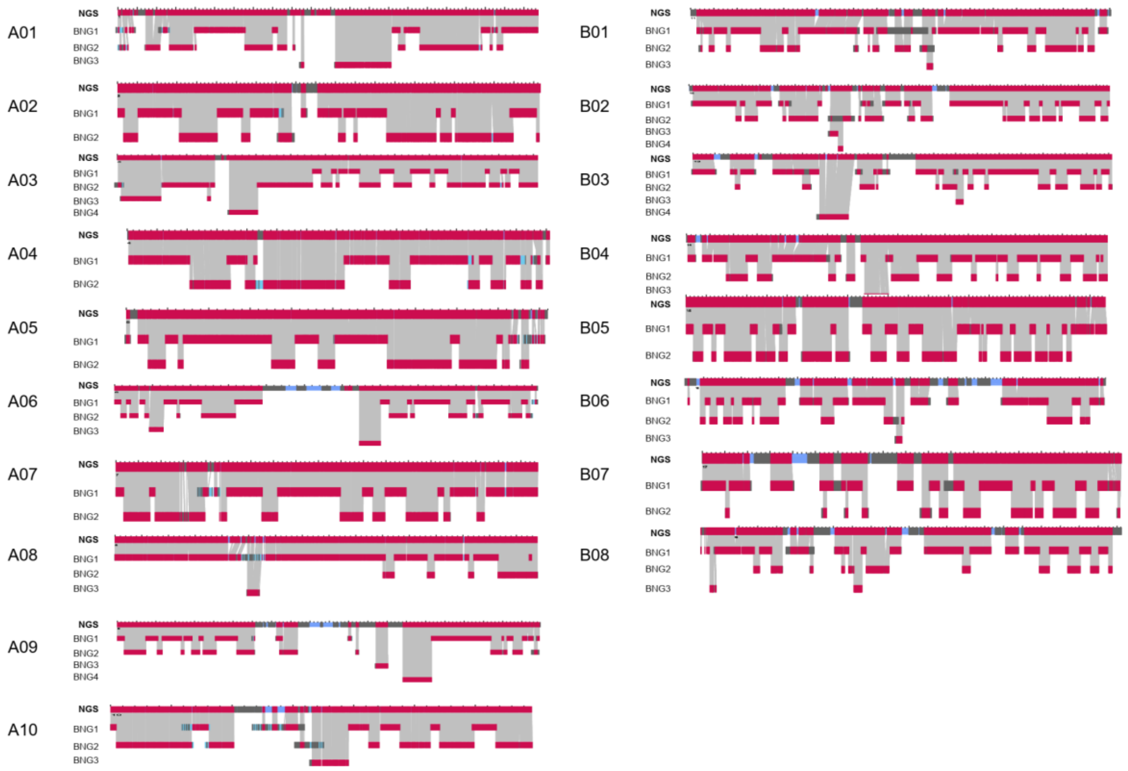

Supplementary Figure 7

BioNano alignment of *Brassica juncea* var. Sichuan Yellow genome.

NGS represents the SY assembly. BNG1-3 stand for the assembled scaffolds by optical consensus map. The gray lines represent the collinear regions. The alignment was performed with the optical map using Bionano Access V1.5.1.

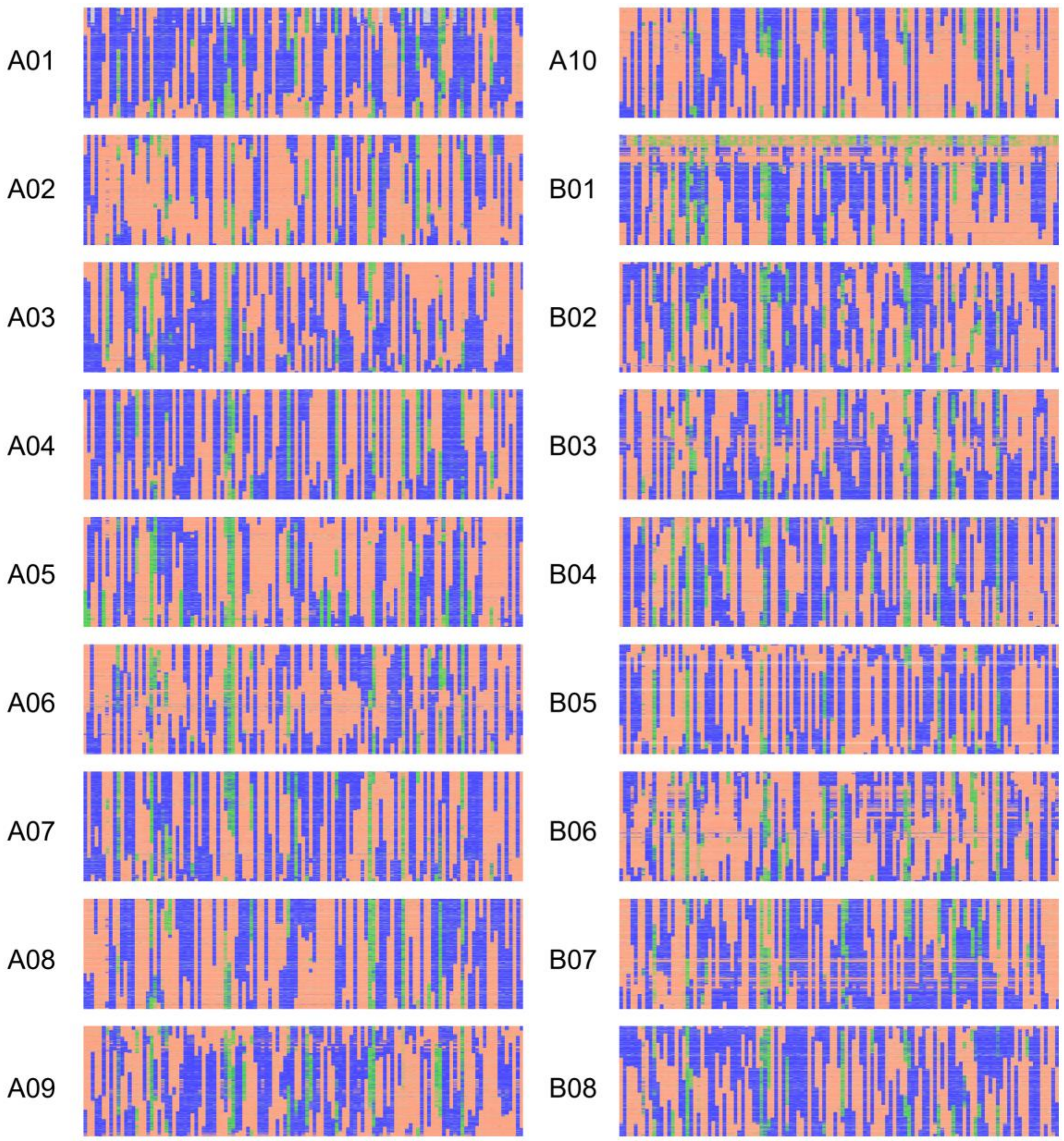

Supplementary Figure 8

Assessment of genome sequence organization of *Brassica juncea* var. Sichuan Yellow using genome-ordered graphical genotypes.

Graphical genotypes are shown for transcriptome SNP markers scored across 106 lines of the VHDH mapping population with Varuna alleles in pink, Heera alleles in blue, heterozygous calls in green, and missing scores in gray.

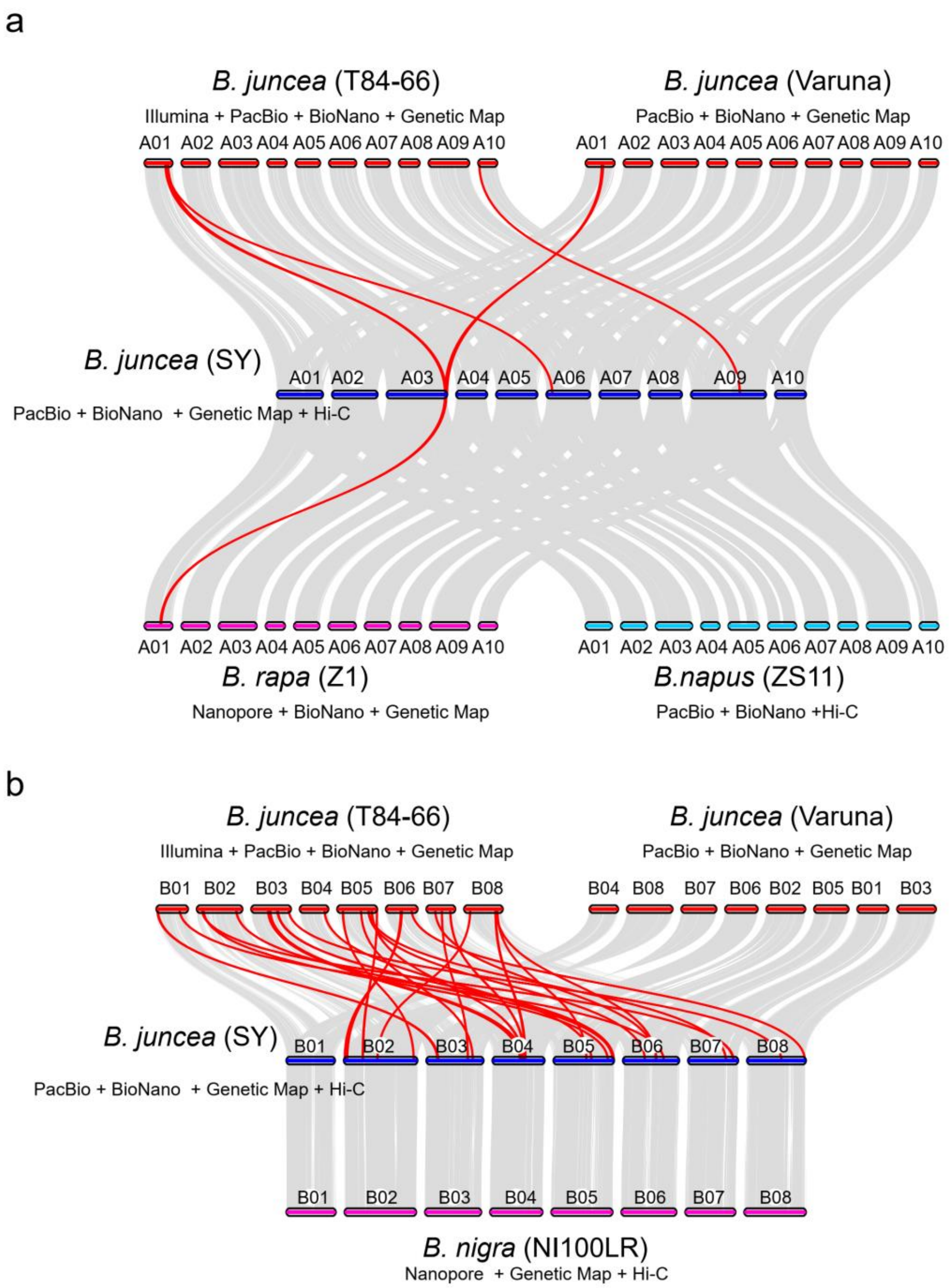

Supplementary Figure 9

Synteny among the subgenomes of *Brassica juncea* vars. SY, T84-66, Varuna, its diploid progenitors *B. rapa* and *B. nigra*, and previously reported *B. napus* cv. ZS11.

a, Synteny between *B. juncea* SY, T84-66, Varuna, *B. napus* ZS11 and *B. rapa* Z1 in the A genome. **b**, Synteny between *B. juncea* SY, T84-66, Varuna and *B. nigra* NI100 in the B genome. The red lines represent the syntenic blocks between SY and T84-66, Varuna or Z1. The gray lines connecting the chromosomes represent the syntenic blocks.

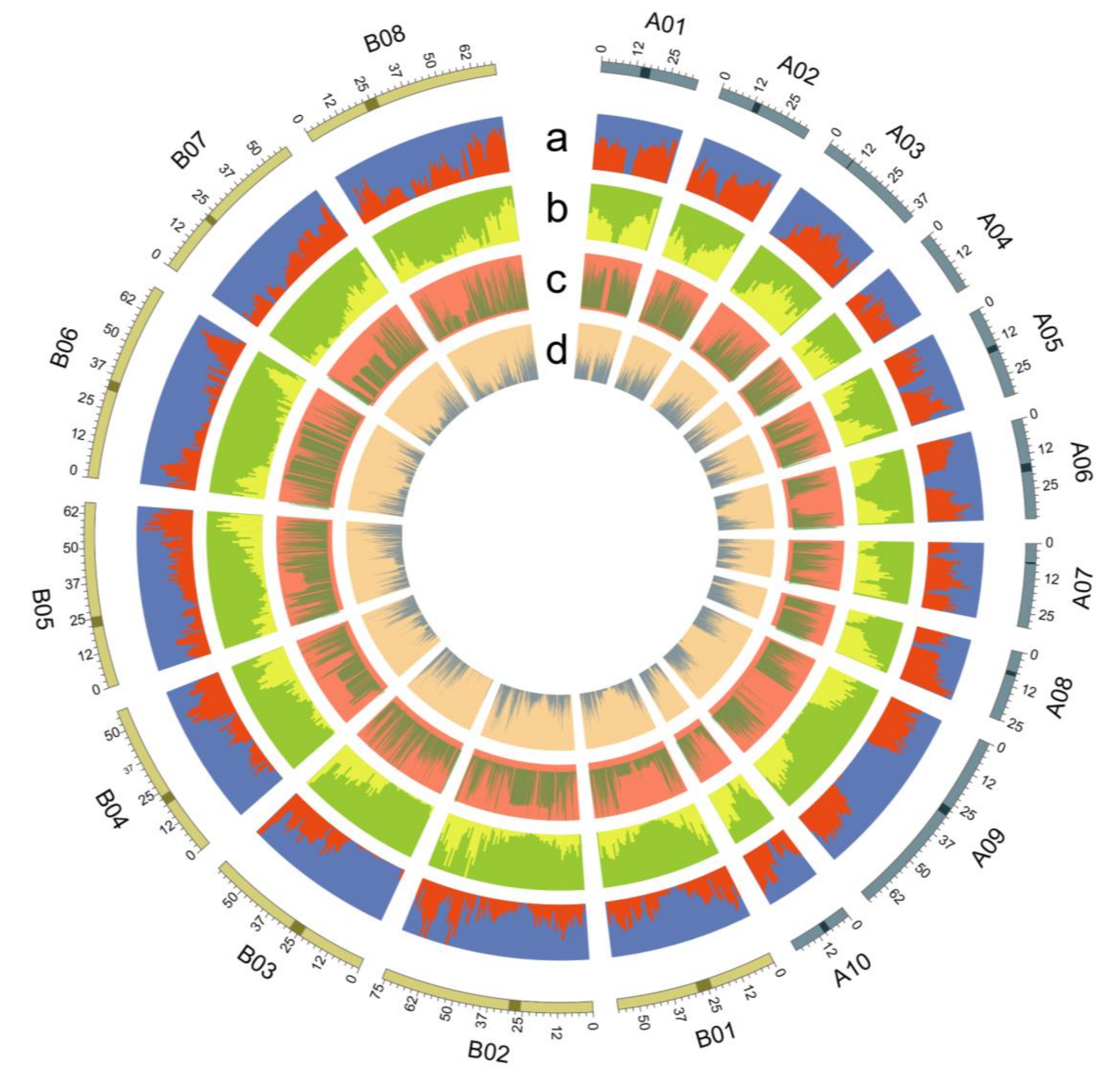

Supplementary Figure 10

Genetic diversity of *Brassica juncea* along the 18 chromosomes.

Tracks from outside to inside: **a**, SNP density; **b**, InDel density; **c**, Neutral evolutionary parameters (Tajima's D); **d**, Nucleotide diversity (π).

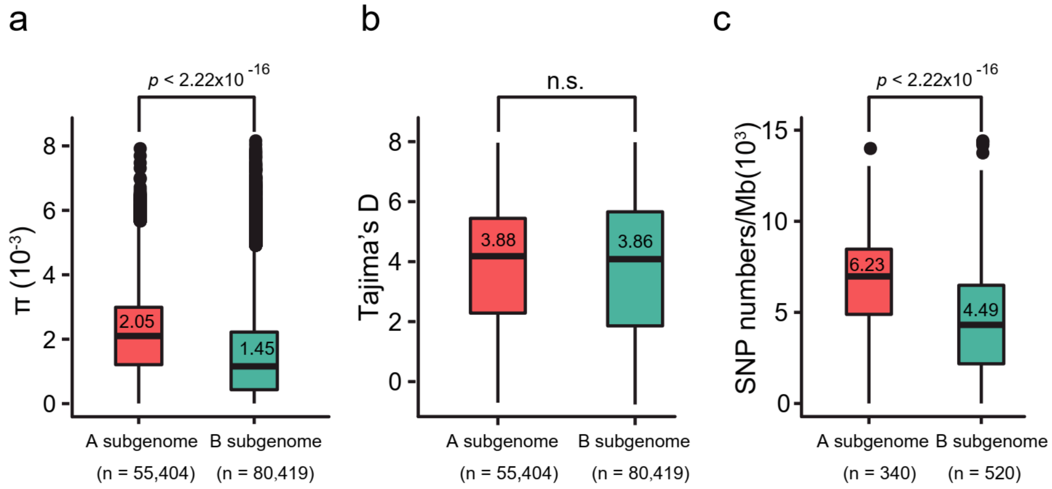

Supplementary Figure 11

Nucleotide diversity (*π*) (a), neutral evolutionary parameters (Tajima's D) (b) and SNP density (c) in the A and B subgenomes of *Brassica juncea*.

A sliding window analysis (1 Mb window, 5 kb steps) was used to calculated the *π* and Tajima's D. The box represents the interquartile range. The center line in each box indicates the median, and the number inside each box represents the mean value. The box limits indicate the upper and lower quartiles of divergence. *p*-value was calculated using two-sided *t*-test. n.s., not statistically significant.

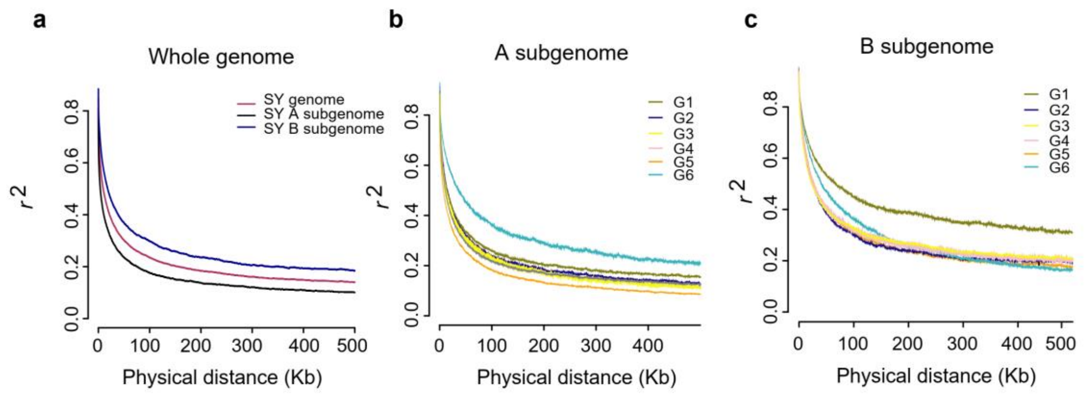

Supplementary Figure 12

Decay of linkage disequilibrium (LD) of whole genome (a), A (b), and B (c) subgenome of *Brassica juncea*.

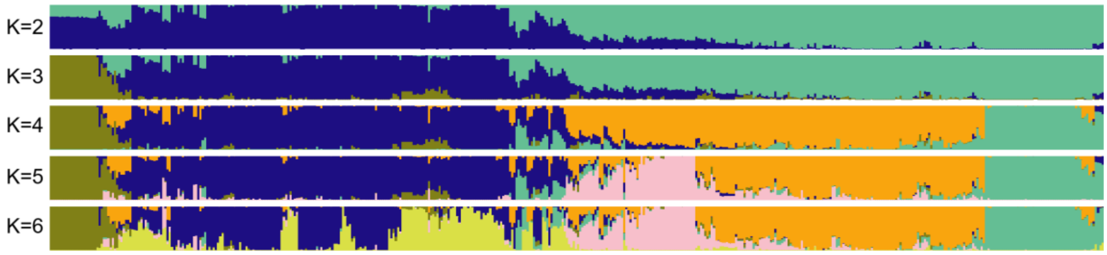

Supplementary Figure 13

Genetic structure of 480 *Brassica juncea* accessions.

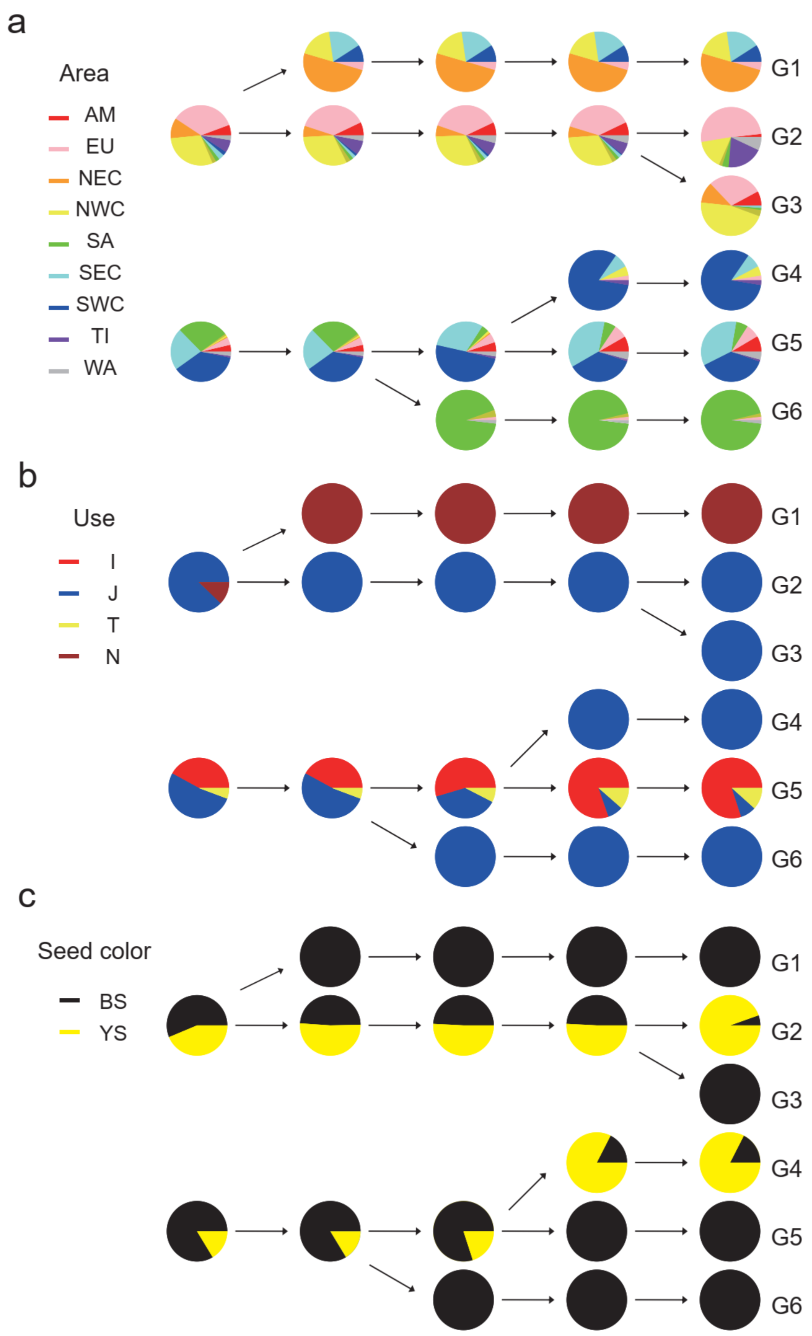

Supplementary Figure 14

The distribution of geographic origins (a), uses (b), and seed color (c) in different subpopulations of *Brassica juncea* based on different numbers of clusters (*K* = 2-6).

a, AM, America. EU, Europe. NEC, North China, Northeast China and Korea. NWC, Northwest China. SA, South Asia. SEC, East China, South China and Japan. SWC, Southwest China (except Tibet). Ti, Tibet. WA, West Asia. **b,** I, ssp. *integrifolia*. J, ssp. *juncea*. T, ssp. *tumida*. N, ssp. *napiformis*. **c**, BS, brown or black seed. YS, yellow seed.

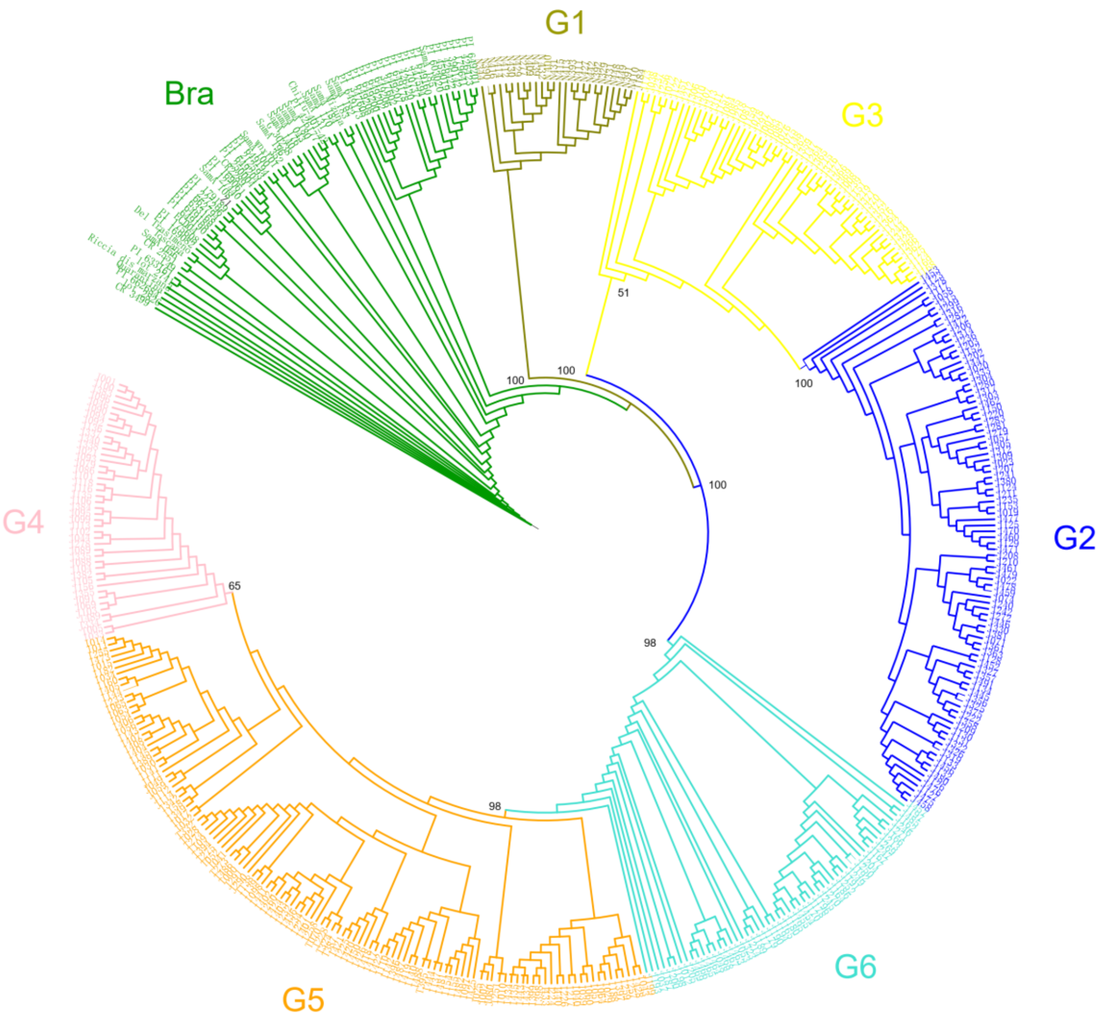

Supplementary Figure 15

Maximum-likelihood phylogenetic tree of the A subgenomes of *Brassica juncea* and *B. rapa*.

Bootstrap support values of key nodes are labelled on the tree. Bra, *B. rapa*.

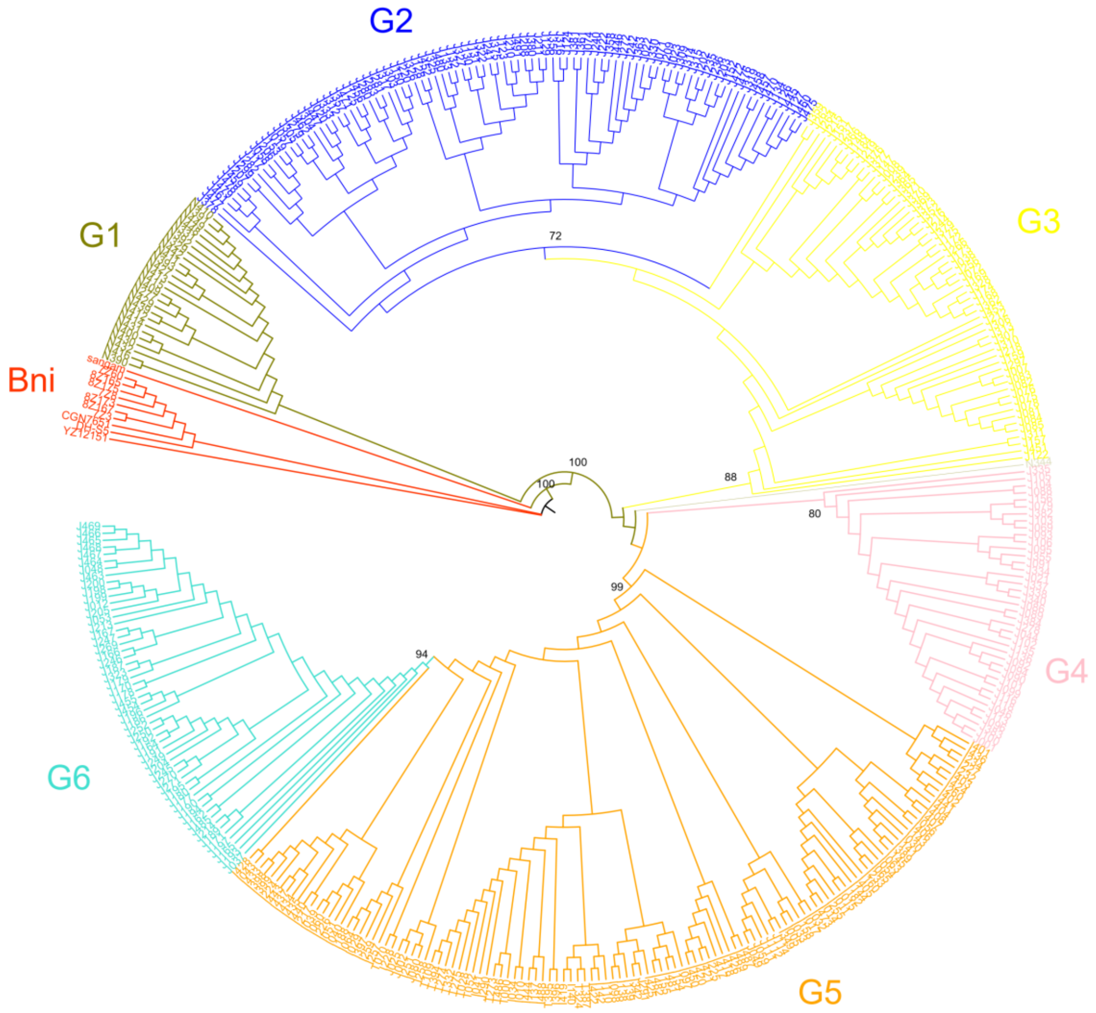

Supplementary Figure 16

Maximum-likelihood phylogenetic tree of the B subgenomes of *Brassica juncea* and *B. nigra*.

Bootstrap support values of key nodes are labelled on the tree. Bni, *B. nigra*.

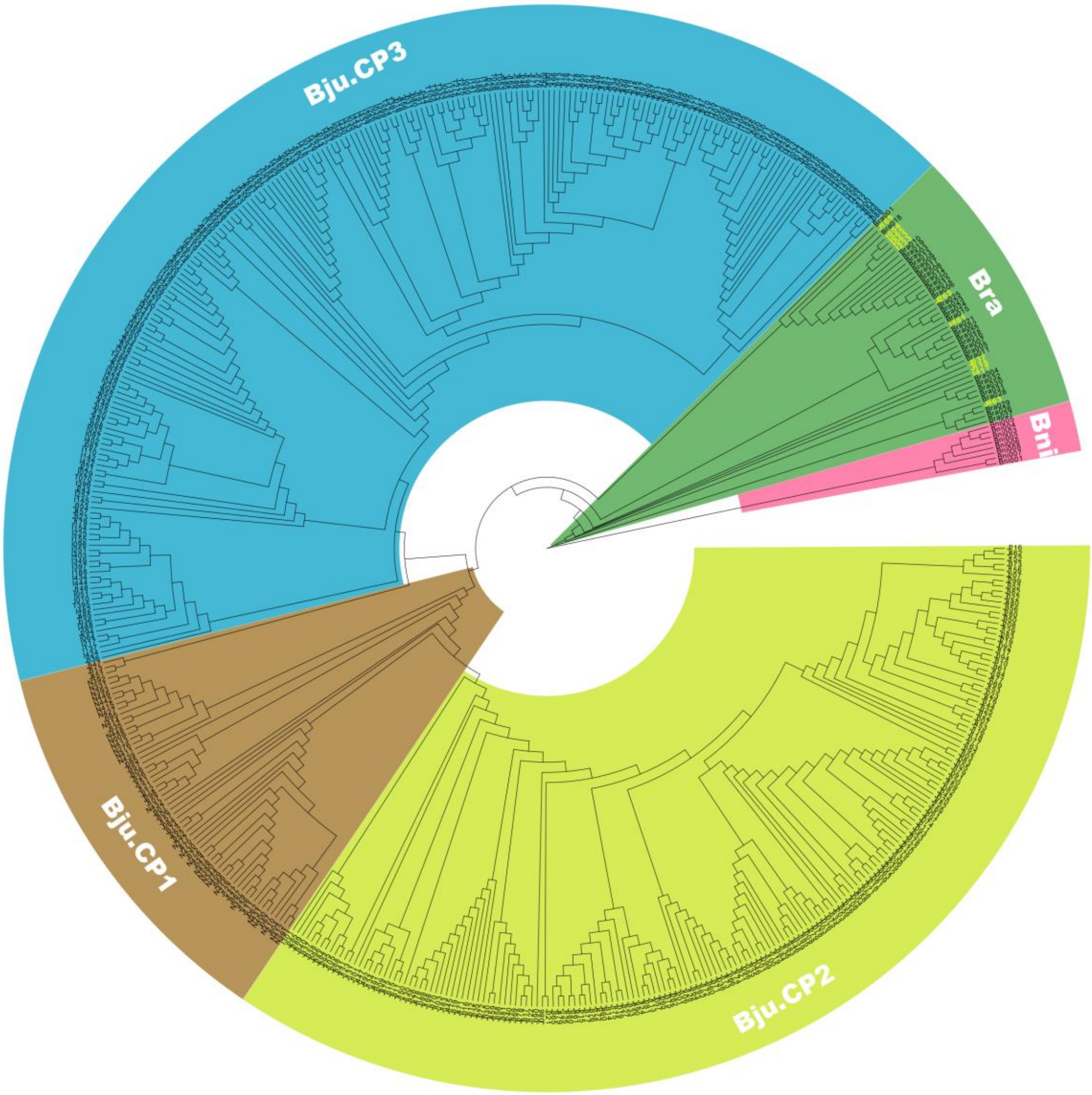

Supplementary Figure 17

Maximum-likelihood phylogeny of chloroplast genomes of *Brassica juncea* and its progenitor species.

The three *B. juncea* chloroplast genome types (Bju.CPs1-3) were identified by two InDels (Extended Data Fig. 2a). Eleven mustard accessions (in yellow) were clustered in the *B. rapa* clade, listed in Supplementary Table 18. Bra, *B. rapa*; Bni, *B. nigra*.

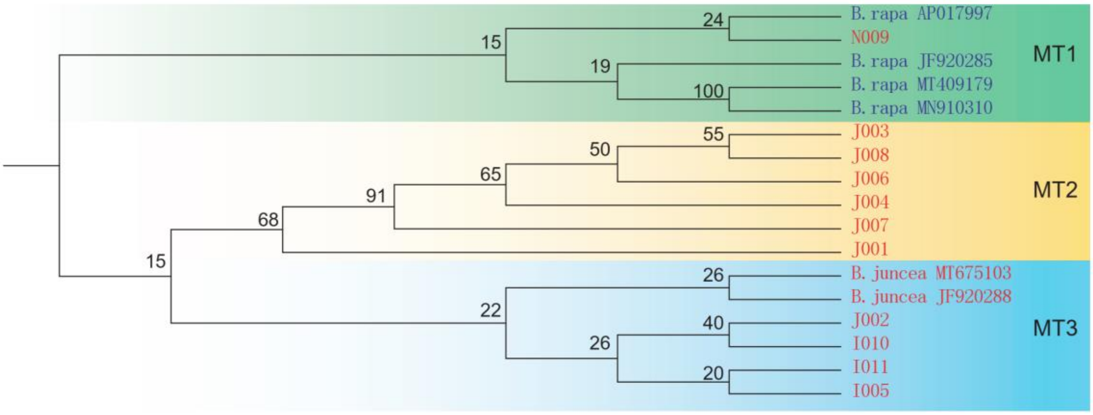

Supplementary Figure 18

Maximum-likelihood phylogeny of mitochondrial genomes of *Brassica juncea* and its progenitor species *B. rapa*.

The three *B. juncea* mitochondrial genome types (MT1-3) were identified by InDel and SNP variants (Extended Data Fig. 2b). Four *B. rapa* mitochondrial genomes were classified as MT1 based on the same InDel and SNP variants in *B. juncea* (for GenBank ID see Supplementary Table 25b).

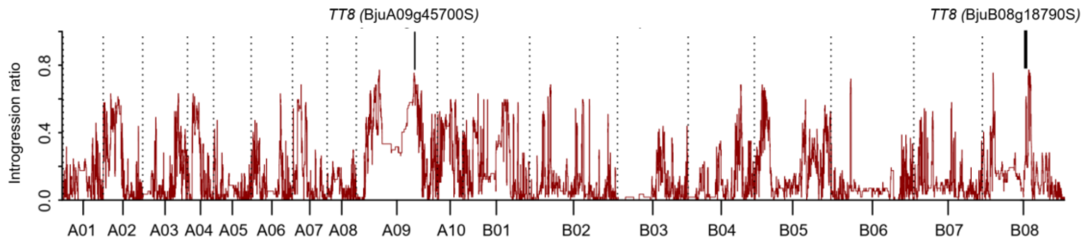

Supplementary Figure 19

Putative origins of introgression from G2 detected in G4 as illustrated by relative identity by descent (rIBD).

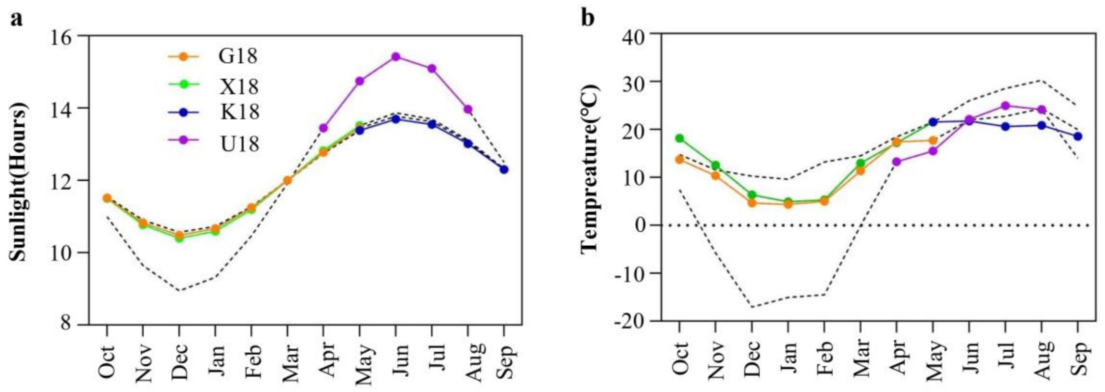

Supplementary Figure 20

Climate data for four different environments tested in China.

a and b, The graphs show sunlight and monthly mean temperature in four different environments from October 2017 to September 2018, respectively. The environments are abbreviated as follows: Guiyang, from Oct., 2017 to May, 2018 (G18); Xiangtan, from Nov., 2017 to May, 2018 (X18); Kunming, from May to Aug. in 2018 (K18), and Urumqi, from Apr. to Aug. in 2018 (U18).

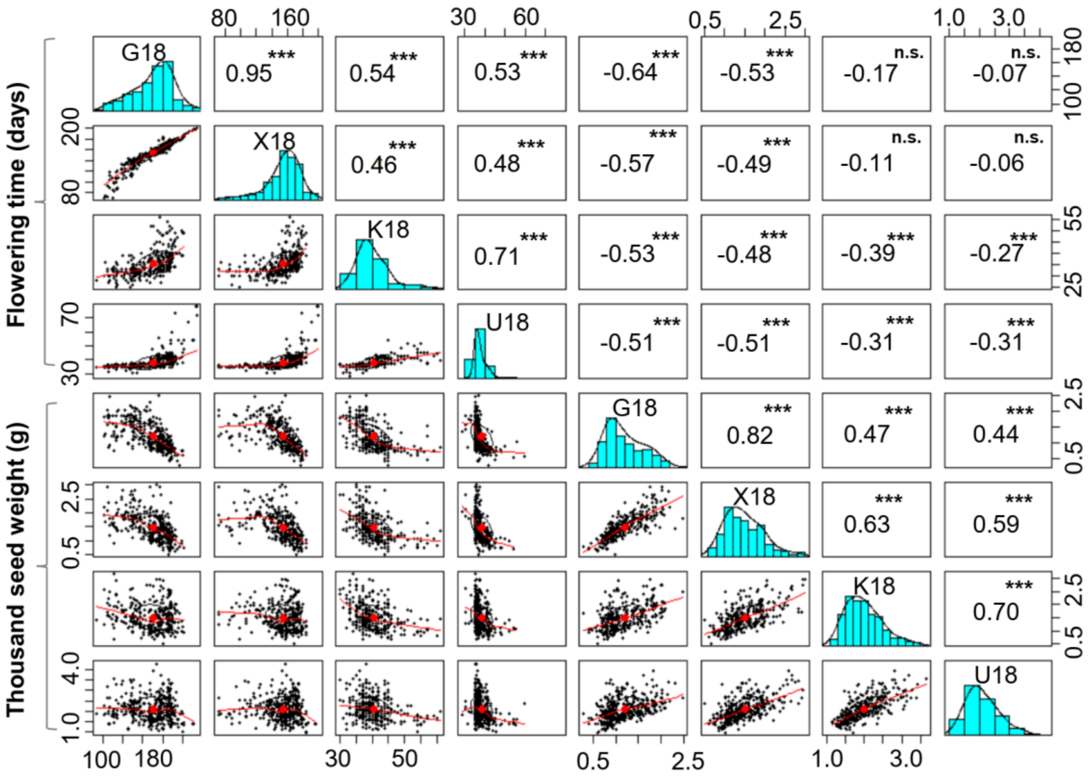

Supplementary Figure 21

Correlation coefficients and frequency distributions for flowering time and thousand seed weight in 390 *Brassica juncea* accessions.

****P* < 0.001; n.s., not significant (Pearson correlation coefficient)

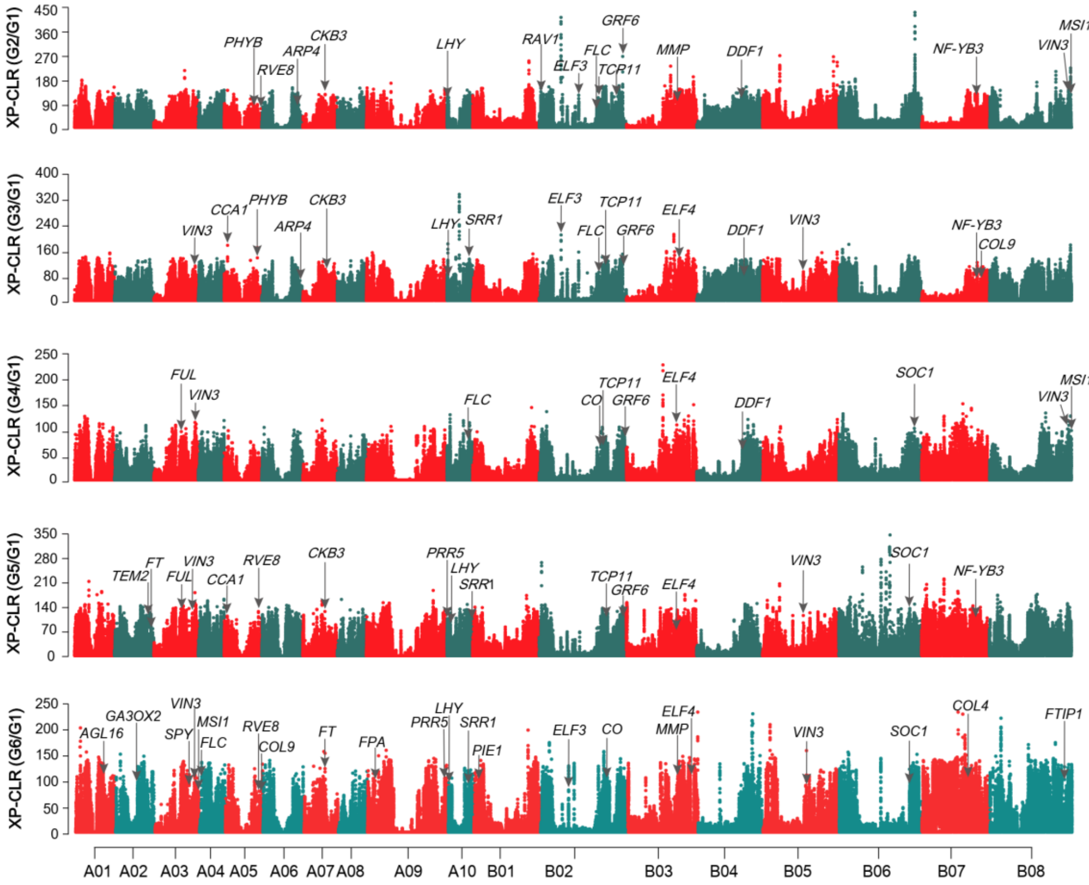

Supplementary Figure 22

Genome-wide screening of selective signals for flowering time between the group G1 and G2, G3, G4, G5 or G6 in *Brassica juncea* by XP-CLR analysis.

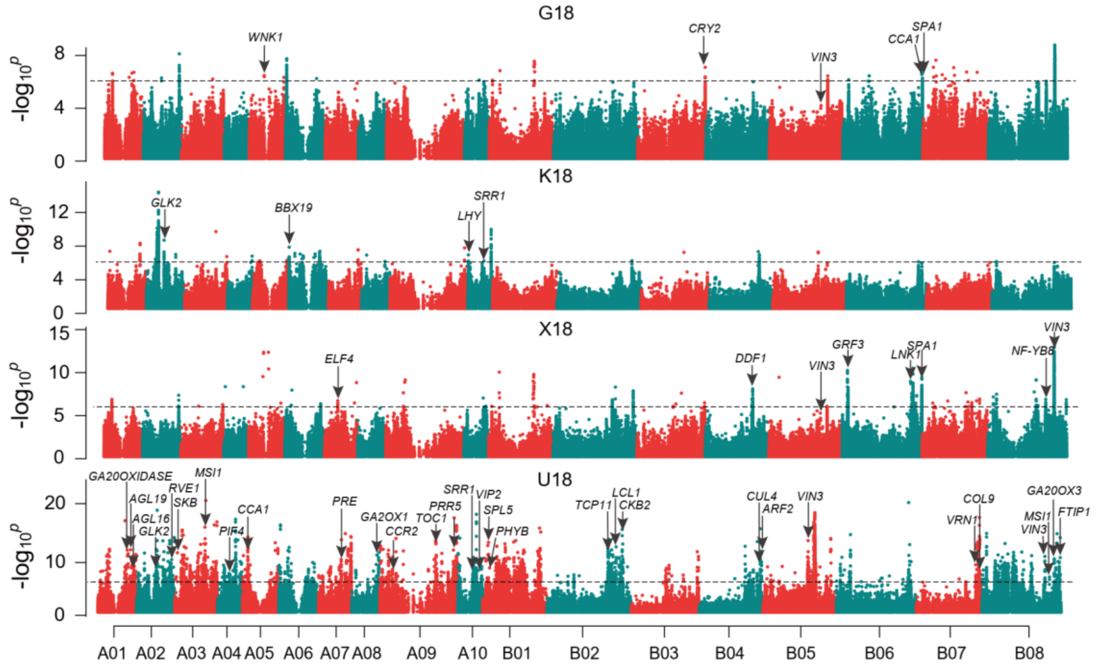

Supplementary Figure 23

GWAS of flowering time in *Brassica juncea* under four environments.

Candidate genes significantly associated with flowering time are labeled under each environment. The significance threshold of -$\log_{10}P$ value was set at 6.

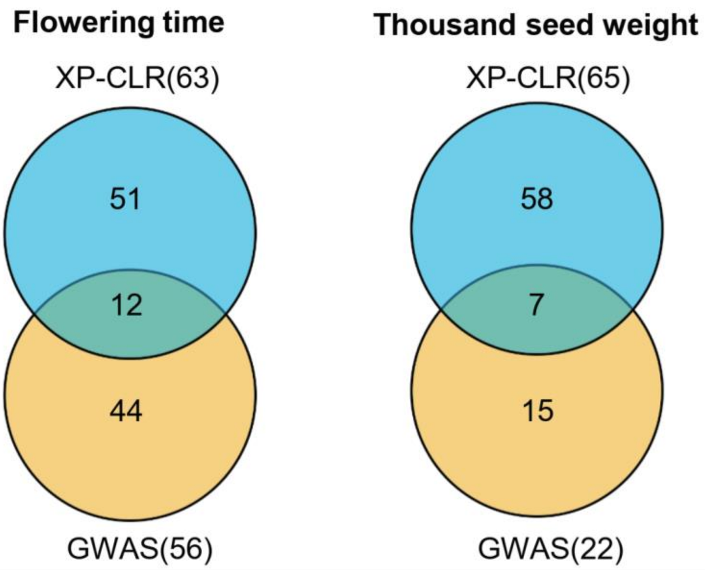

Supplementary Figure 24

Venn diagram for the number of candidate genes for flowering time (left) and thousand seed weight (right) in *Brassica juncea* from selective sweeps and GWAS.

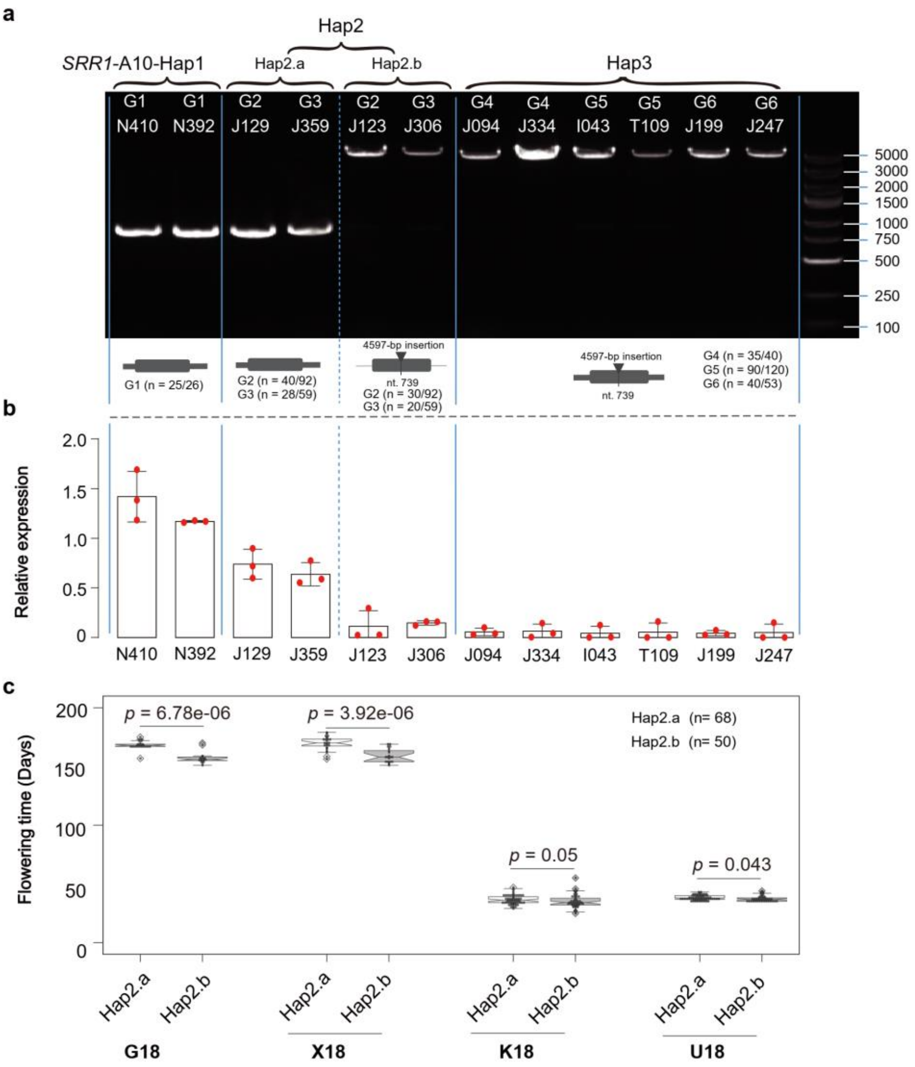

Supplementary Figure 25

Structural variation and expression analysis of the *Brassica juncea SRR1* (BjuA10g14550S).

a, PCR amplification detected a 4597 bp insertion at nt. 442 in *SRR1*-A10-Hap2.b and *SRR1*-A10-Hap3 accessions. Source data of gel was provided as a Supplementary Data 1. **b**, Relative expression level of different *SRR1* haplotypes by qRT-PCR. Reference gene: *HSP70-2*; $n = 3$. Error bars, s.d. (n = 3). **c**. Comparison in flowering time between *SRR1*-A10-Hap2.a (n = 68) and -Hap2.b (n = 50) accessions. Box edges represent the 0.25 and 0.75 quantiles with the median values shown by bold lines. Whiskers extend to data no more than 1.5 times the interquartile range, and remaining data are indicated by dots. *p*-value was calculated with two-sided *t*-test. For details of accessions see Supplementary Table 18.

a

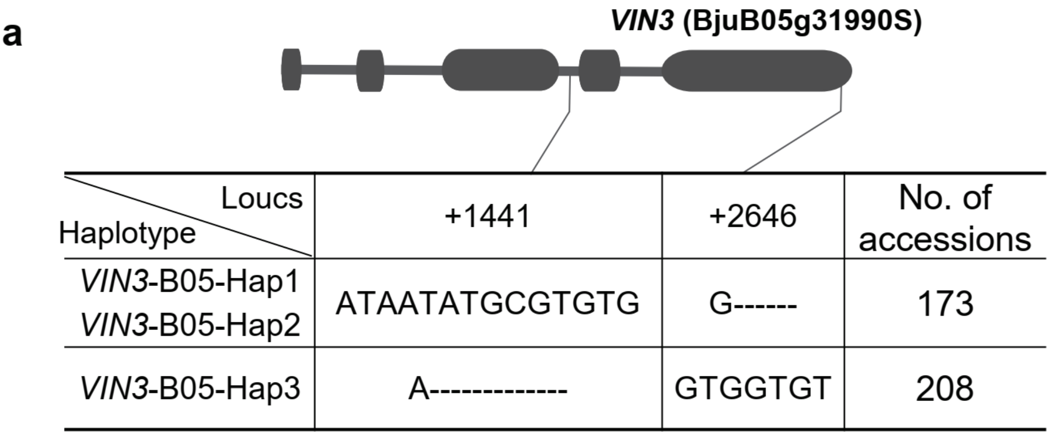

Loucs / Haplotype	+1441	+2646	No. of accessions
VIN3-B05-Hap1<br>*VIN3*-B05-Hap2	ATAATATGCGTGTG	G------	173
VIN3-B05-Hap3	A-------------	GTGGTGT	208

b

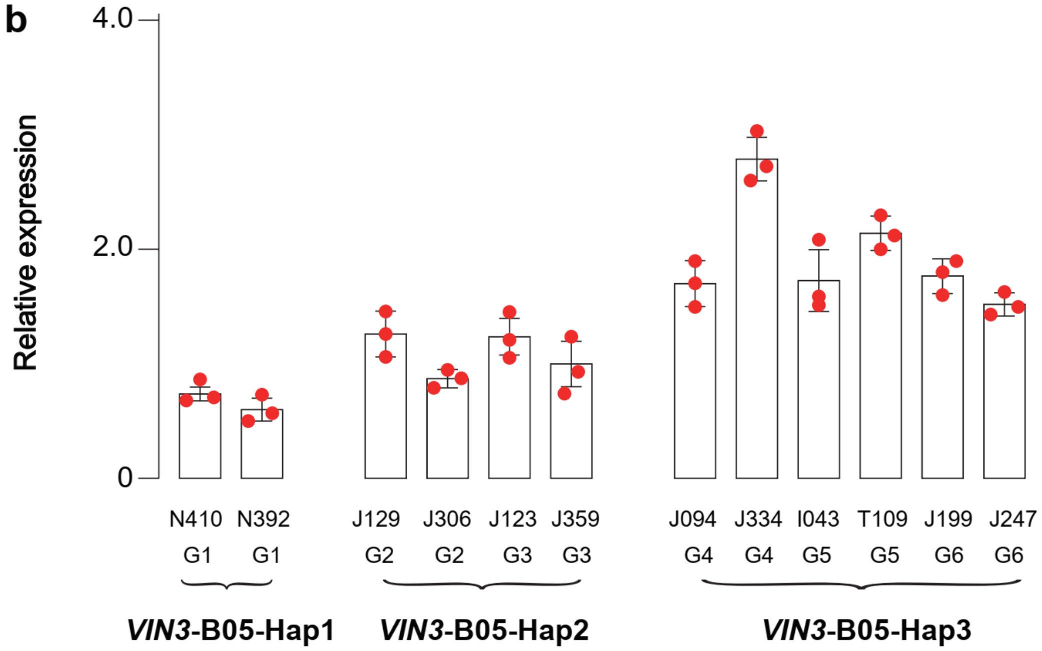

Supplementary Figure 26

Structural variations and expression analysis of the *Brassica juncea VIN3* (BjuB05g31990S).

a, A diagram illustrating two InDels detected by PCR amplification of the gene *VIN3*.

b, Relative expression level of different *VIN3* haplotypes by qRT-PCR. Reference gene: *HSP70-2*; $n = 3$. Error bars, s.d. (n = 3). For details of accessions see Supplementary Table 18.

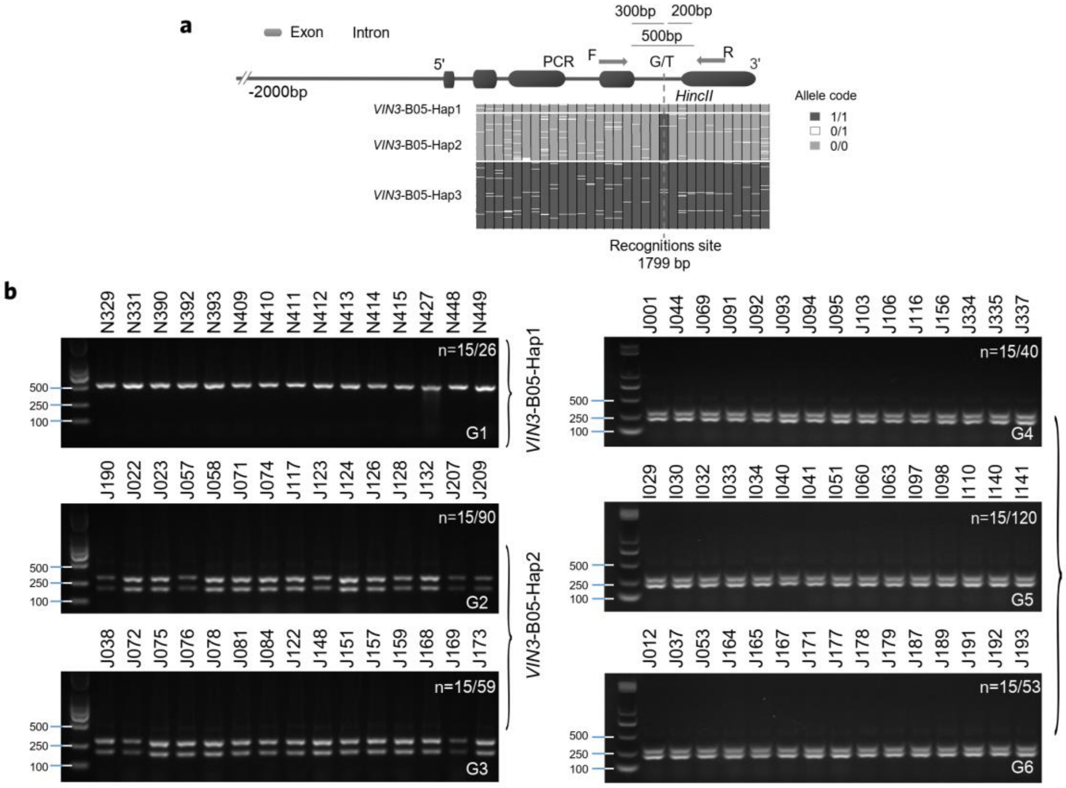

Supplementary Figure 27

Electrophoretograms of *Brassica juncea VIN3* (BjuB05g31990S) amplified fragment digested by *Hin*cll among accessions of three haplotypes.

a, The target region of *VIN3* amplified by PCR. **b**, The *VIN3* fragment amplified from 90 randomly selected *B. juncea* accessions were digested by the restriction enzyme *Hin*cII, which recognized the DNA sequence of Hap2 and Hap3 in the SNP site. The primer sequences used for PCR: *VIN3*-F, ACCGTGTTTCTTTAGGACAGA; *VIN3*-R, GGTTCAAGTGCTTGGATGGTA. The PCR experiments were repeated independently for three times with similar results. Source data of gels were provided as a Supplementary Data 2.

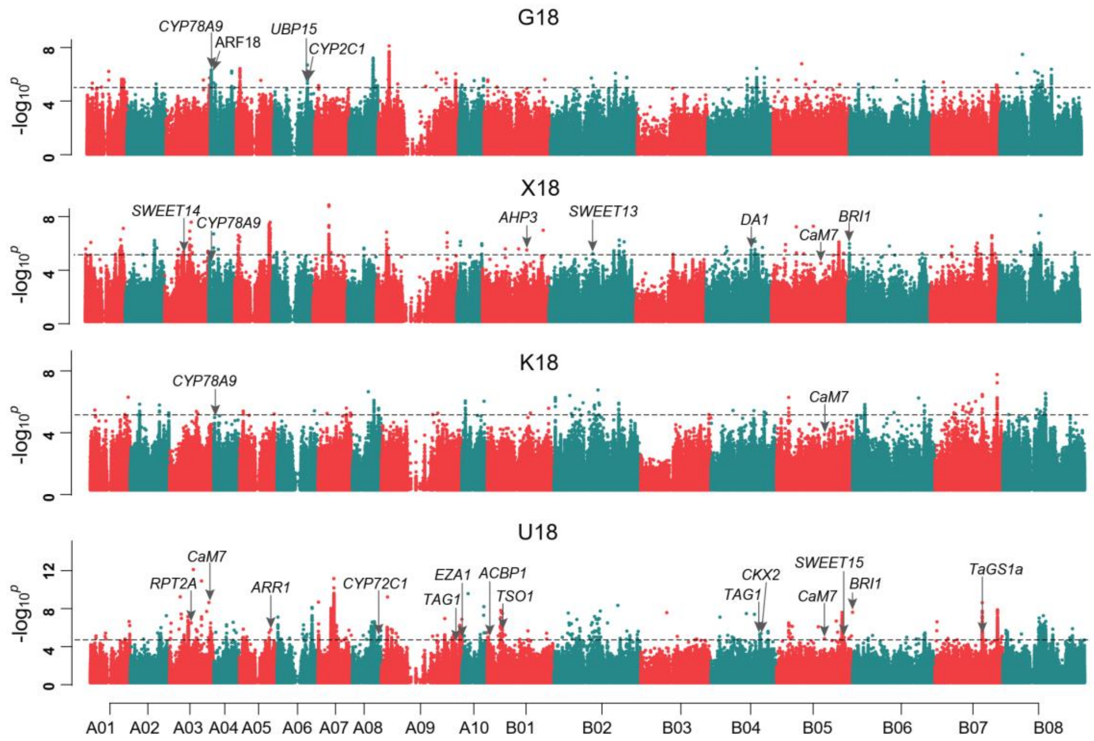

Supplementary Figure 28

GWAS of seed weight in *Brassica juncea* under four environments.

Candidate genes significantly associated with thousand seed weight are labeled in each environment. The significance threshold of -$\log_{10}p$ value was set at 5.

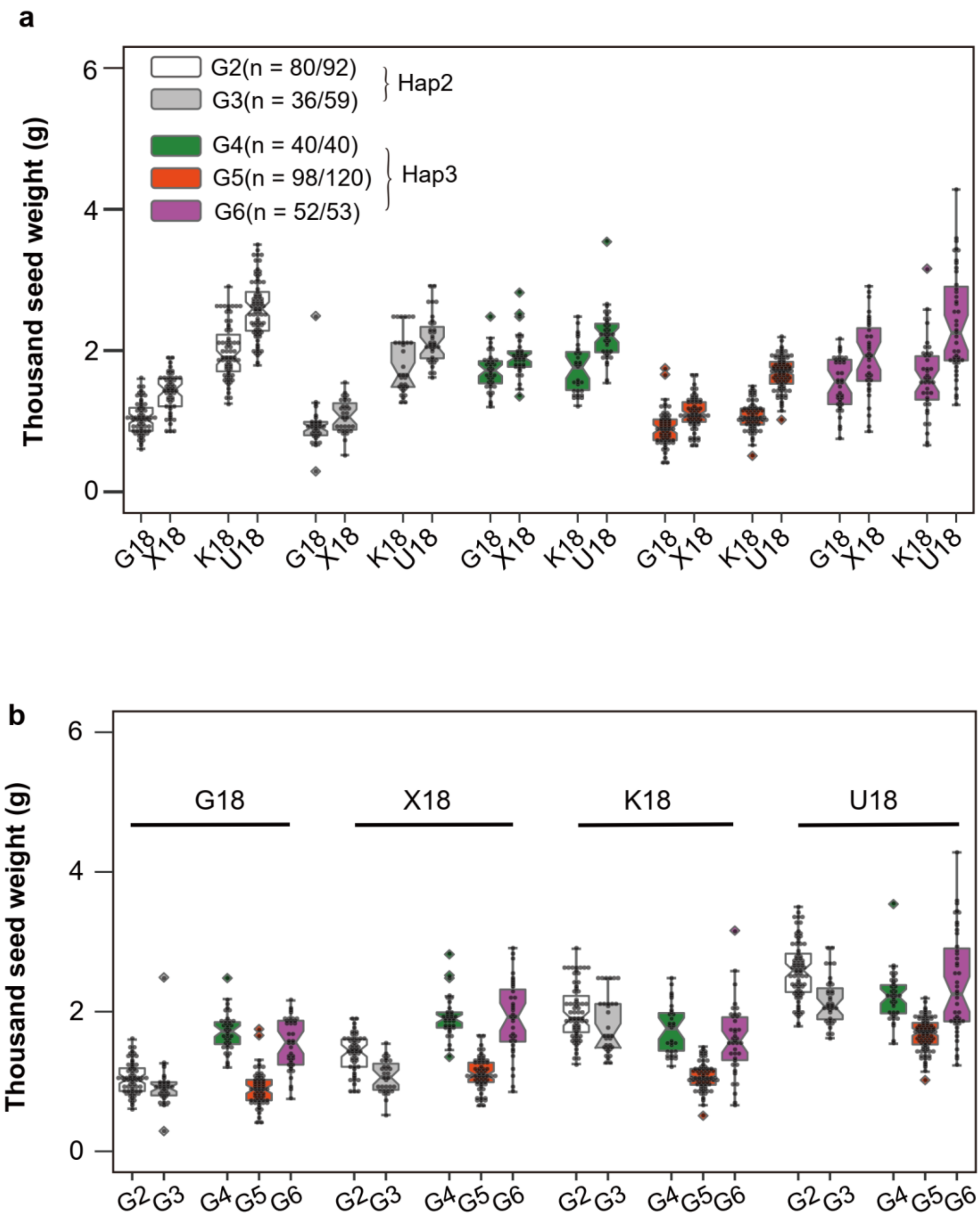

Supplementary Figure 29

Comparison of seed weight between Hap2 and Hap3 accessions in *CYP78A9* (BjuA04g00760S) and *CaM7* (BjuB05g28000S) under four environments.

a, Comparison of thousand seed weight among four different environments for accessions of each group. **b**, Comparison of thousand seed weight among five group accessions under each environment. Box edges represent the 0.25 and 0.75 quantiles, respectively, with the median values shown by bold lines. Whiskers extend to data no more than 1.5 times the interquartile range, and remaining data are indicated by dots.

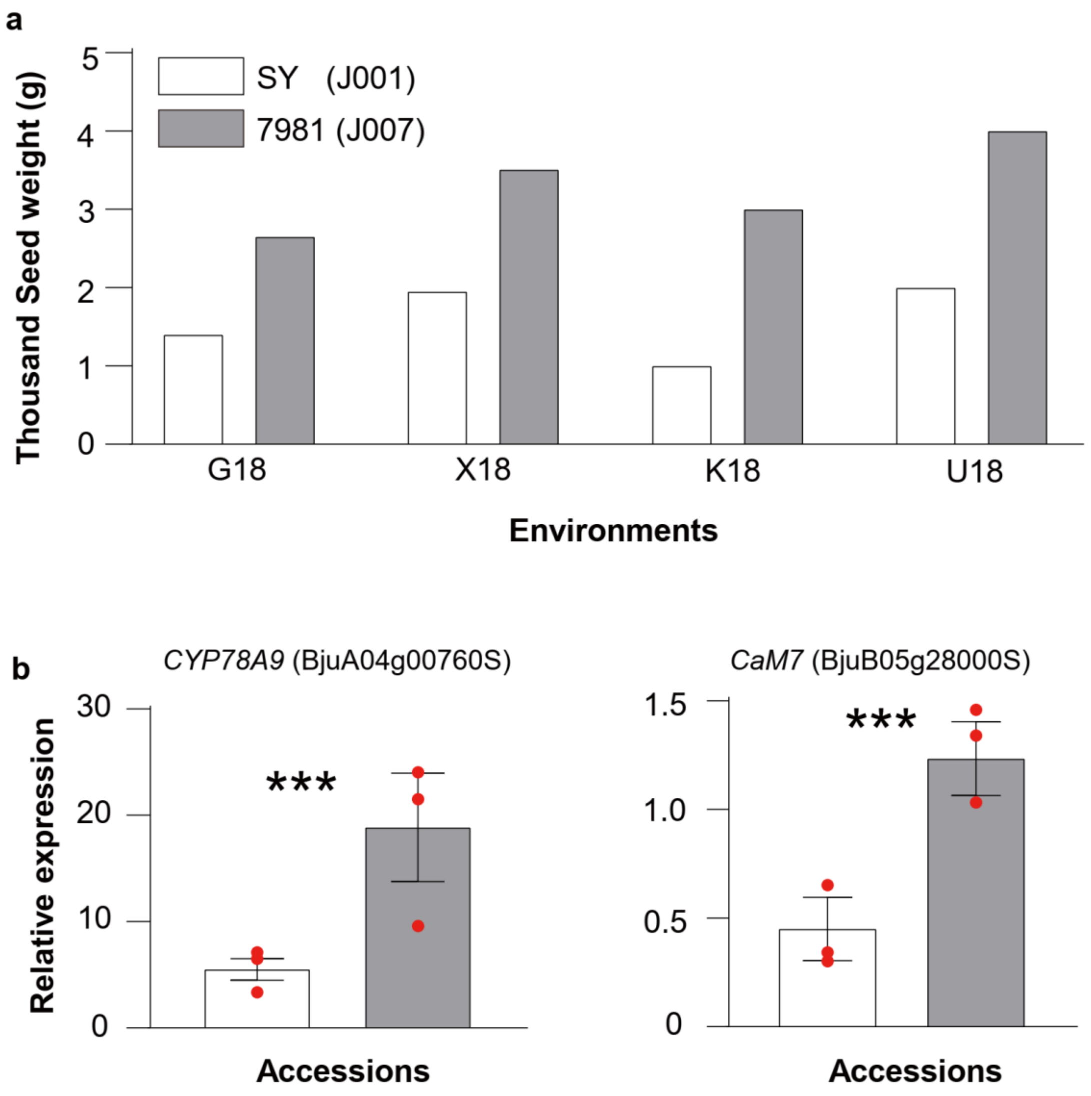

Supplementary Figure 30

Expression analysis of *CYP78A9* (BjuA04g00760S) and *CaM7* (BjuB05g28000S) in the small-seeded ‘SY’ and the large-seeded ‘7981’ of *Brassica juncea*.

a, The thousand seed weight of ‘SY’ and ‘7981’ under four different environments. **b**, Relative expression level of *CYP78A9* and *CaM7* in seeds 25 days post anthesis from the small-seeded ‘SY’ and the large-seeded ‘7981’. Reference gene: *HSP70-2*; n=3. Error bars, s.d. (n = 3). ***$P < 0.001$ (Two-sided t-test).

Source Data

Source Data 1 for the unprocessed gel in Supplementary figure 25a

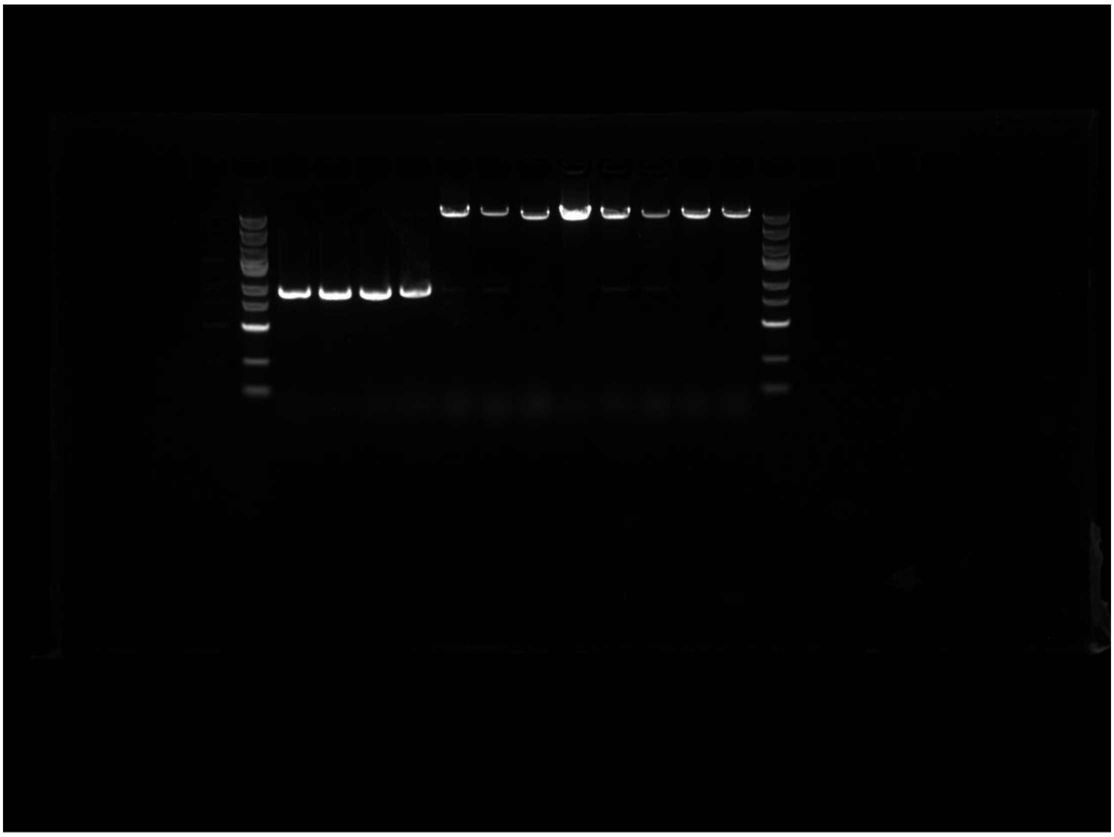

Source Data 2 for the unprocessed gels in Supplementary figure 27b

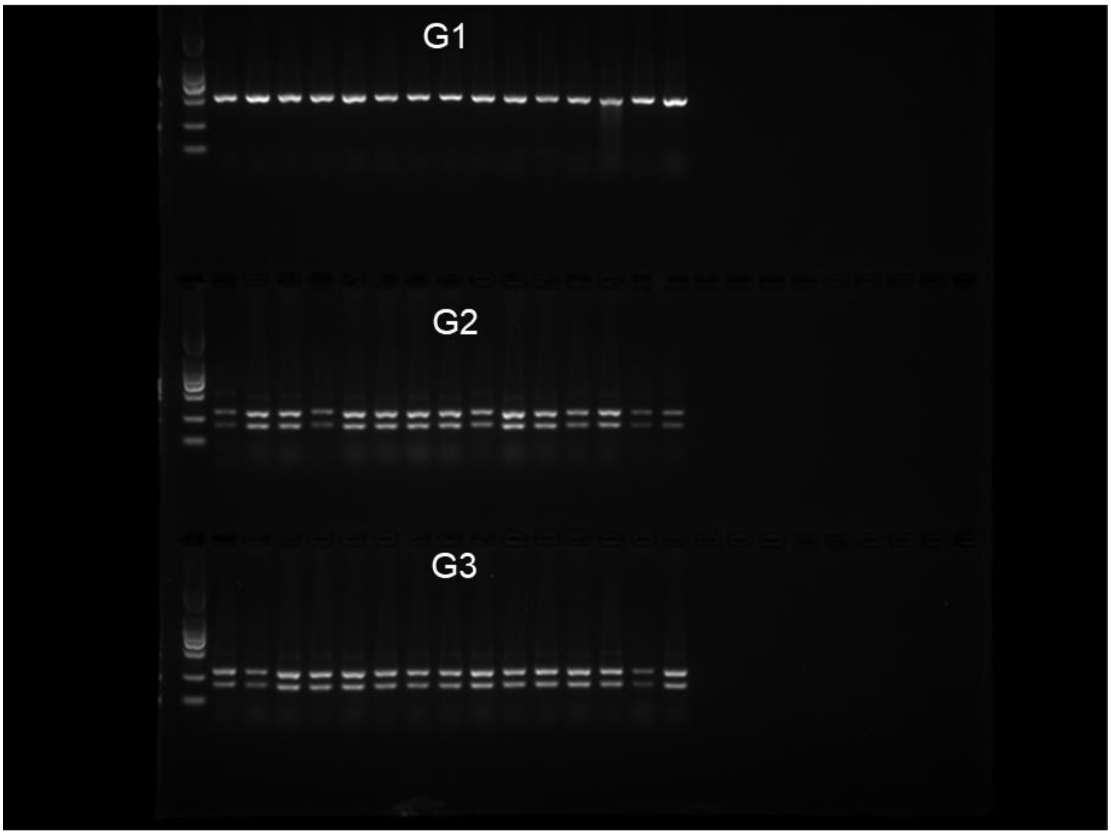

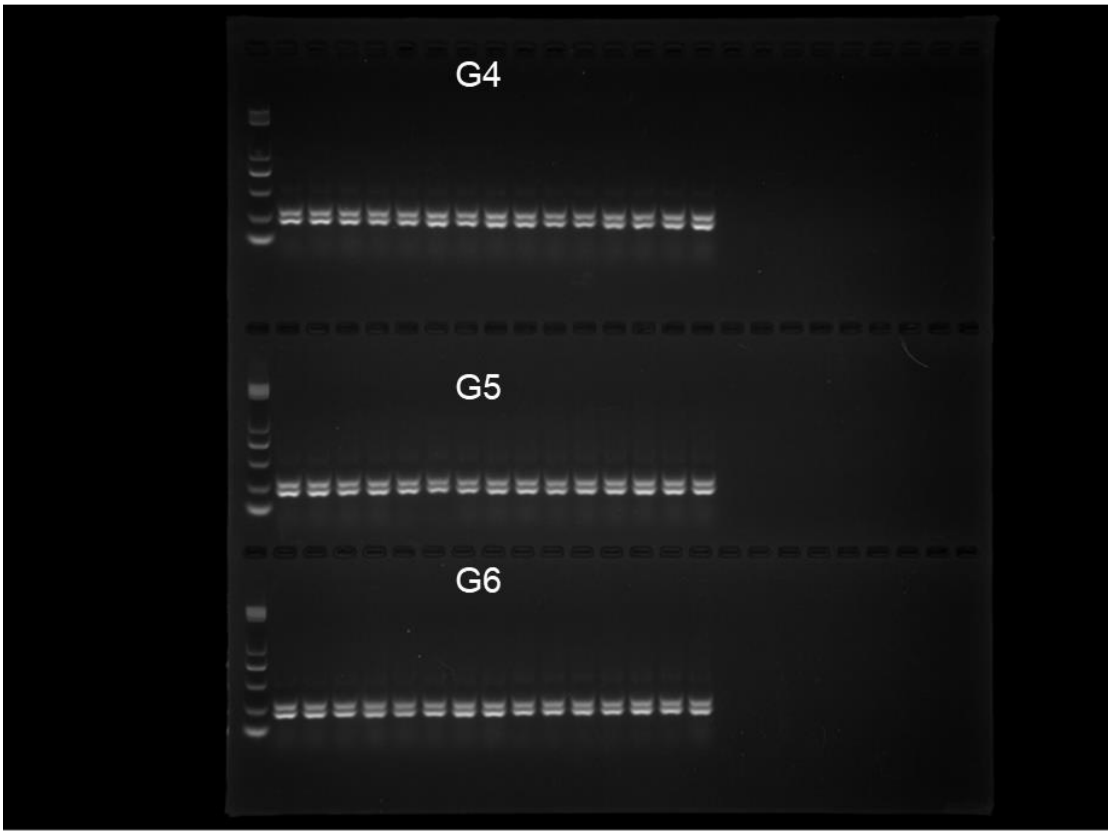

Supplementary Tables in a separate Excel file

Supplementary Table 1 Summary of genome sequencing strategy for *Brassica juncea* var. Sichuan Yellow.

(a) Illumina paired-end data

(b) PacBio sequencing data

(c) BioNano sequencing data

(d) Hi-C data

Supplementary Table 2 Statistics of *Brassica juncea* var. Sichuan Yellow genome.

Supplementary Table 3 Characteristics of the linkage map of *Brassica juncea* developed from a RIL population between Sichuan Yellow and Purple Leaf Mustard.

Supplementary Table 4 Comparison of the features of the assembled *Brassica juncea* Sichuan Yellow genome with previously reported genomes of *B. juncea* Tumida (V1.5) and Varuna.

Supplementary Table 5 List of genes for major phenotypic traits in *Brassica juncea.*

Supplementary Table 6 Comparison in genome assembly quality among *Brassica* genomes.

Supplementary Table 7 BAC validation for *Brassica juncea* var. Sichuan Yellow genome assembly.

Supplementary Table 8 Evaluation of *Brassica juncea* var. Sichuan Yellow genome using paired BAC end sequences.

Supplementary Table 9 Assessment of LTR-RT completeness of *Brassica* species using LAI.

Supplementary Table 10 Transposable elements annotation of published *Brassica* genomes.

Supplementary Table 11 Location of centromeres identified by mapping centromere-specific sequences in the *Brassica juncea* var. Sichuan Yellow genome.

Supplementary Table 12 Distribution of classified transposable elements in the centromeric and pericentromeric regions of *Brassica juncea* var. Sichuan Yellow genome.

Supplementary Table 13 Gene structure annotation of published *Brassica* genomes.

Supplementary Table 14 Gene function annotation of *Brassica juncea* var. Sichuan Yellow genome by using different databases.

Supplementary Table 15 Summary of RNA-Seq data in *Brassica juncea* var. Sichuan Yellow.

Supplementary Table 16 Summary of the 58 transcription factor gene families identified in the *Brassica juncea* var. Sichuan Yellow genome.

Supplementary Table 17 Noncoding RNA annotation in *Brassica juncea* var. Sichuan Yellow genome.

Supplementary Table 18 Information for the 480 *Brassica juncea* accessions used in this study.

Supplementary Table 19 Annotation of genomic variations in *Brassica juncea*.

Supplementary Table 20 Chromosomal distribution of SNPs and Indels in the 480 *Brassica juncea* accessions.

Supplementary Table 21 Major characteristics of six genetic groups in *Brassica juncea.*

Supplementary Table 22 Genomic polymorphisms in six genetic groups of *Brassica juncea*.

Supplementary Table 23 Genome-wide average LD decay in six genetic groups of *Brassica juncea.*

Supplementary Table 24 Summary of the genomic data of *Brassica rapa* and *B. nigra* used for phylogenetic analysis

Supplementary Table 25 Summary of the chloroplast and mitochondrial genome data of *Brassica rapa*, *B. nigra,* and *B. juncea* used in this study
(a) The chloroplast genomes of *B. rapa and B. nigra*
(b) The mitochondrial genomes of *B. rapa and B. juncea*

Supplementary Table 26 Gene flow among the six groups detected by *D*-statistic

analysis.

Supplementary Table 27 Genomic polymorphisms of subspecies in G5 accessions of *Brassica juncea*.

Supplementary Table 28 Summary of introgression rates in G4 group of *Brassica juncea*.

Supplementary Table 29 Phenotypic characteristics for flowering time and thousand seed weight in the 390 *Brassica juncea* accessions.

Supplementary Table 30 Flowering time candidate genes within the putative selective sweeps in *Brassica juncea*.

Supplementary Table 31 The summary of genes significantly associated with flowering time in *Brassica juncea*.

Supplementary Table 32 (a) Comparison between haplotypes of *SRR1* and *VIN3* in *Brassica juncea*.
(b) SNP information of *SRR1* and *VIN3* in *Brassica juncea*.

Supplementary Table 33 The summary of new genes significantly associated with flowering time in *Brassica juncea*.

Supplementary Table 34 Thousand seed weight candidate genes within the putative selective sweeps in *Brassica juncea*.

Supplementary Table 35 The summary of genes significantly associated with thousand seed weight in *Brassica juncea*.

Supplementary Table 36 (a) Comparison between haplotypes of *CYP78A9* and *CaM7* in *Brassica juncea.*
(b) SNP information of *CYP78A9* and *CaM7* in *Brassica juncea*.

Supplementary Table 37 The summary of new genes significantly associated with thousand seed weight in *Brassica juncea*.

Supplementary Table 38 Genomic regions detected by selective sweep scan for root enlargement in *Brassica juncea* ssp. *napiformis*.

Supplementary Table 39 Candidate genes for root enlargement in *Brassica juncea* ssp. *napiformis*

Supplementary Table 40 Genomic regions detected by selective sweep scan for stem swelling in *Brassica juncea* ssp. *tumida*.

Supplementary Table 41 Candidate genes for stem swelling in *Brassica juncea* ssp. *tumida*.

Supplementary Table 42 The primer sequences used in this study.
