# Supplementary material for: Genomic insights into the origin, domestication and diversification of Brassica juncea
Source: Nat Genet. 2021 Sep 6;53(9):1392–402. doi: 10.1038/s41588-021-00922-y (PMC8423626; doi:10.1038/s41588-021-00922-y)
Supplement: Supplementary file 2 — Reporting Summary [file 41588_2021_922_MOESM2_ESM.pdf]

nature research

Corresponding author(s): Zhongsong Liu and Wei Hua

Last updated by author(s): May 24, 2021

Reporting Summary

Nature Research wishes to improve the reproducibility of the work that we publish. This form provides structure for consistency and transparency in reporting. For further information on Nature Research policies, see our Editorial Policies and the Editorial Policy Checklist.

Statistics

For all statistical analyses, confirm that the following items are present in the figure legend, table legend, main text, or Methods section.

n/a	Confirmed	
☐	☒	The exact sample size (*n*) for each experimental group/condition, given as a discrete number and unit of measurement
☐	☒	A statement on whether measurements were taken from distinct samples or whether the same sample was measured repeatedly
☐	☒	The statistical test(s) used AND whether they are one- or two-sided<br>*Only common tests should be described solely by name; describe more complex techniques in the Methods section.*
☐	☒	A description of all covariates tested
☐	☒	A description of any assumptions or corrections, such as tests of normality and adjustment for multiple comparisons
☐	☒	A full description of the statistical parameters including central tendency (e.g. means) or other basic estimates (e.g. regression coefficient) AND variation (e.g. standard deviation) or associated estimates of uncertainty (e.g. confidence intervals)
☐	☒	For null hypothesis testing, the test statistic (e.g. F, t, r) with confidence intervals, effect sizes, degrees of freedom and P value noted<br>*Give P values as exact values whenever suitable.*
☐	☒	For Bayesian analysis, information on the choice of priors and Markov chain Monte Carlo settings
☒	☐	For hierarchical and complex designs, identification of the appropriate level for tests and full reporting of outcomes
☐	☒	Estimates of effect sizes (e.g. Cohen's *d*, Pearson's *r*), indicating how they were calculated

Our web collection on statistics for biologists contains articles on many of the points above.

Software and code

Policy information about availability of computer code

Data collection	(1) DNA sequencing: PacBio reads were collected from single-molecule real-time (SMRT) cells on PacBio Sequel instruments; Hi-C data and pair-end reads were collected from the Illumina HiSeq X Ten platform and Illumina HiSeq2000 platform; BioNano data were collected from BioNano Irys System using genomic DNA digested by Nt.BspQI.<br><br>(2) RNA-seq data were generated using Illumina HiSeq X Ten and its system software.
Data analysis	(1) Genome assembly: We used Jellyfish (v.2.2.9) to estimated the genome size. We used FALCON (falcon-kit==0.7), Sapace-longread (v.1.1), PBJelly (v.1.9.1), Chromonomer (v.1.07), QUIVER (v2.0.0), Plion (V1.22), BioNano Solve (V3.1), BWA (v.0.7.8), Juicebox (V1.9.8) for genome assembly. NOVOPlasty (V3.7) and Celera Assembler (V7.0) were used for chloroplast assembly and mitochondrial assembly, respectively.<br><br>(2) Genome annotation: We used Repeatmasker (v4.0.5), LTR_FINDER (V 1.0.5), RepeatScout (V1.0.5), RepeatModeler (v1.0.11), RepeatProteinMask (version 3.3.0,) Tandem Repeats Finder (TRF, version 4.07b), Augustus (version 2.5.5), Genscan (version 1.0), GlimmerHMM (version 3.0.1), Geneid (V1.4), SNAP (2013.11.29), Tophat (version 2.0.8), Cufflinks (version 2.1.1), PASA (v2.3.3), EvidenceModeler (V 1.1.1), tRNAscan-SE (V2.0), INFERNAL (v 1.1.3), WUblast (version 2.0), GeneWise (V2.4.1), InterProScan (v 5.32-71.0), BLAST (V2.2.28) for genome annotation.<br><br>(3) Assessment of genome completeness: We used BUSCO (v. 2) and CEGMA (v. 2.5) to evaluate the genetic integrity of the genome. LTR_retriever (v 2.8.7) was used to evaluate the integrity of the genome repeat sequence. LASTZ (V 1.02.00) software was used for BAC and BES evaluation. Heatmaps were generated using the JUICER-pre command, and visualized using Juicebox (V 1.9.8). BLASTP (v 2.2.6) was used to align the protein sequence of SY with previously reported Brassica genome, and MCScanX was used to build genome synteny between SY and other five Brassica subgenomes.<br><br>(4) Population structure analysis: We used BWA (v. 0.7.8), SAMtools (v.0.1.19), GATK (sentieon-genomics-201711), ANNOVARtool (version 2013-05-20) for SNP calling and annotation. ADMIXTURE (v.1.23) was used for population genetic structure analysis. IQ-TREE v1.6.6 was used

to construct phylogenetic tree. GCTA (V 1.26.0) was used for principal component analysis. The population relatedness and migration events were inferred using TreeMix (V 1.13). Arlequin (v.3.5.2.2) was used to calculate Nucleotide diversity (π) and fixation index (Fst). PopLDdecay (v.3.40) was used to calculate the squared correlation coefficient (r2) between pairwise SNPs. Ka/Ks Calculator (v. 2.0) and SMC++ (V1.13) were used for estimation of divergent time.

(5) GWAS and Selective sweep analysis: We performed GWAS using GEMMA (V 0.98.1) program under the mixed-linear model; XP-CLR (V 1.0) was used for selective sweep analysis.

For manuscripts utilizing custom algorithms or software that are central to the research but not yet described in published literature, software must be made available to editors and reviewers. We strongly encourage code deposition in a community repository (e.g. GitHub). See the Nature Research guidelines for submitting code & software for further information.

Data

Policy information about availability of data

All manuscripts must include a data availability statement. This statement should provide the following information, where applicable:

- Accession codes, unique identifiers, or web links for publicly available datasets
- A list of figures that have associated raw data
- A description of any restrictions on data availability

The genome sequence and annotation data for B. juncea var. Sichuan Yellow, the re-sequencing data for 480 B. juncea accessions, and transcriptome data are accessible under NCBI BioProject numbers PRJNA615316. Functional annotation of the SY genome used the SwissProt (ftp.uniprot.org/pub/databases/uniprot/current_release/knowledgebase/complete/uniprot_sprot.fasta.gz), NR (ftp.ncbi.nlm.nih.gov/blast/db/FASTA/nr.gz) and KEGG (release 53, https://www.genome.jp/kegg/brite.html) databases. Seed of accessions used, phenotype data, and sequences of the chloroplast and mitochondrial genomes reported here are available from the corresponding authors upon request. Source data are provided with this paper.

Field-specific reporting

Please select the one below that is the best fit for your research. If you are not sure, read the appropriate sections before making your selection.

☒ Life sciences ☐ Behavioural & social sciences ☐ Ecological, evolutionary & environmental sciences

For a reference copy of the document with all sections, see nature.com/documents/nr-reporting-summary-flat.pdf

Life sciences study design

All studies must disclose on these points even when the disclosure is negative.

Sample size	A panel of 480 mustard accessions were collected from 38 countries. It represents the four subspecies and samples all regions where mustard is spread. The 480 samples were clustered into six groups based on population structure. To better clarify the relationships of Brassica juncea accessions, 390 accessions with the genetic components of larger than 0.6 were retained for the further analysis.<br>Sample size for linkage map analysis was determined based on 172 recombinant inbred lines individuals.
Data exclusions	For population genomic analyses, we excluded 90 Brassica juncea accessions with the genetic components of less than 0.6 based on the population structure analysis.
Replication	Phenotyping was performed in four locations with two replicates at each location.<br>For RNA-Seq experiment, we used two biological replicates.<br>The PCR experiments were repeated independently for three times with similar results.<br>For all experiments, at least two replication were repeated and succeed.
Randomization	The 390 samples were clustered into six groups based on the result of population structure, PCA and phylogenetic tree.
Blinding	The investigators were blinded to the group allocations.

Reporting for specific materials, systems and methods

We require information from authors about some types of materials, experimental systems and methods used in many studies. Here, indicate whether each material, system or method listed is relevant to your study. If you are not sure if a list item applies to your research, read the appropriate section before selecting a response.

Materials & experimental systems

n/a	Involved in the study
☒	☐ Antibodies
☒	☐ Eukaryotic cell lines
☒	☐ Palaeontology and archaeology
☒	☐ Animals and other organisms
☒	☐ Human research participants
☒	☐ Clinical data
☒	☐ Dual use research of concern

Methods

n/a	Involved in the study
☒	☐ ChIP-seq
☒	☐ Flow cytometry
☒	☐ MRI-based neuroimaging
