# Supplementary material for: Genomic insights into the origin, domestication and diversification of Brassica juncea
Source: Nat Genet. 2021 Sep 6;53(9):1392–402. doi: 10.1038/s41588-021-00922-y (PMC8423626; doi:10.1038/s41588-021-00922-y)

Source Data for the gels in Extended Data figure 2a

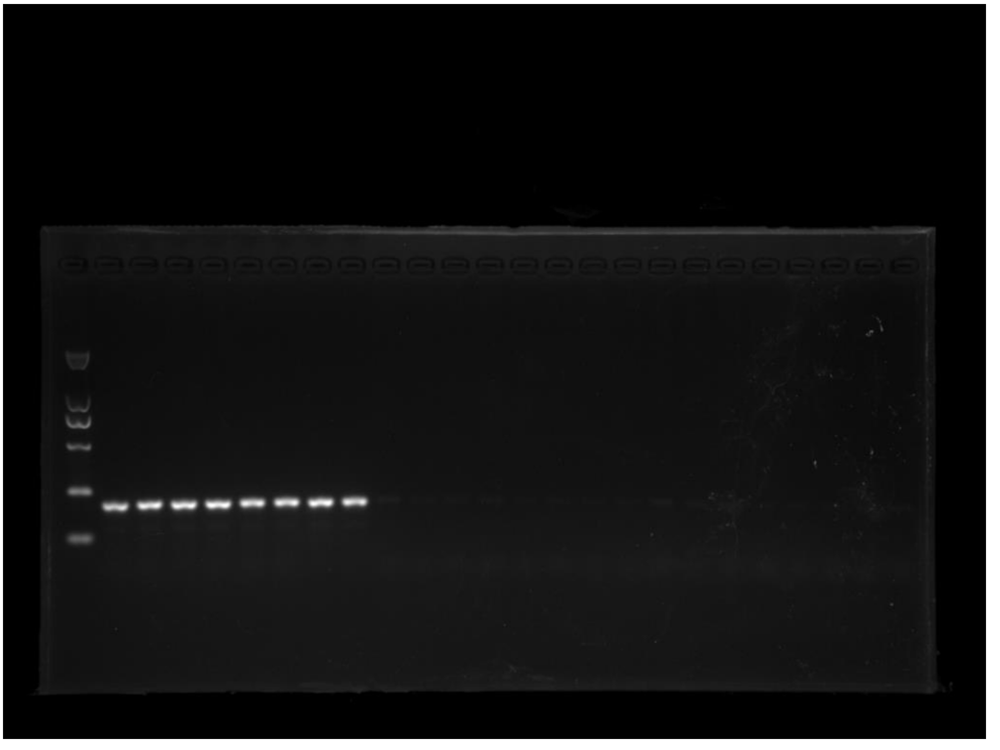

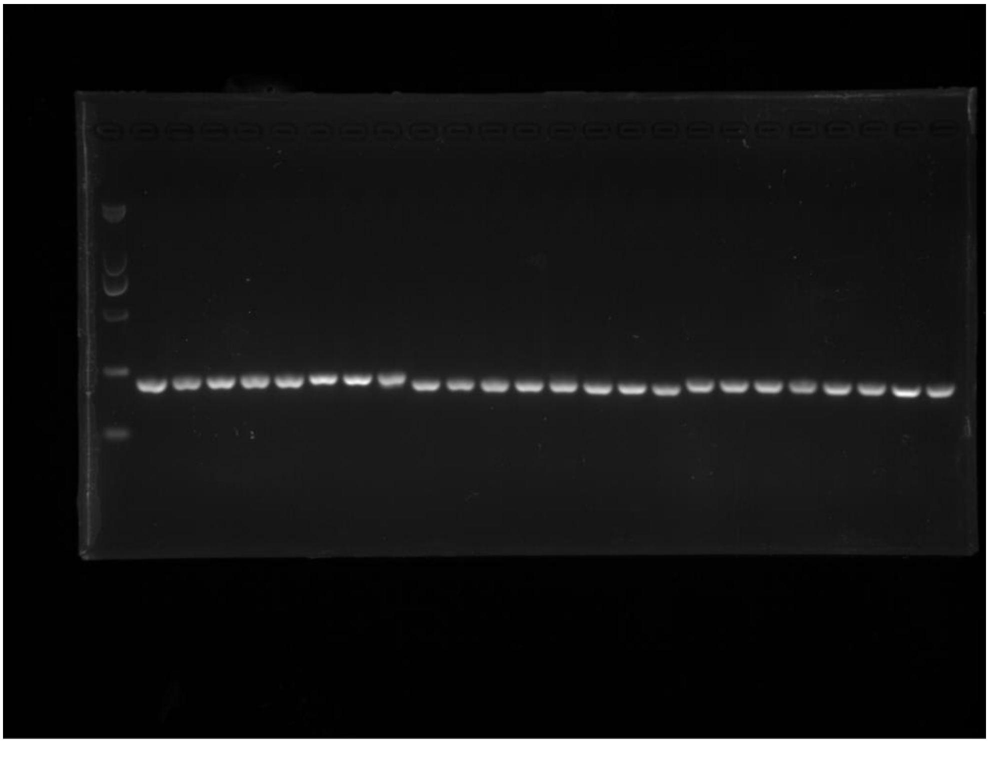

Source Data for the gels in Extended Data figure 2b

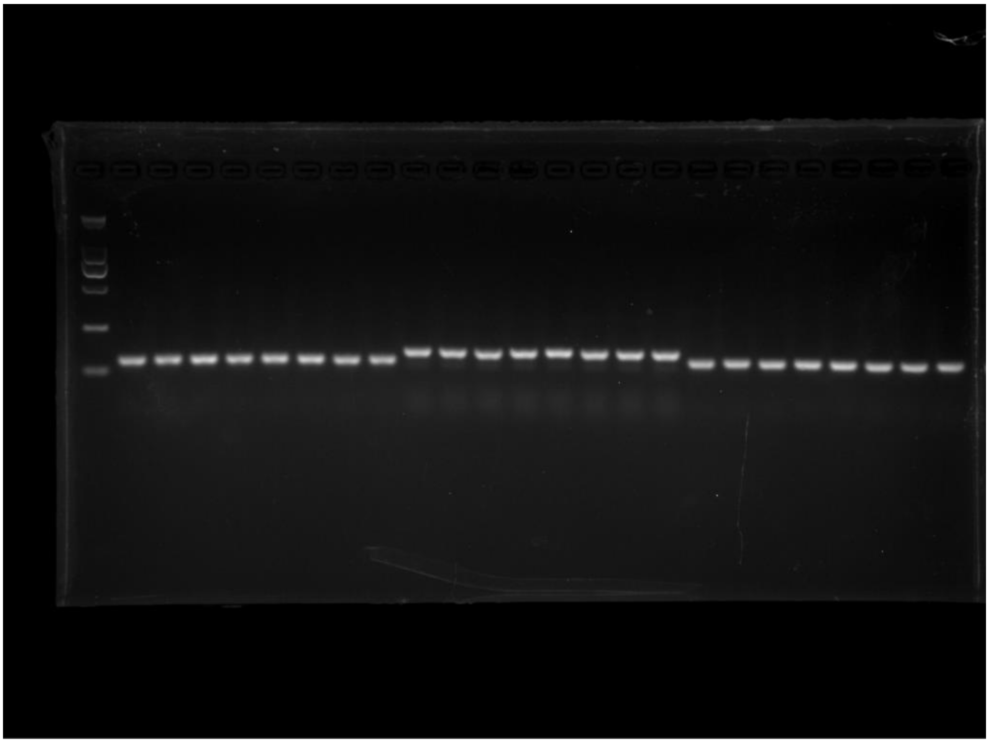

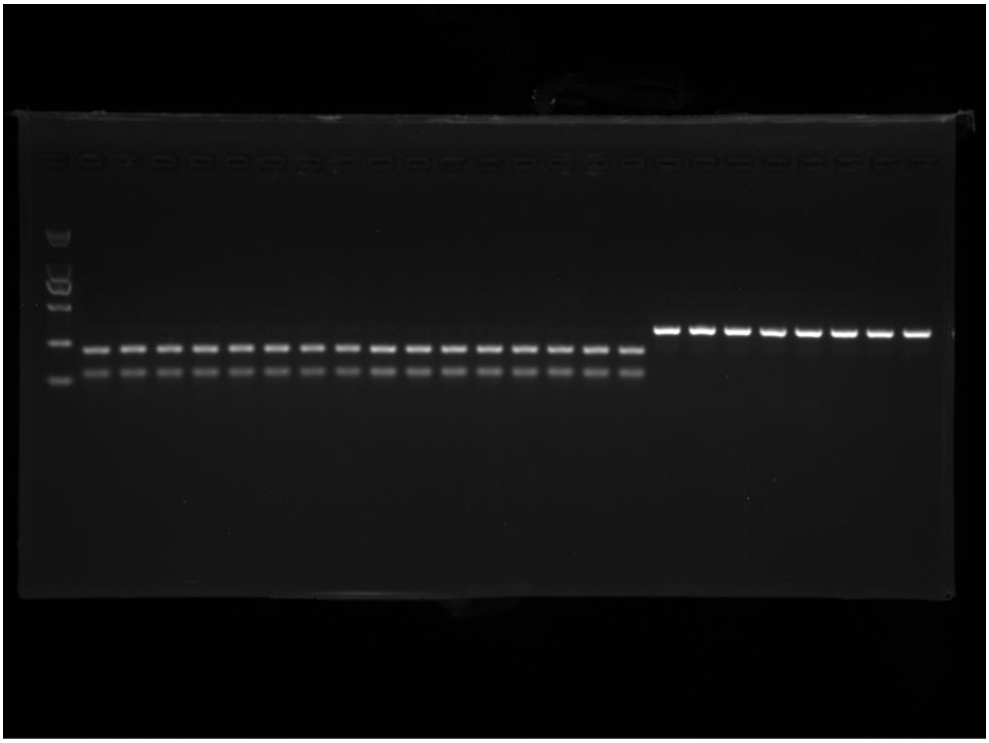

Supplement: Source Data Extended Data Fig. 2 — Unprocessed gels. [file 41588_2021_922_MOESM4_ESM.pdf]
